# Exercise, or exercise and diet for the management of polycystic ovary syndrome: a systematic review and meta-analysis

**DOI:** 10.1186/s13643-019-0962-3

**Published:** 2019-02-12

**Authors:** Chris Kite, Ian M. Lahart, Islam Afzal, David R. Broom, Harpal Randeva, Ioannis Kyrou, James E. Brown

**Affiliations:** 1 Aston Medical Research Institute, Aston Medical School, Aston University, Birmingham, B4 7ET UK; 2 Division of Translational and Experimental Medicine, Warwick Medical School, University of Warwick, Coventry, CV4 7AL UK; 3 Warwickshire Institute for the Study of Diabetes, Endocrinology and Metabolism (WISDEM), University Hospitals Coventry and Warwickshire NHS Trust, Coventry, CV2 2DX UK; 4 Centre of Applied Biological & Exercise Sciences, Faculty of Health & Life Sciences, Coventry University, Coventry, CV1 5FB UK; 5 School of Life and Health Sciences, Cell and Tissue Biomedical Research Group, Aston University, Aston Triangle, Birmingham, B4 7ET UK; 6 Faculty of Education Health and Wellbeing, University of Wolverhampton, Walsall Campus, Gorway Road, Walsall, WS1 3BD UK; 7 Centre for Active Living, University Centre Shrewsbury, University of Chester, Guildhall, Frankwell Quay, Shrewsbury, SY3 8HQ UK; 8 Academy of Sport and Physical Activity, Faculty of Health and Wellbeing, Sheffield Hallam University, Sheffield, S10 2BP UK

**Keywords:** Polycystic ovary syndrome, Exercise, Physical activity, Diet, Cardiovascular risk, Insulin resistance, Cardiorespiratory fitness

## Abstract

**Background:**

Typically, management of PCOS focuses on lifestyle changes (exercise and diet), aiming to alleviate symptoms, and lower the associated risk of type 2 diabetes and cardiovascular disease. Our objective was to analyse evidence on the effectiveness of exercise in the management of PCOS, when compared to (i) usual care, (ii) diet alone, and (iii) exercise combined with diet, and also exercise combined with diet, compared to (i) control or usual care and (ii) diet alone.

**Methods:**

Relevant databases were searched (June 2017) with no time limit for trial inclusion. Eligible trials employed a randomised or quasi-randomised design to measure the chronic effects of exercise, or exercise and diet in women with PCOS.

**Results:**

Searches returned 2390 articles; of those, 27 papers from 18 trials were included. Results are presented as mean difference (MD) and 95% confidence intervals (95% CI). Compared with control, exercise had a statistical effect on change from baseline fasting insulin (MD − 2.44 μIU/mL, 95% CIs − 4.24 to − 0.64; very low-quality evidence), HOMA-IR (− 0.57, − 0.99 to − 0.14; very low-quality evidence), total cholesterol (− 5.88 mg/dL, − 9.92 to − 1.83; low-quality evidence), LDL cholesterol (− 7.39 mg/dL, − 9.83 to − 4.95; low-quality evidence), and triglycerides (− 4.78 mg/dL, − 7.52 to − 2.05; low-quality evidence). Exercise also improved VO_2_ max (3.84 ml/kg/min, 2.87 to 4.81), waist circumference (− 2.62 cm, − 4.13 to − 1.11), and body fat percentage (− 1.39%, − 2.61 to − 0.18) when compared with usual care. No effect was found for change value systolic/diastolic blood pressure, fasting glucose, HDL cholesterol (all low-quality evidence), or waist-to-hip ratio. Many favourable change score findings were supported by post-intervention value analyses: fasting insulin (− 2.11 μIU/mL, − 3.49 to − 0.73), total cholesterol (− 6.66 mg/dL, − 11.14 to − 2.17), LDL cholesterol (− 6.91 mg/dL, − 12.02 to − 1.80), and VO_2_ max (5.01 ml/kg/min, 3.48 to 6.54). Statistically lower BMI (− 1.02 kg/m^2^, − 1.81 to − 0.23) and resting heart rate (− 3.26 beats/min − 4.93 to − 1.59) were also revealed in post-intervention analysis. Subgroup analyses revealed the greatest improvements in overweight/obese participants, and more outcomes improved when interventions were supervised, aerobic in nature, or of a shorter duration. Based on limited data, we found no differences for any outcome between the effects of exercise and diet combined, and diet alone. It was not possible to compare exercise vs diet or exercise and diet combined vs diet.

**Conclusion:**

Statistically beneficial effects of exercise were found for a range of metabolic, anthropometric, and cardiorespiratory fitness-related outcomes. However, caution should be adopted when interpreting these findings since many outcomes present modest effects and wide CIs, and statistical effects in many analyses are sensitive to the addition/removal of individual trials. Future work should focus on rigorously designed, well-reported trials that make comparisons involving both exercise and diet.

**Systematic review registration:**

This systematic review was prospectively registered on the Prospero International Prospective Register of Systematic Reviews (CRD42017062576)

**Electronic supplementary material:**

The online version of this article (10.1186/s13643-019-0962-3) contains supplementary material, which is available to authorized users.

## Background

Polycystic ovary syndrome (PCOS) is the most common endocrinopathy in reproductive-aged women, affecting 6–21% (depending on the applied diagnostic criteria) of this population worldwide [1–3]. PCOS is characterised by hyperandrogenism and/or chronic anovulation which can manifest with a range of symptoms (e.g., hirsutism, acne, oligomenorrhea, and infertility) [4] and is associated with increased risk of cardiometabolic disease, including hypertension, dyslipidaemia, insulin resistance (IR), and type 2 diabetes mellitus (T2DM) [5]. Moreover, PCOS is linked to increased psychological morbidity [e.g., increased risk of stress, depression, low self-esteem, poor body image, and reduced health-related quality of life (HRQoL)] [6, 7]. The exact PCOS aetiology is unknown, but increased adiposity is considered pivotal [8]. Indeed, almost 90% of women with PCOS are overweight or obese and even moderate weight loss (e.g., 5%) may result in clinically meaningful improvements in hyperandrogenism and menstrual regularity [9–13]. Also, women with PCOS often have more severe IR than weight-matched women without PCOS [14, 15], whilst their increased susceptibility to obesity [16] may further exacerbate IR and the accompanying metabolic [17, 18] and reproductive [10, 19] dysfunctions. As such, women with PCOS exhibit increased risk of impaired glucose tolerance and T2DM regardless of weight and age [20].

As there is currently no curative treatment for PCOS, management of overweight/obese women with PCOS focuses on weight loss through regular exercise and diet, aiming to alleviate its clinical manifestations and lower the related risk of T2DM and cardiovascular disease (CVD) [21]. Considering the benefits of exercise interventions in other IR populations independent of weight loss [22–24], incorporating moderate-intensity exercise in PCOS treatment may be particularly favourable. Existing evidence supports this; although most exercise trials in women with PCOS show little or no weight loss [5], exercise can have favourable effects on IR, body fat distribution, and CVD risk in these patients [25]. As the number of studies investigating the effects of exercise and diet in PCOS is increasing, it is important to summarise this body of evidence in order to better inform clinical practice. Therefore, this systematic review aims to analyse the evidence on the effectiveness of exercise compared to (i) control or usual care, (ii) diet alone, and (iii) exercise combined with diet, as well as the effectiveness of exercise combined with diet compared to (i) control or usual care and (ii) diet alone.

## Methods

This systematic review was prospectively registered on the Prospero International Prospective Register of Systematic Reviews (CRD42017062576) and is reported based on the guidelines of the Preferred Reporting Items for Systematic Reviews and Meta-Analyses (PRISMA) statement [26].

### Search methods for identification of studies

Table 1 presents the eligibility criteria for inclusion in this systematic review. Only trials with women of reproductive age who had received a PCOS diagnosis were eligible for inclusion. Eligible trials employed a randomised or quasi-randomised experimental (intervention) design to measure the chronic effects of exercise or exercise and diet in women with PCOS. We defined exercise as a potential disruption to homeostasis by muscle activity that is either exclusively, or in combination, concentric, eccentric, or isometric [27]. Accordingly, we accepted all methods of exercise training, including continuous aerobic exercise (e.g.*,* walking, jogging, or cycling); high-intensity interval training; resistance training; flexibility training; and yoga, Tai Chi, and Pilates. Trials were eligible if they had a pre-post design that compared at least two conditions, using either within-subject crossover design or between-subject comparison to a control/alternative treatment group. Studies, which included follow-up testing at least 1 month after completion of the intervention, were also included.

**Table 1:** Eligibility criteria for including studies in this systematic review

Inclusion criteria: 1. Study design: randomised controlled trials and quasi-randomised controlled trials. 2. Types of participants: reproductive-aged women with a diagnosis of polycystic ovary syndrome (PCOS) based on the National Institute of Health (NIH) diagnostic criteria (1990), the Rotterdam ESHRE/ASRM (2003) diagnostic criteria or the AE-PCOS Criteria (2006). We also included trials where the PCOS diagnosis had been verified by a general practitioner or specialist clinician. 3. Comparators: exercise vs usual care/control, exercise combined with diet vs usual care/control, exercise combined with diet vs diet only. Exercise combined with diet vs exercise only, exercise vs diet, exercise combined with pharmaceutical vs pharmaceutical. 4. All outcomes; expected outcomes included: primary outcomes, such as blood pressure, fasting blood glucose, insulin and lipid concentrations; and secondary outcomes, such as body mass index, cardiorespiratory fitness, testosterone, free androgen index and health-related quality of life measures. Exclusion criteria: 1. Study design: case studies, cross sectional and non-randomised controlled trials. 2. Types of participants: males, adolescent females, post-menopausal women, women without PCOS 3. Comparators: women with PCOS vs healthy controls, pharmaceutical vs exercise, pharmaceutical vs diet, diet vs diet, surgical vs any other condition.	

The databases searched were CENTRAL (in the Cochrane Library), PubMed, CINAHL, SCOPUS, EMBASE (via Web of Science), SportDiscus (via EBSCOhost), and PsycINFO (via OvidSP). A search algorithm was developed for PubMed (Additional file 1: Table S1), which was then modified for each database searched.

Searches were completed in June 2017 with no time limit specified for trial inclusion. Only fully published, peer-reviewed papers were included, whereas grey literature was not eligible. No language restrictions were placed on the search.

Initial searches were completed by one reviewer (CK), duplicate records were removed before title, and abstracts were screened independently by two reviewers (CK and IML). Subsequently, full-text eligibility screening was completed independently by two reviewers (CK and IML). Any disagreements on eligibility were resolved by discussion, whilst any unresolved disagreements by arbitration from a third reviewer (DRB).

Where multiple publications for the same trial were retrieved, they were linked together, and the earliest paper of the trial was used as the primary reference. The earliest paper was used as the reference only, and data were extracted from all papers with the most comprehensive available data included for each outcome. Data were extracted from eligible studies, and a summary of these findings are presented in Table 2. Trial data were combined in meta-analyses using Review Manager (RevMan 5.3.5, Copenhagen, Denmark).

**Table 2:** Characteristics of studies included in this systematic review

Study (design)	*N* randomised/analysed	Intervention duration (assessment points)	Participant characteristics (PCOS diagnostic criteria)	Intervention	Outcome measures
Almenning et al. [32] (RCT)	HIIT: 10/8 RT: 11/8 CON: 10/9	10 wks (baseline, 10 wks)	Age: 27.2 ± 5.5 y BMI: 26.7 ± 6.0 kg/m^2^ (Rotterdam)	HIIT frequency: 3 times/wk HIIT intensity: 2 d/wk, 4 × 4 mins 90–95% HR_max_/3 × 3 mins ~ 70% HR_max_. 1 d/wk, 10 × 1 min ‘all-out’/10 × 1 min rest. RT frequency: 3 times/wk RT sets × reps: 3 × 10 RT load: 75% 1-RM	HOMA-IR, FBG, FI, TG, TC, LDL-C, HDL-C VO_2_ max, RHR, BW, BMI, WC, BF%, FM, FFM, T, SHBG, FAI, hsCRP
Brown et al. [97] (RCT)	EX: 21/8 CON: 16/12	20–24 wks due to varying length of ramp-up phase (baseline, immediately post)	Age: 32.3 ± ns y BMI: 33.0 kg/m^2^ (NIH)	Exercise: 12-wk moderate-intensity intervention preceded by 8–12-wk ramp-up. Aerobic duration: ~ 228 min/wk (≤ 60 bouts) Aerobic intensity: 40–60% VO_2_ max	FBG, FI, HOMA-IR, TG, LDL-C, HDL-C, VO_2_ max, BW, BMI, WC, FT, SBP, DBP
Bruner et al. [98] (RCT)	EX + DIET: 7/7 DIET: 5/5	12 wks (baseline, 12 weeks)	Age: 30.7 ± 4.6 y BMI: 36.6 ± 6.0 kg/m^2^ (Rotterdam)	Exercise frequency: 3 times/wk Aerobic intensity: 70–85% HR_max_ Aerobic duration: 30 mins (+ 10-min warm-up) RT sets × reps: 2–3 × 10–15 RT load: not specified Diet: 1 h/wk of nutritional counselling	FI, QUICKI, VO_2_ max, BW, BMI, WC, T, SHBG, FAI
Guzick et al. [99] (RCT)	EX + DIET: 6/6 CON: 6/6	12 wks (baseline, 12 weeks)	Age: 31.7 ± 10.0 y BMI: ns (NIH)	Exercise frequency: 5 times/wk Exercise intensity: 1050–4200 kJ/wk Diet: VLCD (8 wks) with calories increased over final 4 wks (4200–5040 kJ/d). ‘Optifast’ used to supplement diet	FBG, FI, BW, WHR, T, SHBG, FT, LH, FSH
Hoeger et al. [100] (RCT)	LS + PLA: 11/6 PLA: 9/7 LS + MF: 9/5 MF: 9/5	48 wks (baseline, 24 wks, 48 wks)	Age: 28.5 ± 5.2 y BMI: 39.0 ± 6.1 kg/m^2^ (NIH)	Exercise programme: Individualised to achieve 150 min per week Diet: Individualised healthy balanced meal plan to achieve 500–1000 kcal deficit per day Metformin: 850 mg 2 times/day	BW, T, SHBG, FAI
Konopka et al. [102] (RCT)	EX: 12/12 CON: 13/13	12 wks (baseline, 12 wks)	Age: 35 ± 5.0 y BMI: 33.0 ± 5.0 kg/m^2^ (Rotterdam)	Exercise frequency: 5 times/wk Exercise intensity: 65% VO_2_ peak Exercise duration: 60 min	FBG, FI, HOMA-IR, BMI, BW, FM, FFM, E_2_
Nasrekani et al. [104] (RCT)	EX: 10/10 CON: 10/10	12 wks (baseline, 12 wks)	Age: 30.4 ± 5.9 y BMI: 28.3 ± 6.2 kg/m^2^ (Rotterdam)	Exercise frequency: 3 times/wk Exercise intensity: 40–65% HR_max_ Exercise duration: 25–30 min	VO_2_ max, BW, BMI, FSH, LH
Nybacka et al. [105, 106] (RCT)	EX: 19/17 EX + DIET: 19/12 DIET: 19/14	4 months (baseline, 4 months)	Age: 30.8 ± 5.2 y BMI: 36.0 ± 6.2 kg/m^2^ (Rotterdam)	Exercise programme: Individualised to meet individuals’ capacity, goals and interest. Diet: ≥ 600 kcal/day reduction maintaining 55–60% CHO, 25–30% fat and 10–15% protein.	FBG, FI, HOMA-IR, BW, BMI, WHR, BF%, FFM, T, SHBG, FT, E_2_, FSH, LH
Petranyi et al. [107] (QRCT)	LS + MF: 29/29 MF: 27/27	6 months (baseline, 6 months)	Age: 29 ± ns y BMI: 27.2 ± 6.9 kg/m^2^ (Rotterdam)	Exercise programme: recommendation to increase physical activity levels. Specifics unclear. Diet: low glycaemic index diet with caloric restriction for those who are obese. Metformin: 500 mg 3 times/day	BMI, WHR
Roessler et al. [34] (Randomised crossover)	EX: 8/7 CON: 9/7	16 wks (baseline, 8 wks, 16 wks)	Age: 31.7 ± 7.9 y BMI: 36.3 ± 7.2 kg/m^2^ (Rotterdam)	Exercise frequency: 3 times/wk (2 × cycle, 1 × walk) Exercise intensity: following 2-week ramp, cycling 20–180 s 80–100% HR_max_/rest 25–180 s 45–65% HR_max_. Walking 3–5 min 80–90% HR_max_/1 min 50–60% HR_max_. Exercise duration: 45 min (+ 10 min warm-up). Control: Group counselling sessions (2 h, 1 time/wk) focussing on barriers and motivation.	VO_2_ max, BW, BMI, WC
Sa et al. [36, 108] (RCT)	EX: 15/14 CON: 15/13	16 wks (baseline, 16 wks)	Age: 26.0 ± 5.0 y BMI: 32.8 ± 4.6 kg/m^2^ (Rotterdam)	Exercise frequency: 3 times/wk Exercise intensity: 60–85% HR_max_ Exercise duration: 40 min (+ 5 min)	SBP, DBP, FI, BMI, RHR, VO_2_ max, T, FSH, LH
Saremi et al. [109] (RCT)	EX: 11/11 CON: 11/11	8 wks (baseline, 8 wks)	Age: 35.2 ± 4.4 y BMI: 28.3 ± 4.3 kg/m^2^ (Rotterdam)	Exercise frequency: 3 times/wk Exercise intensity: 40–65% HR_max_ Exercise duration: 30 min	FBG, FI, HOMA-IR, TG, TC, LDL-C, HDL-C, VO_2_ peak, BW, BMI, BF%, WC, WHR
Saremi et al. [110] (RCT)	EX + PLA: 10/10 CON: 10/10 EX + CAL: 10/10	8 wks (baseline, 8 wks)	Age: 27.1 ± 5.1 y BMI: 25.5 ± 2.7 kg/m^2^ (Rotterdam)	Exercise frequency: 3 times/wk RT sets x reps: 1–2 × 15–20 RT load: 40–60% 1-RM	FBG, FI, HOMA-IR, TG, TC, LDL-C, HDL-C, BW, BMI
Stener-Victorin et al. [101, 103, 111–113] (RCT)	EX: 34/22 CON: 17/13 ACU: 33/24	16 wks (baseline, 16 wks, 32 wks)	Age: 30 ± 4.4 y BMI: 28.1 ± 7.3 kg/m^2^ (Rotterdam)	Exercise frequency: 3 times/wk Exercise intensity: HR ≥ 120 BPM Exercise duration: 30–45 min Low-frequency electroacupuncture: 14 × 30 min treatments over 16 wks	SBP, DBP, FBG, FI, HOMA-IR, TG, TC, LDL-C, HDL-C, BMI, WHR, T, FT, SHBG, FAI, LH, FSH, VO_2_ max, BMI, E_2_
Thomson et al. [33, 114–116] (RCT)	AET + DIET: 31/18 AET + RT + DIET: 33/20 DIET: 30/14	20 weeks (baseline, 10 wks, 20 wks)	Age: 29.3 ± 6.8 y BMI: 36.1 ± 4.8 kg/m^2^ (Rotterdam)	Exercise frequency: 5 times/wk (3 × aerobic, 2 × RT in combined exercise group) Aerobic intensity: 60–65% HR_max_ progressed to 75–80% HR_max_ by study end Aerobic duration: 25–30 min progressed to 45 mins by study end RT sets × reps: 3 × 12 RT load: 50–60% 1-RM progressed to 65–75% 1-RM after 2 wks Diet: energy restricted high protein diet (5000–6000 kJ/day)	SBP, DBP, FBG, FI, HOMA-IR, TG, TC, LDL-C, HDL-C, BW, BF%, FM, FFM, WC, T, SHBG, FAI, PCOS-Q
Turan et al. [117] (RCT)	EX: 16/14 CON: 16/16	8 wks (baseline, 8 wks)	Age: 24.5 ± 2.8 y BMI: 21.9 ± 3.5 kg/m^2^ (Rotterdam)	Exercise frequency: 3 times/wk Exercise duration: 50–60 min Aerobic intensity: 65–70% HR_max_ RT sets x reps: 1 × 15 RT load: 5–6 on RPE for RT scale	SBP, DBP, FBG, HOMA-IR, FI, TG, TC, HDL-C, LDL-C, BMI, WC, RHR, VO_2_ max, T, FT, E_2_, LH, FSH
Vigorito et al. [118] (RCT)	EX: 45/45 CON: 45/45	3 months (baseline, 3 months)	Age: 21.8 ± 2.1 y BMI: 29.4 ± 3.2 kg/m^2^ (Rotterdam)	Exercise frequency: 3 times/wk Exercise intensity: 60–70% VO_2_ max Exercise duration: 30 min	SBP, DBP, FBG, FI, TG, TC, LDL-C, HDL-C, VO_2_ max, RHR, BMI, WC, E_2_, T, FT, SHBG, FAI, LH, FSH, CRP
Vizza et al. [119] (RCT)	EX: 8/7 CON: 7/6	12 wks (baseline, 12 wks)	Age: 27 ± 5.0 y BMI: 37.8 ± 11.4 kg/m^2^	Exercise frequency: 4 times/wk (2 × RT, 2 home-based) RT sets × reps: 2–3 × 8–12 RT load: Progressed with strength gains Home-based: Callisthenics, 3 sets of 10 reps	FBG, FI, HOMA-IR, BW, BMI, WC, FM, FFM, BF%, hsCRP, T, SHBG, FAI, PCOS-Q, SF-36

Studies presented by lead author and year of publication. Design; *RCT* randomised controlled trial, *QRCT* quasi-randomised controlled trial. *N randomised* the number of participants randomised into each study arm at the study initiation, analysed is the number of participants included within the analysis, *HIIT* high-intensity interval training, *RT* resistance training, *CON* control group, *EX* exercise group, *DIET* dietary intervention, *LS* lifestyle, *PLA* placebo, *MF* metformin, *ACU* acupuncture, *AET* aerobic exercise training, *CAL* calcium supplementation. *Intervention duration* length of the duration, *assessment points* the time-points at which researchers have assessed outcome measures. Participant characteristics presented as mean ± standard deviation (SD) or median in one study [97] for age (in years, y) and BMI (kg/m^2^) at study entry, *ns* not specified. *Diagnostic criteria* the specific criteria used to confirm a PCOS diagnosis, *NIH* National Institute of Health (1990) diagnostic criteria, *Rotterdam* European Society for Human Reproductive and Embryology/American Society for Reproductive Medicine (2003). Outcome measures refers to the outcomes from each study that are relevant to this systematic review. *VO*_*2*_ *max* maximum oxygen uptake, *RHR* resting heart rate, *HDL-C* high-density lipoprotein cholesterol, *LDL-C* low-density lipoprotein cholesterol, *TC* total cholesterol, *TG* triglycerides, *FBG* fasting blood glucose, *FI* fasting insulin, *HOMA-IR* homeostatic assessment of insulin resistance, *QUICKI* quantitative insulin sensitivity check index, *FM* fat mass, *FFM* fat-free mass, *BF%* body fat percentage, *BW* body weight, *BMI* body mass index, *WC* waist circumference, *WHR* waist-to-hip ratio, *SHBG* sex hormone binding globulin, *FAI* free androgen index, *T* testosterone, *FT* free testosterone, *E*_*2*_ oestradiol, *LH* luteinising hormone, *FSH* follicle stimulating hormone, *SBP* systolic blood pressure, *DBP* diastolic blood pressure, *hsCRP* high-sensitivity C-reactive protein, *d* day, *mins* minutes, *wk* week, *reps* repetitions, *RM* maximum number of repetitions, *HRmax* maximum heart rate, *PCOS-Q* PCOS health-related questionnaire, *SF-36* Optum36-item Short Form Survey, *VLCD* very low calorie diet, *CHO* carbohydrate

All trial outcomes were considered for inclusion following the search, but the primary outcomes were those linked to CVD risk (e.g., blood pressure, lipids, and glucose). Secondary outcomes were cardiorespiratory fitness (CRF), anthropometric measures, androgen levels, pro-inflammatory markers, and psychosocial outcomes.

### Assessment of risk of bias in included studies

The Cochrane Collaboration’s tool for assessing risk of bias was used; and six specific domains (sequence generation, allocation concealment, blinding, incomplete outcome data, selective outcome reporting, and any other sources of bias) were assessed. Two reviewers (CK and IA) assessed risk of bias, and a third reviewer (IML) arbitrated conflicts not due to assessor error. The *Cochrane Handbook* recommendations [28] were followed, and each bias parameter was graded as either high, low, or unclear risk. We judged studies with > 20% of data missing as at a high risk of attrition bias. We considered studies with between-group baseline differences that may affect the outcome, less than 75% adherence in the intervention group, and contamination in the control group (i.e., control group participants engaged in exercise), as high risk of ‘other sources of bias’ [29]. In exercise trials, it is difficult to blind participants and researchers to the interventions resulting in a high risk of performance bias being made; this should not infer that the methodological quality of the trial is poor, but rather that the inevitable bias related to lack of blinding has been acknowledged by the reviewers. A risk of bias table is presented in Additional file 1: Table S2 and risk of bias summarised in the results (Fig. 2; Additional file 2: Figure S1).

### Strategy for data synthesis

Where data from ≥ 2 trials were available, pooled intervention effect estimates and their 95% confidence intervals (CIs) are presented. Meta-analytical methods for involving continuous outcomes assume that data are normally distributed; hence, data were excluded from the meta-analysis when they were clearly skewed, or results were reported with median and range values, and non-parametric tests used for analysis.

Outcomes across each trial were presented as continuous data and, based on the *Cochrane Handbook’s* recommendations [30], the random-effects method for meta-analysis was utilised to combine data [31]. Mean ± standard deviation (SD) data for either change from baseline to post-intervention or immediately post-intervention values were combined in a meta-analysis. The RevMan calculator was used to convert standard errors, CIs, or *t* values to SD where necessary. A priori, the analysis was based on change from baseline scores as it removed a component of between-person variability [30]; immediately, post-intervention analysis was also included so as to nullify the effect of selective reporting, but also to better indicate whether there was a treatment effect regardless of baseline values. Mean difference (MD) was used where trials reported the same outcome using the same scale. Where scales varied, units of measurement were converted to the most common measure [e.g., fasting insulin (FI) converted from pmol/L to μIU/mL]. If this was not possible, standardised mean difference (SMD) was used. Immediately, post-intervention values were also assessed, and their data reported. If trials contained more than one eligible intervention arm [32, 33], outcome data from both groups were combined using methods recommended by Deeks et al. [30]. If an included trial used a crossover design [34], then only data up to the point of crossover were used.

We used the Grades of Recommendation, Assessment, Development, and Evaluation (GRADE) approach [35] to assess the quality of the evidence for our primary outcomes: systolic and diastolic blood pressure, blood glucose, FI, homeostatic model assessment of insulin resistance index (HOMA-IR), total cholesterol (TC), low-density lipoprotein cholesterol (LDL-C), high-density lipoprotein cholesterol (HDL-C), and triglycerides. GRADEpro GDT software was used to develop the ‘Summary of findings’ table, and two review authors (IML and CK) graded the quality of the evidence for each outcome. We did not downgrade based on lack of blinding alone due to difficulties of blinding participants and exercise supervising personnel. We downgraded based on risk of bias only if a lack of blinding was accompanied by additional high risks of bias (e.g., selection bias and incomplete outcome reporting).

### Investigation of heterogeneity

The *I*^2^ statistic was used to evaluate the heterogeneity of results for each outcome, across studies. Although not a measure of absolute heterogeneity, the *I*^2^ describes the percentage of variability in the point estimates that is due to heterogeneity rather than sampling error [30]. We interpreted heterogeneity as 0–40% ‘might not be important’, 30–60% ‘may represent moderate heterogeneity’, 50–90% ‘may represent substantial heterogeneity’, and 75–90% ‘considerable heterogeneity’ [30]. The importance of the observed *I*^2^ value depends on the magnitude and direction of effects, as well as the strength of evidence for heterogeneity. Visual inspection of forest plots was completed, and statistical heterogeneity assumed if there was little or no overlap of CIs for the results of individual studies. When evidence of at least substantial heterogeneity was present, its source was investigated by study population groups—the trial that represented the largest outlier was removed from the analysis and the *I*^2^ was re-evaluated. If heterogeneity was not reduced, it was also assessed in subgroup analyses.

### Assessment of reporting biases

To investigate publication bias, if there were ≥ 10 trials included in an analysis, we used a funnel plot to explore the possibility of small study effects—a tendency for smaller studies to report larger beneficial effects. This was only completed for one outcome (BMI) because when there are fewer studies (< 10), the power of tests is too low to distinguish chance from real asymmetry [30].

### Subgroup analysis

Where there were data from ≥ 2 studies, analyses of subgroups was conducted. Study characteristics analysed were body mass index (BMI) upon study entry (BMI ≤ 24.9 kg/m^2^, 25.0–29.9 kg/m^2^ or ≥ 30.0 kg/m^2^), intervention type (aerobic exercise, resistance training, or combination of the two), intervention duration (≤ 12 weeks or > 12 weeks), and intervention delivery format (supervised, unsupervised, or mixed delivery). Outcome data were separated by subgroup, and subtotal summary statistics were presented. The available data were insufficient to complete three of the sub-analyses (exercise intensity, combined treatments, and behaviour change components) outlined in the original protocol, but findings have been reported qualitatively where available.

### Sensitivity analysis

Sensitivity analyses were completed on outcomes where an effect was observed to assess the effect of removing small sample size studies (*n* < 30 total participants) and those with high overall bias risk. Due to the nature of the interventions, performance and detection bias were removed from the reviewers’ judgement. All studies exhibited at least one domain where risk of bias was unclear, so only those with at least one domain where risk of bias was deemed to be high were removed.

## Results

### Description of included studies

#### Search results

**Fig. 1:**
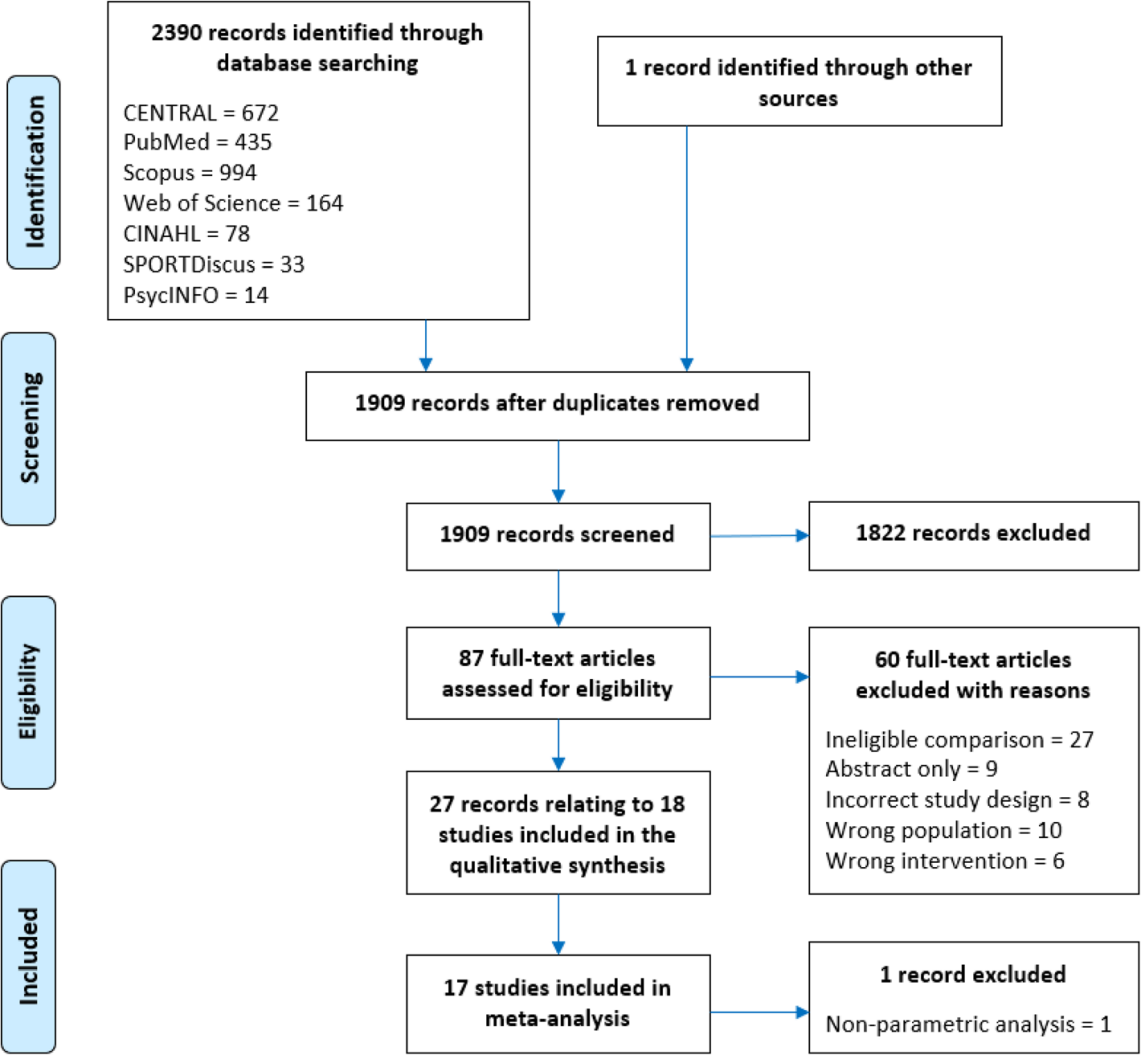
Preferred Reporting Items for Systematic Reviews and Meta-analyses (PRISMA) flow diagram

In total, 2390 articles were identified from the database searches; we were also sent one additional article after requesting further information from another author [36]. After removing duplicates, 1908 articles were screened for eligibility based on title and abstract. A total of 87 full-text articles were retrieved for detailed eligibility evaluation, and 60 of these were excluded [37–96] with reasons detailed in Fig. 1 and Additional file 1: Table S3.

Following exclusion, 27 met the inclusion criteria [32–34, 36, 97–104, 106–119]. However, these publications were based on 18 trials, since four trials had multiple publications, namely Stener-Victorin et al. [111] four additional papers [101, 103, 112, 113]; Thomson et al. [33] three additional publications [114–116]; Nybacka et al. [105] one additional publication [106]; and Sa et al. [108] one additional publication [36].

One study was excluded from the meta-analysis [97] because data were reported as median and range values (attempts to contact the author were unsuccessful).

#### Eligible studies design and attrition

Of the 18 included trials, 16 were randomised controlled clinical trials (RCTs), whilst one trial had a quasi-RCT [107] and another a randomised crossover [34] design.

Twelve trials compared exercise to usual care or minimal intervention [32, 34, 97, 102, 104, 108–111, 117–119]. Three trials each compared combined exercise and diet with diet only [33, 98, [105]], and exercise and diet combined with usual care [99, 100, 107]. Only one trial [105]investigated exercise versus diet and exercise versus exercise and diet combined. The total number of participants included within the trials were 758 (exercise/intervention, *n* = 230; control, *n* = 257; combined treatment arms, *n* = 174; and diet alone, *n* = 54). In addition, 43 participants were included in ineligible arms, i.e., pharmacological arm [100, 107, 110] and low-frequency electroacupuncture [111].

Eight trials (44%) did not report any attrition [98, 99, 102, 104, 107, 109, 110, 118]. Where reported, attrition ranged from 6% [117] to 50% [33] with a median value of 19.5%; five trials (28%) reported attrition over 20% [32, 33, 97, 100, 105]. Reasons for exercise dropouts included non-exercise related injury [32–34, 97, 119], pregnancy [32–34, 100, 119], time [33, 34, 97], work/family commitments [32, 33, 119], personal reasons [33, 105, 108, 111], medical grounds [97, 105, 111], and relocation [33]. Two trials excluded participants because adherence to intervention was < 75% [117] or failure to comply with study requirements [33].

#### Participant characteristics of included studies

Participant characteristics are presented in Table 2. Included trials used a range of criteria to diagnose PCOS as presented in Additional file 1: Table S4, with three trials [97, 99, 100] using the NIH diagnostic criteria [120], whereas 14 [32–34, 98, 102, 104, 105, 107–111, 117, 118] used the Rotterdam consensus criteria [121]. One trial confirmed the PCOS diagnosis via participants’ general practitioner/specialist [119], but criteria used were unclear. No trials specified use of the AE-PCOS definition [122].

Participants with T2DM, fasting hyperglycaemia, or glucose intolerance were explicitly excluded in nine trials (50%) [33, 34, 97, 98, 102, 109, 111, 117, 118], and nine trials also excluded participants with any diagnosed CVD [33, 34, 98, 99, 109, 111, 117–119]. Another prerequisite in seven trials (39%) was the activity status of participants upon enrolment, namely a sedentary lifestyle and no recent participation in an exercise intervention had to be apparent [32, 33, 97, 102, 104, 109, 119].

#### Intervention and comparison details

Fourteen trials (74%) assessed the effectiveness of an exercise-only intervention and six trials (32%) assessed a combined exercise and dietary intervention. Moreover, 14 trials (74%) included intervention arms consisting of aerobic exercise only, and a further three (16%) combined aerobic exercise with resistance training [33, 98, 117]. Of those incorporating aerobic exercise (*n* = 17, 94%), 11 trials (61%) specified either walking, brisk walking, or jogging [32–34, 97–99, 104, 105, 108, 109, 111] and seven (39%) incorporated static cycling either on its own or as part of a wider intervention [32, 34, 97, 98, 102, 111, 118]. A trial each incorporated elliptical training [97], step training [117] or swimming [105]. Five trials (28%) [32, 97, 98, 105, 111] allowed participants to self-select modality from those listed above, whereas two trials [100, 107] allowed participants to self-select a modality but without stating the choices. Three trials (16%) had arms that were resistance training only [32, 110, 119]. However, in one trial, the type of exercise was unclear [107].

The modal training session frequency was three per week in 10 trials (56%) [32, 33, 98, 104, 108–111, 117, 118]. Five sessions per week were prescribed in three (17%) trials [33, 99, 102], whereas in another trial [119] four sessions per week were set. Of the remaining four trials, one specified a weekly physical activity (PA) time target of 150 min per week [100], one trial set an exercise dose of 14 kcal/kg/week [97], and two did not specify training frequency or volume [105, 107].

Eight trials (44%) set aerobic exercise intensity using a percentage of the maximum heart rate (HR_max_) [32–34, 98, 104, 108, 109, 117] or maximal oxygen uptake (VO_2_ max) [97, 102, 118]. One trial specified that heart rate (HR) was set at ≥ 120 beats/min [111]. Three trials using resistance training prescribed intensity based on a percentage of 1-repetition maximum (either 40–60% [110] or 50–75% [32, 33]). One resistance training intervention set intensity using a rate of perceived exertion of 5–6 out of 10 [117]. Six trials did not specify the intensity of the intervention [98–100, 105, 107, 119]. Four trials increased the intensity as the intervention progressed [10–104, 106–110].

Eleven trials (61%) prescribed session durations of 1 hour or less (≤ 30 min [32, 34, 104, 109, 118], > 30–60 min [97, 102, 108, 111, 119], or 20–30 to 45 min [33]). Only one trial consisted of training sessions of > 60 min [98]. Hoeger and colleagues [100] specified 150 min as a weekly target, whereas another trial used a target distance of 10 miles per week [99]. Four trials did not specify timings for their intervention [11, 105, 107, 110].

In ten trials (56%), participants were fully supervised in all exercise sessions [34, 98, 102, 104, 105, 108–110, 117, 118], whilst two (11%) used a mixed approach with some supervised sessions [32, 119] and one (6%) was unsupervised with support provided weekly by telephone [111]. The remaining five trials (26%) did not report supervision status.

Six trials (33%) incorporated a dietary component. Five of these trials (28%) specified either a daily caloric target [33, 99], a reduced caloric intake [105, 107], or an individualised caloric deficit [100]. The other of these trials [98] used weekly nutritional counselling sessions to educate participants on a range of nutritional topics.

Thirteen trials (72%) had a control arm (Table 2) [32, 34, 96. 98, 101, 103, 107, 109–111, 117–119]. Three of these trials offered participants the intervention [34, 99] or a 1-month gym membership [32] upon completion of the trial (wait-list control). Three of the remaining trials (17%) used a diet-only arm as their comparison group [33, 98, 105], one trial used a placebo [100], and another used metformin treatment only [107].

#### Characteristics of the outcome measures

All studies assessed participants at baseline and immediately post-intervention (Table 2), whilst two trials incorporated an additional midway assessment [33, 100], one trial added a follow-up assessment 16 weeks post-intervention [111], and another trial assessed at baseline, crossover, and immediately post-intervention [34]. No post-intervention follow-up analysis was possible due to lack of studies.

Seven trials (39%) stated the sample size calculation methods [32, 97, 105, 108–111, 118], although only five (28%) of those reported the outcome upon which their calculations were based [32, 97, 108, 111]. The primary outcomes (used in sample size calculation) were HOMA-IR [32], VO_2_ peak [108], total testosterone [111], insulin sensitivity [97], and BMI [105]. Only three trials stated recruitment targets [32, 108, 111]; all three trials achieved their sample size calculated target. The outcomes included in each trial are provided in Table 2.

### Assessment of risk of bias in included studies

**Fig. 2:**
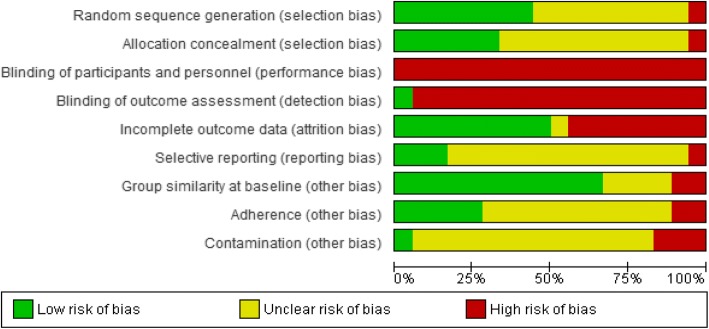
Review of authors’ judgement of each methodological quality item from the Cochrane Risk of Bias tool, presented as a percentage across all 18 included studies

The authors’ risk of bias judgements are presented in the risk of bias graph (Fig. 2), whilst further details are included in Additional file 2: Figure S1 and Additional file 1: Table S2.

Only four trials (22%) were judged to have a low risk of selection bias, using appropriate methods to generate their randomisation sequence and conceal allocation [32, 97, 117, 119]. One trial was judged to be at high risk of selection bias [108] because five participants were allocated to the control group based upon their geographical location. The remaining trials were judged to have an unclear risk of selection bias due to insufficient reporting of sequence generation or allocation concealment methods. Due to the nature of the interventions, all trials were judged to be at a high risk for performance bias. Only one trial had a low risk for detection bias [118]; the remaining trials were judged to be at a high risk of this bias due to not stating whether outcome assessors were blinded to participant allocation. One trial [32] used an independent, and blinded, assessor for evaluation of only one outcome (flow-mediated dilation).

Eight trials (44%) were judged to be high risk for attrition bias because participant withdrawal rates were > 20% [33, 97, 100, 105, 111], incomplete data due to lab error [98], inappropriate handling of missing data (i.e., last observation carried forward) [119], and only a subset of participants completing hyperinsulinaemic-euglycaemic clamp testing [102]. A prospective protocol document or trial registration was available only for three trials, thus making it difficult to judge whether all intended outcomes had been reported. The remaining 14 trials (78%) were judged to have an unclear risk of reporting bias, and one trial [108] was judged to be high risk due to incomplete reporting of results.

Eleven trials (61%) had low risk of bias based upon statistical similarities between groups at baseline [32, 34, 98–100, 102, 105, 108, 111, 114, 117]. Of the high-risk trials, one [119] had participants in the intervention group with less favourable adiposity and body composition versus control. Similarly, another trial [97] had an older exercise group that was less hyperandrogenic and hirsute, and had lower levels of CRF and higher BMI, plasma lipids, and IR levels compared with controls.

Adherence was reported in seven trials (39%) [32, 34, 97, 111, 117–119], with a median of 90% adherence, ranging from 67% [34] to 103% [111]. Two of the trials (11%) reported intervention adherence below the 75% threshold outlined in the “Methods” section [34, 119]. Five trials (28%) were judged to have a low risk of adherence bias (adherence ≥ 75%) [32, 97, 111, 117, 118]. Finally, most trials (*n* = 14, 78%) were deemed to have unclear risk of contamination bias due to lack of reporting. Only one trial [118] had a low risk of contamination bias as the control group did not increase PA > 4 MET/h/week [123], whereas three trials (17%) had a high risk of contamination as it was reported that comparison groups had either engaged in treatment [34, 111] or control groups had not received their allocated intervention [108].

### Effects of interventions: Exercise versus control

Due to data availability, a meta-analysis was possible only for three comparisons: (1) exercise versus control, (2) exercise and diet combined versus control, and (3) exercise and diet combined versus diet only.

Eleven trials were included in the exercise versus control meta-analysis as presented in Table 3 [32, 34, 102, 104, 108–111, 117–119].

**Table 3:** Effect estimates and heterogeneity for change from baseline to post-intervention scores and immediately post-intervention values, for all outcomes analysed in the exercise versus control comparison

Outcome	References	Change from baseline					Immediately post-intervention values				
		*N*	MD	Lower 95% CI	Upper 95% CI	*I*^2^ (%)	*N*	MD	Lower 95% CI	Upper 95% CI	*I*^2^ (%)
SBP (mmHg)	[101, 108, 117, 118]	158	− 2.93	− 7.06	1.20	50	158	2.02	− 6.82	10.86	87
DBP (mmHg)	[101, 108, 117, 118]	158	− 2.19	− 5.23	0.85	46	158	− 0.82	− 3.49	1.84	31
FBG (mg/dL)	[32, 100, 101, 107, 109, 110, 117–119]	263	− 1.08	− 2.47	0.30	16	238	− 1.69	− 4.35	0.97	37
FI (μIU/mL)	[32, 100, 101, 107, 109, 110, 117–119]	263	− 2.44**	− 4.24	− 0.64	91	238	− 2.11**	− 3.49	− 0.73	40
HOMA-IR	[32, 100, 101, 107, 109, 110, 117, 119]	173	− 0.57**	− 0.99	− 0.14	87	148	− 0.22	− 0.80	0.36	69
TC (mg/dL)	[32, 101, 108–110, 117, 118]	225	− 5.88**	− 9.92	− 1.83	35	225	− 6.35**	− 10.76	− 1.95	0
LDL-C (mg/dL)	[32, 101, 108–110, 117, 118]	225	− 7.39***	− 9.83	− 4.95	0	225	− 6.68**	− 11.66	− 1.70	0
HDL-C (mg/dL)^▲^	[32, 101, 108–110, 117, 118]	225	0.29	− 1.46	2.04	52	225	1.87	− 1.59	5.33	65
TG (mg/dL)	[32, 101, 108–110, 117, 118]	225	− 4.78***	− 7.52	− 2.05	3	225	− 1.97	− 7.36	3.42	18
VO_2_ max (ml/kg/min)^▲^	[32, 100, 103, 107, 109, 118]	229	3.84***	2.87	4.81	17	184	5.01***	3.48	6.54	42
RHR (bpm)	[32, 101, 117, 118]	156	− 2.65	− 5.55	0.25	51	156	− 3.26***	− 4.93	− 1.59	0
BMI (kg/m^2^)	[32, 34, 100, 101, 103, 107, 109, 110, 117, 118, 119]	331	− 0.49	− 1.04	0.06	66	272	− 1.02**	− 1.81	− 0.23	0
Body Mass (kg)	[32, 34, 101, 103, 109, 110, 119]	139	− 1.25	− 3.27	0.76	33	128	− 0.48	− 4.86	3.91	0
WC (cm)	[32, 34, 108, 109, 117–119]	221	− 2.62***	− 4.13	− 1.11	53	221	− 2.33	− 5.23	0.58	15
WHR	[101, 118]	101	− 0.03	− 0.08	0.02	0	101	− 0.04	− 0.08	0.01	19
Body Fat (%)	[32, 109, 119]	60	− 1.39*	− 2.61	− 0.18	30	60	− 3.28	− 7.39	0.83	22
Fat Mass (kg)	[32, 101, 119]	63	− 1.70	− 3.93	0.53	70	38	5.14	− 14.39	24.68	65
FFM (kg)^▲^	[32, 101, 119]	63	0.46	− 0.89	1.81	58	38	4.99	− 7.31	17.28	75
Testosterone (nmol/L)	[32, 101, 117–119]	203	− 0.09	− 0.24	0.06	0	169	− 0.08	− 0.35	0.19	37
SHBG (nmol/L)	[32, 101, 118, 119]	173	7.51	− 8.01	23.04	89	139	4.03	− 18.57	26.63	66
Free T (pg/mL)	[101, 117]	74	− 0.43	− 1.74	0.88	76	41	0.33	− 0.10	0.77	0
FAI	[32, 101, 118, 119]	139	0.24	− 0.55	1.04	0	139	0.68	− 1.09	2.44	46
FG	[101, 118]	135	− 0.63	− 2.08	0.81	0	101	− 0.75	− 2.03	0.54	0
Oestradiol (pmol/L)	[100, 101, 117, 118]	190	− 13.94	− 54.53	26.64	65	120	0.27	− 11.27	11.80	0
DHEA-S (μmol/L)	[32, 101]	70	− 0.60	− 1.58	0.39	0	36	− 0.20	− 1.87	1.46	0
LH (IU/L)	[101, 104, 117, 118]	185	− 0.30	− 2.54	1.95	72	151	− 0.66	− 2.39	1.06	43
FSH (IU/L)	[101, 104, 117, 118]	185	0.23	− 0.08	0.53	0	151	− 0.01	− 0.40	0.37	0
LH/FSH ratio	[101, 117]	41	− 0.02	− 0.38	0.33	0	41	0.32	− 0.22	0.86	37
PG (nmol/L)	[102, 118]	115	− 0.72	− 2.53	1.09	74	–	–	–	–	–
Prolactin (ng/mL)	[104, 118]	110	− 0.05	− 0.71	0.61	0	110	0.20	− 0.27	0.68	0
hsCRP (mg/L)	[32, 119]	38	− 0.41	− 1.19	0.37	0	38	0.67	− 1.31	2.65	0
AMH (ng/mL)	[32, 109, 110]	67	− 0.67	− 1.65	0.32	0	67	0.48	− 1.89	2.84	0
Adiponectin (μg/mL)	[32, 101]	70	− 0.20	− 1.04	0.64	0	–	–	–	–	–

Effect estimates are reported as mean differences (MD) and 95% confidence intervals, between exercise and usual care groups. Heterogeneity reported using *I*^2^ statistic

Key: *95% CI* 95% confidence intervals, *SBP* systolic blood pressure, *DBP* diastolic blood pressure, *FBG* fasting blood glucose, *FI* fasting insulin, *HOMA-IR* homeostatic model of assessment - insulin resistance, *TC* total cholesterol, *LDL-C* low-density lipoprotein cholesterol, *HDL-C* high-density lipoprotein cholesterol, *TG* triglycerides, *RHR* resting heart rate, *BMI* body mass index, *WC* waist circumference, *WHR* waist-to-hip ratio, *FFM* fat-free mass, *SHBG* sex hormone binding globulin, *Free T* free testosterone, *FAI* free androgen index, *FG* Ferriman-Gallwey score, *DHEA-S* dehydroepiandrosterone sulfate, *LH* luteinising hormone, *FSH* follicle stimulating hormone, *PG* progesterone, *hsCRP* high-sensitivity C-reactive protein, *AMH* anti-Müllerian hormone. *N* number or participants included within analysis

^▲^Positive values favour exercise over control.

^•^Study only included in the change from baseline analysis

Statistically significant effects denoted by *P ≤ 0.05; **P ≤ 0.01; ***P ≤ 0.001

### Primary outcomes

#### Blood pressure

Four eligible trials (158 participants) assessed changes in blood pressure. We found no significant effect of exercise on systolic blood pressure (SBP) or diastolic blood pressure (DBP) for either change scores or post-intervention values compared with control (Table 3). We rated the result of both SBP and DBP as low-quality evidence due to imprecision (small number of participants, and a null and appreciable effect were included in the 95% CI for the MD), and high or unclear risk of selection bias, detection bias, reporting bias, attrition bias, and contamination (see Table 4; Summary of findings for primary outcomes).

**Table 4:** Summary of findings for primary outcomes: exercise versus control

Exercise compared to usual care for women with PCOS						
Patient or population: women with PCOS Setting: Intervention: exercise Comparison: usual care						
Outcomes	Anticipated absolute effects* (95% CI)		Relative effect(95% CI)	№ of participants (studies)	Certainty of the evidence(GRADE)	Comments
	Risk with usual care	Risk with exercise				
Systolic blood pressure (change from baseline) follow-up: range 8 weeks to 16 weeks	The mean systolic blood pressure (change from baseline) ranged from − 2.5 to 1.1 mmHg	The mean systolic blood pressure (change from baseline) in the intervention group was 2.93 mmHg lower (7.06 lower to 1.2 higher)	–	158 (4 RCTs)	⨁⨁◯◯ LOW ^a,b^	Exercise may result in little to no difference in systolic blood pressure (change from baseline).
Diastolic blood pressure (change from baseline)follow-up: range 8 weeks to 16 weeks	The mean diastolic blood pressure (change from baseline) ranged from −3.1 to 2.9 mmHg	The mean diastolic blood pressure (change from baseline) in the intervention group was 2.19 mmHg lower (5.23 lower to 0.85 higher)	–	158 (4 RCTs)	⨁⨁◯◯ LOW ^a,b^	Exercise may result in little to no difference in diastolic blood pressure (change from baseline).
Fasting blood glucose (change from baseline)follow-up: range 8 weeks to 16 weeks	The mean fasting blood glucose (change from baseline) ranged from − 1.3 to 2.6 mg/dL	The mean fasting blood glucose (change from baseline) in the intervention group was 1.08 mg/dL lower (2.47 lower to 0.3 higher)	–	263 (9 RCTs)	⨁⨁◯◯ LOW ^c,d^	Exercise may result in little to no difference in fasting blood glucose (change from baseline).
Fasting insulin (change from baseline) follow-up: range 8 weeks to 16 weeks	The mean fasting insulin (change from baseline) ranged from −4.1 to 2.5 μU/ml	The mean fasting insulin (change from baseline) in the intervention group was 2.44 μU/ml lower (4.42 lower to 0.64 lower)	–	263 (9 RCTs)	⨁◯◯◯ VERY LOW ^e,f,g^	Exercise may reduce fasting insulin (change from baseline) but we are very uncertain.
HOMA-IR (change from baseline)follow-up: range 8 weeks to 16 weeks	The mean HOMA-IR (change from baseline) ranged from − 0.4 to 0.7	The mean HOMA-IR (change from baseline) in the intervention group was 0.57 lower (0.99 lower to 0.14 lower)	–	173 (8 RCTs)	⨁◯◯◯ VERY LOW ^d,e,h^	Exercise may reduce HOMA-IR (change from baseline) but we are very uncertain.
Total cholesterol (change from baseline) follow-up: range 8 weeks to 16 weeks	The mean total cholesterol (change from baseline) ranged from −8.85 to 6.85 mg/dL	The mean total cholesterol (change from baseline) in the intervention group was 6.48 mg/dL lower (10.5 lower to 2.45 lower)	–	225 (7 RCTs)	⨁⨁◯◯ LOW ^g,i^	Exercise may reduce total cholesterol (change from baseline) slightly.
LDL-C (change from baseline) follow-up: range 8 weeks to 16 weeks	The mean LDL-C (change from baseline) ranged from − 17.7 to 7.03 mg/dL	The mean LDL-C (change from baseline) in the intervention group was 7.51 mg/dL lower (10.01 lower to 5.02 lower)	–	225 (7 RCTs)	⨁⨁◯◯ LOW ^g,i^	Exercise may reduce LDL-C (change from baseline) slightly.
HDL-C (change from baseline) follow-up: range 8 weeks to 16 weeks	The mean HDL-C (change from baseline) ranged from − 17.7 to 3.5 mg/dL	The mean HDL-C (change from baseline) in the intervention group was 0.01 mg/dL lower (1.91 lower to 1.89 higher)	–	225 (7 RCTs)	⨁⨁◯◯ LOW ^d,i^	Exercise may result in little to no difference in HDL-C (change from baseline).
Triglycerides (change from baseline) follow-up: range 8 weeks to 16 weeks	The mean triglycerides (change from baseline) ranged from − 1.0 to 8.9 mg/dL	The mean triglycerides (change from baseline) in the intervention group was 4.78 mg/dL lower (7.52 lower to 2.05 lower)	–	225 (7 RCTs)	⨁⨁◯◯ LOW ^g,i^	Exercise likely results in a small effect that may not be an important (or unimportant) reduction in triglycerides (change from baseline).

*The risk in the intervention group (and its 95% confidence interval) is based on the assumed risk in the comparison group and the relative effect of the intervention (and its 95% CI)

*CI* confidence interval, *MD* mean difference

GRADE Working Group grades of evidence

High certainty: We are very confident that the true effect lies close to that of the estimate of the effect

Moderate certainty: We are moderately confident in the effect estimate: The true effect is likely to be close to the estimate of the effect, but there is a possibility that it is substantially different

Low certainty: Our confidence in the effect estimate is limited: The true effect may be substantially different from the estimate of the effect

Very low certainty: We have very little confidence in the effect estimate: The true effect is likely to be substantially different from the estimate of effect

Explanations

^a^Three of the four trials had a high or unclear risk of selection bias, detection bias, and reporting bias; all were at high risk of performance bias; two were at high or unclear risk of attrition bias; and all were at a high or unclear risk of contamination. Therefore we downgraded by one level

^b^Small number of participants, wide confidence intervals for three of the four trials, and null/negligible effect and appreciable benefit included in the confidence interval for the mean difference. Therefore, we downgraded by one level

^c^Most trials were at an unclear or high risk of selection bias, detection bias, and reporting bias; and all trials were at a high or unclear risk of contamination and low adherence. Therefore, we downgraded by one level

^d^Small number of participants and null/negligible effect and appreciable benefit included in the confidence interval for the mean difference. Therefore, we downgraded by one level

^e^Most trials were at an unclear or high risk of selection bias, detection bias, attrition bias, and reporting bias; and most trials were at a high or unclear risk of contamination and low adherence. Therefore, we downgraded by one level

^f^Considerable heterogeneity was observed. Therefore, we downgraded by one level

^g^Small number of participants and wide confidence intervals in the included trials. Therefore, we downgraded by one level

^h^Considerable heterogeneity was observed and there was minimal or no overlap of confidence intervals. Therefore, we downgraded by one level

^i^Most trials were at an unclear or high risk of selection bias, detection bias, and reporting bias; and all trials were at a high or unclear risk of contamination. Therefore, we downgraded by one level

In subgroup analyses (Additional file 1: Table S5), we only found effects of supervised interventions (MD: − 4.42 mmHg, 95% CI: -8.32 to − 0.51; 3 trials, 147 participants, *I*^2^ = 31%) on the SBP change compared with control. No effects were found in the subgroup analysis of SBP post-intervention values or in any DBP subgroup analysis.

#### Fasting blood glucose

Based on data from nine trials (263 participants), we found no effect of exercise on fasting blood glucose (FBG) change or absolute post-intervention values compared with control (Table 3). There was also no effect of exercise for any of the subgroup analyses presented in Additional file 1: Table S5. We rated the result as low-quality evidence due to an unclear or high-risk of selection, detection, and reporting bias, contamination, low adherence, small number of participants, and a null or negligible effect and appreciable benefit included in the confidence interval for the mean difference (Table 4).

#### Fasting insulin

**Fig. 3:**
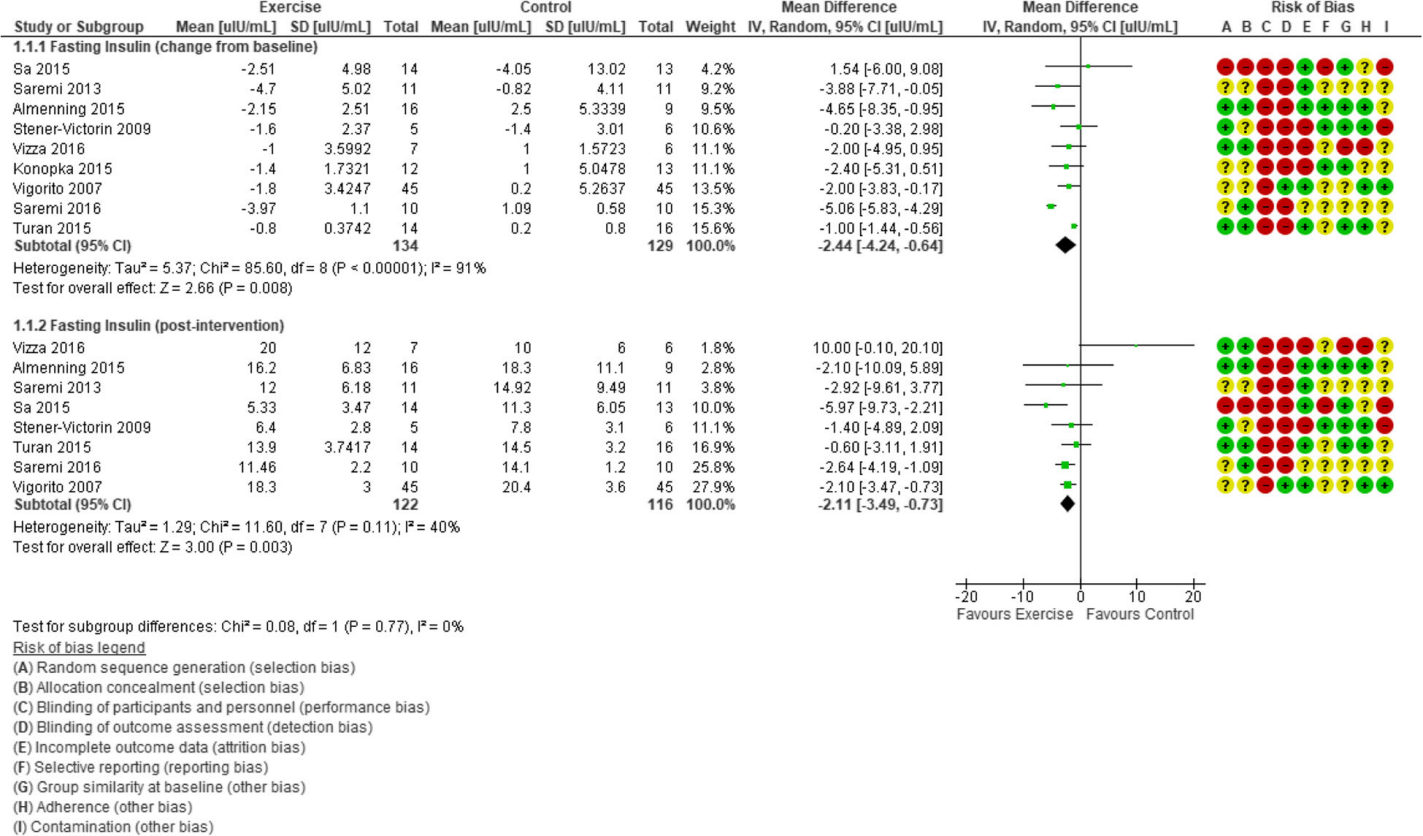
Forest plot of comparison: exercise vs. control, outcome: fasting insulin plasma levels (μIU/mL)

Meta-analysis of nine trials (263 participants) revealed a favourable effect of exercise on the change of FI values from baseline compared with control (MD − 2.44 μIU/mL, 95% CI − 4.24 to − 0.64; Fig. 3), but with evidence of considerable heterogeneity (*I*^2^ = 91%). Similarly, statistically significant lowering effects of exercise versus control were found for FI post-intervention values (MD − 2.11 μIU/mL, 95% CI − 3.49 to − 0.73; 8 trials, 238 participants, *I*^2^ = 40%). Applying GRADE, we rated the result as very low-quality (Table 4) evidence due to unclear or high-risk randomisation or allocation procedures, lack of blinding, high rate of incomplete outcome data, unclear reporting of outcomes and contamination, low adherence, considerable heterogeneity in the effects in individual studies, small number of participants, and wide confidence interval for the mean difference.

In sensitivity analyses, the observed effect of exercise on FI change from baseline remained when only trials with larger sample sizes (*n* ≥ 30 total participants) (MD − 1.09 μIU/mL, 95% CI − 1.64 to − 0.53; 2 trials, 120 participants, *I*^2^ = 7%) and studies with a low risk of bias (MD − 3.18 μIU/mL, 95% CI − 5.63 to − 0.74; 187 participants, 5 trials, *I*^2^ = 95%) were included. Likewise, post-intervention FI effects remained when small trials (MD − 1.73 μIU/mL, 95% CI − 3.00 to − 0.47; 2 trials, 160 participants, *I*^2^ = 5%) and trials with a high risk of bias (MD − 2.10 μIU/mL, 95% CI − 3.04 to − 1.17; 5 trials, 187 participants, *I*^2^ = 0%) were removed.

To identify the potential source of heterogeneity in the FI change analysis, when the greatest outlier [110] was removed, the *I*^2^ statistic was reduced to a level that may not be important (18%) and the effect was maintained (MD − 1.54 μIU/mL, 95% CI − 2.36 to − 0.71). The results of the removed trial may have varied due to the mode of exercise used (resistance training) or the use of a placebo.

A statistical effect of exercise versus control on FI was shown in multiple subgroups (Additional file 1: Table S5). We found a change in FI from baseline to post-intervention in studies with participants who were overweight (BMI 25–29.9 kg/m^2^, MD − 3.25 μIU/mL, 95% CI − 5.27 to − 1.22; 5 trials, 168 participants, *I*^2^ = 75%); interventions that were aerobic exercise-based (MD − 2.22 μIU/mL, 95% CI − 3.57 to − 0.86; 6 trials, 192 participants, *I*^2^ = 10%); ≤ 12 weeks duration (MD − 2.92 μIU/mL, 95% CI − 4.91 to − 0.93; 7 trials, 225 participants, *I*^2^ = 93%); and supervised and combined supervised and unsupervised (MD − 2.54 μIU/mL, 95% CI − 4.82 to − 0.26; 6 trials, 214 participants, *I*^2^ = 94%, and MD − 3.08 μIU/mL, 95% CI − 5.63 to − 0.53; 2 trials, 38 participants, *I*^2^ = 17%, respectively).

Compared with control, favourable effects of exercise on FI post-intervention values were found for participants who were overweight (MD − 2.27 μIU/mL, 95% CI − 3.24 to − 1.31; 5 trials, 168 participants, *I*^2^ = 0%); interventions that were aerobic exercise-based (MD − 2.48 μIU/mL, 95% CI − 3.92 to − 1.04; 5 trials, 167 participants, *I*^2^ = 10%); ≤ 12 weeks duration (MD − 1.80 μIU/mL, 95% CI − 3.18 to − 0.42; 6 trials, 200 participants, *I*^2^ = 32%); and supervised (MD − 2.39 μIU/mL, 95% CI − 3.62 to − 1.17; 5 trials, 189 participants, *I*^2^ = 30%).

#### HOMA-IR

**Fig. 4:**
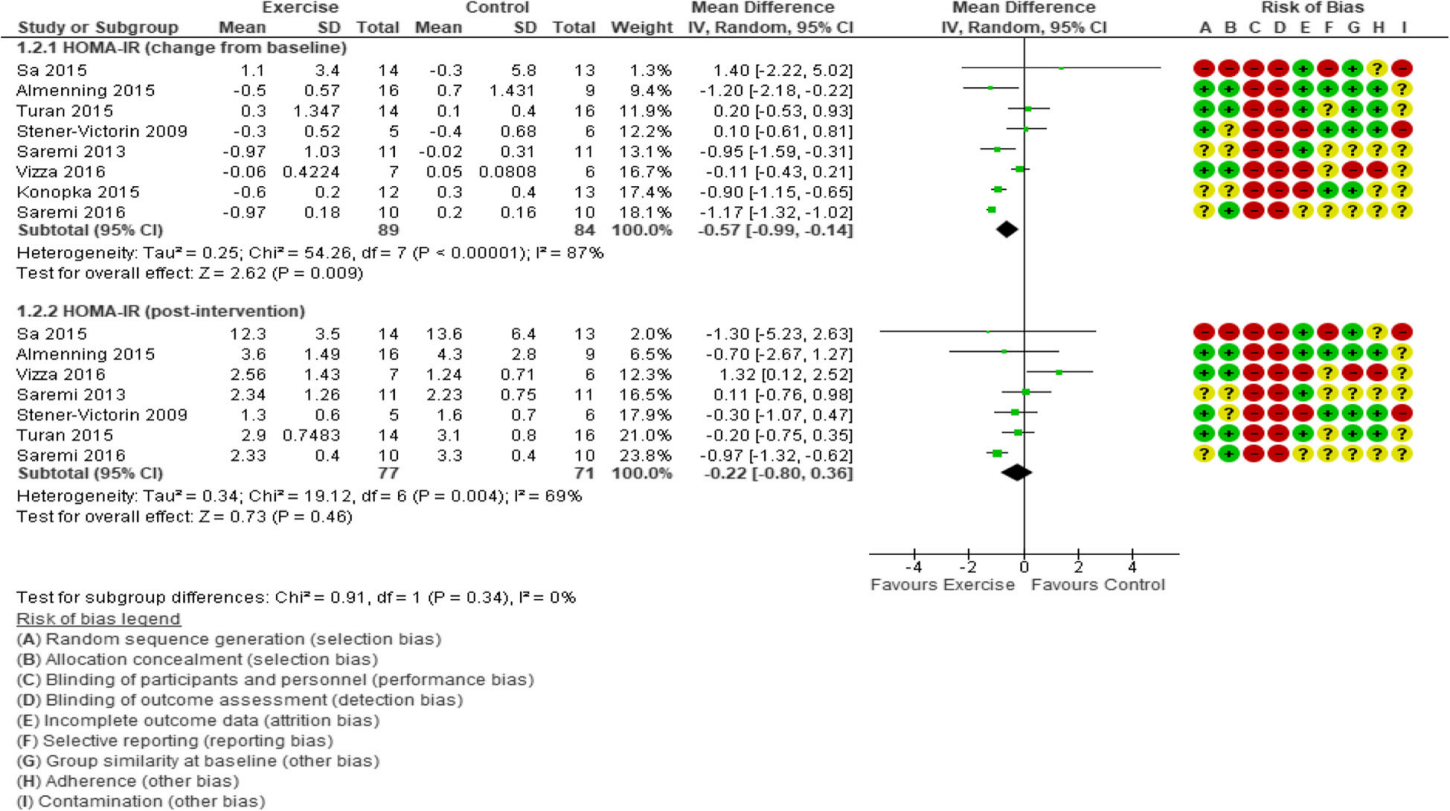
Forest plot of comparison: exercise vs. control, outcome: Homeostatic Model Assessment for Insulin Resistance (HOMA-IR)

Greater reductions in HOMA-IR change scores were evident for exercise versus control (MD − 0.57, 95% CI − 0.99 to − 0.14; 8 trials, 173 participants, *I*^2^ = 87%; Table 3; Fig. 4), but the comparison of post-intervention HOMA-IR values did not reveal a significant exercise effect. In a sensitivity analysis including only trials at a low risk of bias, the effect of exercise was maintained (MD − 0.81, 95% CI − 1.40 to − 0.21; 97 participants, 4 trials, *I*^2^ = 77%) for HOMA-IR changes. Only one trial had a sample size of ≥ 30 participants [117], so a corresponding sensitivity analysis was not possible. We rated the result as very low-quality evidence due to unclear or high risk of selection, detection, attrition, and reporting bias, contamination, low adherence, considerable heterogeneity with minimal or no overlap of confidence intervals, small number of participants, and a null or negligible effect and appreciable benefit included in the confidence interval for the mean difference (Table 4).

In the investigation of heterogeneity, removing the most extreme value [32] had a negligible effect on the *I*^2^ (89%), but a small effect was maintained (MD − 0.50, 95% CI − 0.96 to − 0.05). Similarly, the *I*^2^ statistic was still representative of at least substantial heterogeneity in sub-analyses. The lowest reported value (*I*^2^ = 60%) was in the aerobic exercise intervention subgroup.

Subgroup analyses revealed statistical effects on HOMA-IR change from baseline for aerobic exercise interventions (MD − 0.73, 95% CI − 1.24 to − 0.21; 5 trials, 102 participants, *I*^2^ = 60%); ≤ 12 weeks duration (MD − 0.69, 95% CI − 1.13 to − 0.26; 6 trials, 135 participants, *I*^2^ = 89%); and supervised delivery (MD − 0.80, 95% CI − 1.19 to − 0.42; 5 trials, 124 participants, *I*^2^ = 76%); and for participants in the overweight subgroup (MD − 0.83, 95% CI − 1.39 to − 0.26; 4 trials, 78 participants, *I*^2^ = 75%). Post-intervention subgroup analysis revealed no effects (Additional file 1: Table S5).

#### Circulating lipids

**Fig. 5:**
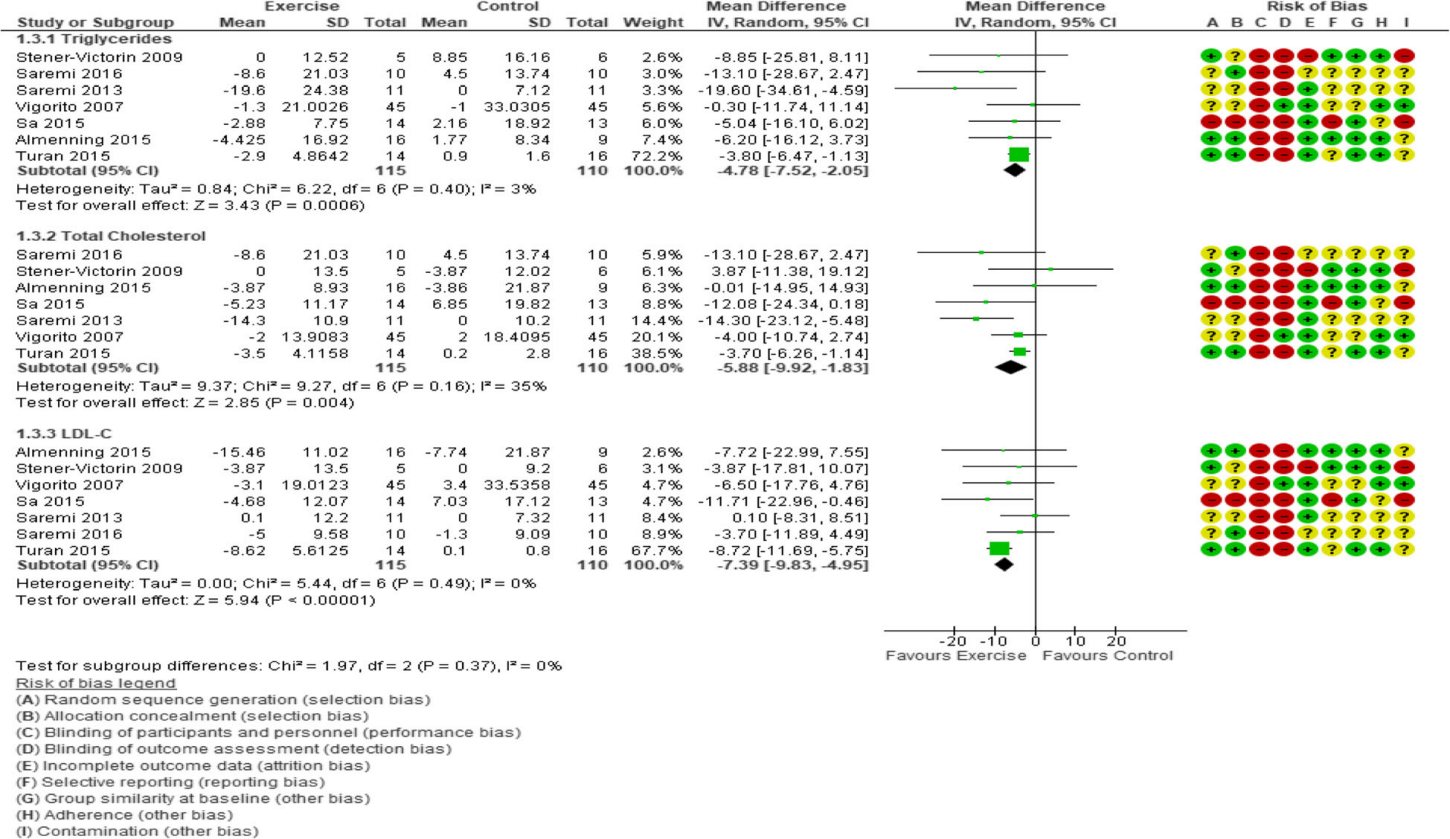
Forest plot of comparison: exercise vs. control, change from baseline to immediately post-intervention analysis of outcomes related to lipid profile (mg/dL)

Seven trials (225 participants) were included in the analysis of all lipid-related outcomes (TC, LDL-C, and HDL-C, and triglycerides; Fig. 5; Table 3). A statistically significant effect of exercise versus control was observed for TC change scores (MD − 5.88 mg/dL, 95% CI − 9.92 to − 1.83; *I*^2^ = 35%), LDL-C (MD − 7.39 mg/dL, 95% CI − 9.83 to − 4.95; *I*^2^ = 0%), and triglycerides (MD − 4.78 mg/dL, 95% CI − 7.52 to − 2.05; *I*^2^ = 3%), but not for HDL-C (Table 3). Post-intervention values analysis of lipid-related outcomes revealed an effect on TC (MD − 6.35 mg/dL, 95% CI − 10.76 to − 1.95; *I*^2^ = 0%) and LDL-C (MD − 6.68 mg/dL, 95% CI − 11.66 to − 1.70; *I*^2^ = 0%) (Table 3). We rated these results as low-quality evidence (Table 4) due to high or unclear risk of selection bias, detection bias, reporting bias, contamination, and imprecision due to small number of participants and wide confidence intervals in the included trials.

In sensitivity analyses, the favourable effects of exercise versus control on TC, LDL-C, and triglycerides change scores were maintained in studies with a low risk of bias (MD − 5.94 md/dL, 95% CI − 10.32 to − 1.55; 5 trials, 187 participants, *I*^2^ = 40%; MD − 6.60 mg/dL, 95% CI − 9.88 to − 3.32; 5 trials, 187 participants, *I*^2^ = 14%; and MD − 5.97 mg/dL, 95% CI − 10.91 to − 1.03; 5 trials, 187 participants, *I*^2^ = 33%, respectively), and in larger trials (MD − 3.74 mg/dL, 95% CI − 6.13 to − 1.35; 120 participants, 2 trials, *I*^2^ = 0%; MD − 8.58, 95% CI − 11.44 to − 5.71; 120 participants, 2 trials, *I*^2^ = 0%; and MD − 3.62 mg/dL, 95% CI − 6.22 to − 1.02; 120 participants, 2 trials, *I*^2^ = 0%, respectively). Sensitivity analyses for LDL-C post-intervention values showed a retained effect when trials with a high risk of bias were excluded (MD − 8.64 mg/dL, 95% CI − 16.30 to − 0.98; 5 trials, 187 participants, *I*^2^ = 22%), but not when smaller trials were removed.

Subgroup analyses of TC change (Additional file 1: Table S6) revealed statistical effects for interventions that were ≤ 12 weeks duration (MD − 5.94 mg/dL, 95% CI − 10.32 to − 1.55; 5 trials, 187 participants, *I*^2^ = 37%) or supervised (MD − 7.25 mg/dL, 95% CI − 11.92 to − 2.58; 5 trials, 189 participants, *I*^2^ = 48%). There was also an effect in subgroup analysis for change from baseline (MD − 6.68 mg/dL, 95% CI − 13.00 to − 0.35; 5 trials, 167 participants, *I*^2^ = 39%) and post-intervention TC values (MD − 6.90 mg/dL, 95% CI − 11.90 to − 1.90; 5 trials, 167 participants, *I*^2^ = 0%) in aerobic exercise interventions. Subgroup analysis of post-intervention TC also revealed an effect when interventions > 12 weeks (MD − 9.92 mg/dL, 95% CI − 17.81 to − 2.04; 2 trials, 38 participants, *I*^2^ = 0%) or were supervised (MD − 6.76 mg/dL, 95% CI − 11.27 to − 2.26; 5 trials, 189 participants, *I*^2^ = 0%).

In subgroup analyses for LDL-C change from baseline, a statistically favourable exercise effect was found in trials consisting of interventions ≤ 12 weeks duration (MD − 6.60 mg/dL, 95% CI − 9.88 to − 3.32; 5 trials, 187 participants, *I*^2^ = 13%) or supervised (MD − 6.70 mg/dL, 95% CI − 10.29 to − 3.12; 5 trials, 189 participants, *I*^2^ = 23%). Subgroup analysis for LDL-C post-intervention values revealed statistical effects in participants with BMI of 25–29.9 kg/m^2^ (MD − 9.54 mg/dL, 95% CI − 18.71 to − 0.36; 5 trials, 168 participants, *I*^2^ = 22%), and interventions of ≤ 12 weeks duration (MD − 8.64 mg/dL, 95% CI − 16.30 to − 0.98; 5 trials, 187 participants, *I*^2^ = 22%), supervised (MD − 7.58 mg/dL, 95% CI − 13.73 to − 1.43; 5 trials, 187 participants, *I*^2^ = 24%), or aerobic (MD: − 5.87 mg/dL, 95% CI − 11.68 to − 0.07; 5 trials, 167 participants, *I*^2^ = 0%; Additional file 1: Table S6).

For HDL-C, only subgroup analyses of resistance training interventions showed a negative effect on change from baseline scores (MD − 2.19 mg/dL, 95% CI − 4.21 to − 0.18; 2 trials, 37 participants, *I*^2^ = 0%) and a positive effect on post-intervention values (MD 7.29 mg/dL, 95% CI 1.11 to 13.46; 2 trials, 37 participants, *I*^2^ = 17%; Additional file 1: Table S6). No effects of exercise were found in other HDL-C subgroup analyses.

Compared with control, exercise had a favourable effect on triglyceride values in the following subgroups: BMI 25–29.9 kg/m^2^ (MD − 8.17 mg/dL, 95% CI − 14.44 to − 1.89; 5 trials, 167 participants, *I*^2^ = 13%); aerobic exercise interventions (MD − 6.80 mg/dL, 95% CI − 13.12 to − 0.48; 5 trials, 167 participants, *I*^2^ = 5%); ≤ 12 weeks duration (MD − 6.06 mg/dL, 95% CI − 10.82 to − 1.31; 5 trials, 187 participants, *I*^2^ = 30%); and supervised interventions (MD − 5.91 mg/dL, 95% CI − 10.75 to − 1.06; 5 trials, 189 participants, *I*^2^ = 29%; Additional file 1: Table S6). Analysis of triglyceride post-intervention values revealed an effect of exercise in trials > 12 weeks only (MD − 13.85 mg/dL, 95% CI − 26.33 to − 1.36; 2 trials, 38 participants, *I*^2^ = 0%).

### Secondary outcomes

#### Maximal or peak oxygen uptake

**Fig. 6:**
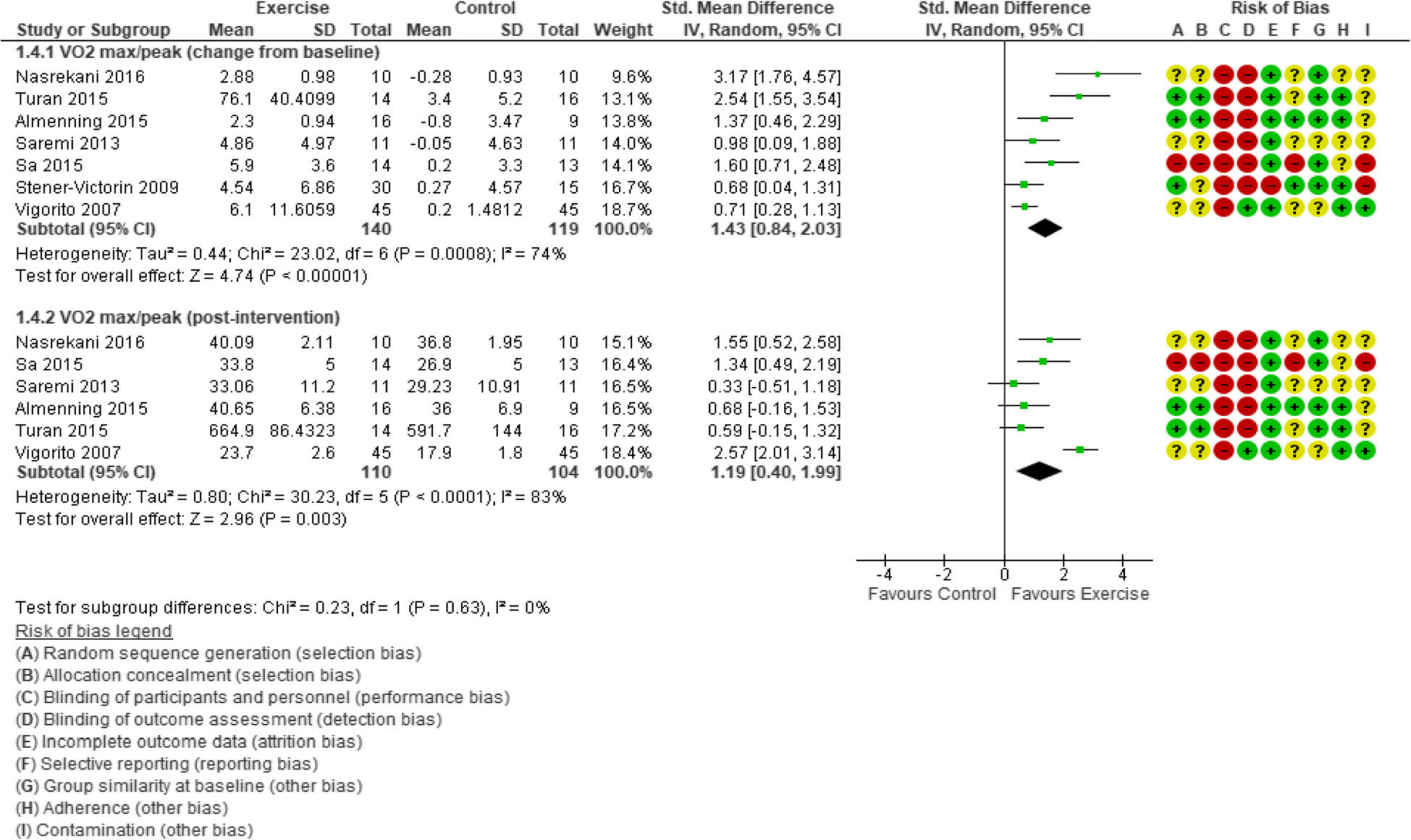
Forest plot of comparison: exercise vs. control, standardised mean difference; outcome: VO_2_ max/peak

A large statistical effect of exercise versus control was found for both change from baseline and post-intervention VO_2_ max/peak values (SMD 1.43, 95% CI 0.84 to 2.03; 259 participants, 7 trials, *I*^2^ = 74%, and SMD 1.19, 95% CI 0.40 to 1.99; *I*^2^ = 83%, respectively; Fig. 6). With the inclusion of only studies that reported relative VO_2_ max/peak values (i.e., expressed as ml/kg/min), the effect of exercise was maintained in both change scores and post-intervention values (MD 3.84 ml/kg/min, 95% CI 2.87 to 4.81; 6 trials, 229 participants, *I*^2^ = 17%, and MD 5.01 ml/kg/min, 95% CI 3.48 to 6.54; 5 trials, 184 participants, *I*^2^ = 42%, respectively).

For the SMD VO_2_ max/peak change sensitivity analysis, an effect remained when small trials (SMD 1.21, 95% CI 0.29 to 2.12; 3 trials, 165 participants, *I*^2^ = 83%) and those with a high risk of bias (SMD 1.63, 95% CI 0.78 to 2.48; 5 trials, 187 participants, *I*^2^ = 80%) were removed. SMD was also used to complete post-intervention sensitivity analysis for sample size; 2 trials (120 participants) [116, 117] were included, but the effect was lost. However, it remained when trials with a high risk of bias were removed (SMD 1.16, 95% CI 0.21 to 2.12; 5 trials, 187 participants, *I*^2^ = 87%).

When we considered only relative VO_2_ max/peak change scores, the effect of exercise was maintained when removing small studies (MD 1.21 ml/kg/min, 95% CI 0.29 to 2.12, 165 participants, 3 trials, *I*^2^ = 83%) and studies with a high risk of bias (MD 3.35 ml/kg/min, 95% CI 2.59 to 4.10; 157 participants, 4 trials, *I*^2^ = 0%). All trials in the post-intervention relative VO_2_ max/peak analysis were considered low risk of bias, so this sensitivity analysis was not possible.

For ease of interpretation, we performed subgroup analyses on the relative VO_2_ max/peak data. Subgroup analysis of the change from baseline relative VO_2_ max/peak values revealed statistical improvements with aerobic exercise, any intervention duration, and for participants with a BMI of 25–29.9 kg/m^2^. The post-intervention pooled analysis showed an effect of exercise on relative VO_2_ max/peak in four subgroups: participants with a BMI of 25–29.9 kg/m^2^, aerobic exercise interventions, ≤ 12 weeks, and supervised (Additional file 1: Table S7).

In one trial reporting data from a post-intervention 16-week follow-up [111], a 12% increase in VO_2_ max (4.11 ± 5.20 ml/kg/min; *p* = .001) from baseline was still evident in the exercise group. The corresponding change for control (7%) was not statistically significant, and there were no significant differences between groups.

#### Resting heart rate

A pooled analysis of four trials (156 participants) indicated no effect of exercise on the change scores of resting heart rate (RHR) values (Table 3). However, in these trials, RHR post-intervention values were statistically lower in the exercise interventions versus control (MD − 3.26 beats/min, 95% CI − 4.93 to − 1.59; *I*^2^ = 0%). When post-intervention sensitivity analyses were completed, this effect was still evident in larger trials (MD − 3.18 beats/min, 95% CI − 5.59 to − 0.77, 3 trials, 145 participants, *I*^2^ = 16%) and those with a low risk of bias (MD − 3.53 beats/min, 95% CI − 5.28 to − 1.78; 2 trials, 120 participants, *I*^2^ = 0%).

In subgroup analyses, there were statistical effects of exercise compared with control on both RHR change from baseline and post-intervention values in interventions that were aerobic exercise-based (Additional file 2: Figure S3), and those that were supervised. Post-intervention subgroup analysis also revealed effects in interventions of ≤ 12 weeks and when participants had a BMI 25–29.9 kg/m^2^ at study entry (Additional file 1: Table S7).

#### Body mass and body mass index

We found a statistical effect of exercise on BMI post-intervention values (MD − 1.02 kg/m^2^, 95% CI − 1.81 to − 0.23; 10 trials, 272 participants, *I*^2^ = 0%) compared with control (Table 3). When trials with a high risk of bias were removed from the sensitivity analysis for BMI post-intervention values, an effect remained (MD − 0.95 kg/m^2^, 95% CI − 1.78 to − 0.12; 6 trials, 207 participants, *I*^2^ = 0%), but not when small trials were removed.

Subgroup analysis revealed a statistical reduction in BMI change scores with exercise in studies consisting of participants with BMI ≥ 30 kg/m^2^. Analysis of BMI change from baseline also revealed a statistical decrease with aerobic exercise interventions, but a statistical increase with resistance training interventions (Additional file 1: Table S7).

Post-intervention subgroup analysis revealed statistical reductions in BMI with interventions that were aerobic exercise-based, supervised, and ≤ 12 weeks, and also in participants with a BMI of 25–29.9 kg/m^2^ (Additional file 1: Table S7).

The meta-analysis revealed no effect of exercise versus control on body mass change from baseline or post-intervention values (Table 3). However, we found statistical effects of exercise versus control on change in body mass from baseline to post-intervention for studies involving participants with BMI ≥ 30 kg/m^2^. No other subgroup analyses indicated such an effect (Additional file 1: Table S7).

Follow-up reporting (16-weeks post-intervention) of BMI from one trial [111] showed no statistically significant within-group changes or between-group differences in either exercise or control arms. The authors [111] also reported similar findings immediately post-intervention.

#### Waist and hip circumference and waist-to-hip ratio

Analysis of waist circumference (WC) change scores, but not post-intervention values, revealed a statistically significant beneficial effect of exercise compared with controls (MD − 2.62 cm, 95% CI − 4.13 to − 1.11; 7 trials, 221 participants, *I*^2^ = 53%; Table 3). The favourable effect of exercise on WC change remained when trials with a low risk of bias (MD − 1.51 cm, 95% CI − 2.26 to − 0.76; 167 participants, 4 trials, *I*^2^ = 0%) and larger sample sizes (MD − 1.48 cm, 95% CI − 2.26 to − 0.71; 120 participants, 2 trials, *I*^2^ = 0%) were analysed separately. When the largest outlier [108] was removed from this analysis, the *I*^2^ was reduced to 0% and an effect remained (MD − 1.68 cm, 95% CI − 2.38 to − 0.99).

In subgroup analyses for WC change, exercise had a statistical effect in studies with participants with BMI of 25–29.9 kg/m^2^ and ≥ 30 kg/m^2^, ≤ 12 week’s duration, aerobic and resistance-based interventions, and supervised exercise (Additional file 1: Table S7). Subgroup analysis revealed that post-intervention WC values were statistically lower in exercise interventions with participants with BMI 25–29.9 kg/m^2^, aerobic exercise, and supervised exercise (Additional file 1: Table S7).

Data from two trials [111, 118] were pooled in the analysis of waist-to-hip ratio (WHR); there was no effect in either change from baseline or post-intervention values analyses.

#### Body composition

The pooled MD for body fat percentage change from baseline was statistically significant (MD − 1.39%, 95% CI − 2.61 to − 0.18; 3 trials, 60 participants, *I*^2^ = 30%), but not for post-intervention values (Table 3). When trials deemed to have a high risk of bias were removed, this statistical effect disappeared. Sensitivity analysis by study size could not be performed for the exercise effect on body fat percentage due to a lack of sufficiently large studies. Moreover, we found no effect of exercise versus control on change from baseline or post-intervention analyses for fat mass and fat-free mass (Table 3).

A statistical effect was found for exercise on body fat percentage change in interventions ≤ 12 weeks, but this analysis included the same trials as the main analysis. No other statistical effects were found across any of the other subgroup analyses on body fat percentage change (Additional file 1: Table S7). However, body fat percentage was statistically lower post-intervention in exercise interventions that included participants with BMI of 25–29.9 kg/m^2^, and aerobic exercise (Additional file 1: Table S7). No effect was evident in the subgroup analysis for fat mass or fat-free mass.

#### Androgenic, hormonal, and inflammatory markers

In pooled analyses of change from baseline or post-intervention values, exercise had no beneficial effect on any of the androgenic/hormonal and inflammatory biomarkers/variables [i.e., testosterone, free testosterone, free androgen index (FAI), sex hormone binding globulin (SHBG), Ferriman-Gallwey scores, oestradiol, luteinising hormone (LH), follicle stimulating hormone (FSH), LH/FSH ratio, progesterone, prolactin, high-sensitivity C-reactive protein, anti-Mullerian hormone (AMH), or adiponectin] when compared with control (Table 3). Similarly, there were no effects in any subgroup analysis for these outcomes (Additional file 1: Table S8).

#### Psychosocial outcomes

In two trials (57 participants) that assessed psychosocial outcomes using the PCOS-Q, we found no effect of exercise on any PCOS-Q domain compared with control. Three trials (84 participants) used the SF-36. Data only allowed for change from baseline analysis and no sub-analysis was possible. For SF-36 domains, a favourable effect of exercise versus control was found for physical functioning (MD 11.81, 95% CI 2.36 to 21.25; *I*^2^ = 74%), general health (MD 10.05, 95% CI 3.89 to 16.20; *I*^2^ = 0%), social functioning (MD 11.75, 95% CI 2.56 to 20.95; *I*^2^ = 6%), and mental health (MD 11.70, 95% CI 1.27 to 22.13; *I*^2^ = 47%) domains (Additional file 2: Figure S5).

There were insufficient data to complete sensitivity analyses; however, all three trials [108, 111, 119] were judged to have a high risk of bias in at least one domain, and only one trial had a sample size ≥ 30. Heterogeneity was investigated in the physical functioning domain; the largest outlier was removed [108] and the *I*^2^ was reduced to 33%, whilst an effect was maintained (MD 7.23, 95% CI 1.66 to 12.80). The same trial was removed in the general health analysis, resulting in a reduction in *I*^2^ to 0%, and a preserved effect (MD 7.97, 95% CI 1.07 to 4.88). When the greatest outliers were removed from the social functioning [119] and mental health [111] domains, both *I*^2^ values were reduced to 0%, but the effect only remained in the mental health domain (MD 17.84, 95% CI 7.33 to 28.36).

#### Additional outcomes

Six trials [32, 108, 111, 117–119] also reported a range of additional outcomes; the key findings from these are presented in Additional file 1: Table S9.

### Effects of interventions: Exercise and diet versus control

Three trials compared exercise and diet combined versus control. Only one of these trials used a control group that was described as no treatment [99]. The other two [100, 107] compared exercise, diet and metformin (or placebo) to metformin only groups. As pharmacological intervention was present in each included treatment arm, we assumed that any variation between groups would result from exercise and dietary components.

Due to insufficient data, it was only possible to include two outcomes in the meta-analysis. Meta-analysis of the two trials (68 participants) reporting change from baseline to post-intervention WHR values revealed a small but statistically significant effect in favour of exercise and diet (MD − 0.02, 95% CI − 0.03 to − 0.01; *I*^2^ = 0%; Additional file 2: Figure S6). The effect was not replicated in the post-intervention value analysis.

We found no effect of exercise and diet combined versus control on the change from baseline to post-intervention SHBG concentrations (Additional file 2: Figure S7). There were insufficient data to complete analysis of post-intervention values or subgroups. Individual outcomes were also reported by each of these trials, which are summarised in Additional file 1: Table S10.

### Effects of interventions: Exercise and diet versus diet

Three trials had intervention arms that compared the combination of exercise and diet to diet only [33, 98, 104]. Analyses of change from baseline and post-intervention values from these trials revealed no statistical difference between combined exercise and diet or diet only interventions for any assessed primary outcome (FBG, FI, and HOMA-IR; all very low-quality evidence; Table 5) or secondary outcome (body weight, BMI, WC, body fat, fat-free mass, testosterone, SHBG, and FAI; Additional file 1: Table S11). There were insufficient data to complete subgroup analyses within this comparison.

**Table 5:** Summary of findings for primary outcomes: exercise and diet versus diet

Exercise and diet compared to Diet for women with PCOS						
Patient or population: women with PCOS Setting: Intervention: exercise and diet Comparison: Diet						
Outcomes	Anticipated absolute effects* (95% CI)		Relative effect (95% CI)	№ of participants (studies)	Certainty of the evidence (GRADE)	Comments
	Risk with Diet	Risk with exercise and diet				
Fasting blood glucose (change from baseline) follow-up: range 16 weeks to 20 weeks	The mean fasting blood glucose (change from baseline) ranged from − 7.0 to − 3.2 mg/dL	The mean fasting blood glucose (change from baseline) in the intervention group was 2.92 mg/dL higher (0.4 lower to 6.23 higher)	–	78 (2 RCTs)	⨁◯◯◯ VERY LOW ^a,b^	We are uncertain about the effect of exercise and diet on fasting blood glucose (change from baseline).
Fasting insulin (change from baseline) follow-up: range 12 weeks to 20 weeks	The mean fasting insulin (change from baseline) ranged from − 2.9 to − 18.54 μU/ml	The mean fasting insulin (change from baseline) in the intervention group was 2.22 μU/ml higher (3.7 lower to 8.14 higher)	–	90 (3 RCTs)	⨁◯◯◯ VERY LOW ^a,c,d^	We are uncertain about the effect of exercise and diet on fasting insulin (change from baseline).
HOMA-IR (change from baseline) follow-up: range 16 weeks to 20 weeks	The mean HOMA-IR (change from baseline) ranged from − 0.74 to − 0.56	The mean HOMA-IR (change from baseline) in the intervention group was 0.01 lower (0.45 lower to 0.43 higher)	–	78 (2 RCTs)	⨁◯◯◯ VERY LOW ^a,b^	We are uncertain about the effect of exercise and diet on HOMA-IR (change from baseline).
*The risk in the intervention group (and its 95% confidence interval) is based on the assumed risk in the comparison group and the relative effect of the intervention (and its 95% CI). CI: Confidence interval; MD: Mean difference						
GRADE Working Group grades of evidence High certainty: We are very confident that the true effect lies close to that of the estimate of the effect Moderate certainty: We are moderately confident in the effect estimate: The true effect is likely to be close to the estimate of the effect, but there is a possibility that it is substantially different Low certainty: Our confidence in the effect estimate is limited: The true effect may be substantially different from the estimate of the effect Very low certainty: We have very little confidence in the effect estimate: The true effect is likely to be substantially different from the estimate of effect						

Explanations

^a^All trials were at an unclear risk of selection bias, reporting bias, contamination, and adherence issues. All trials were at a high risk of detection bias and attrition bias. Therefore, we downgraded by one level

^b^Small number of participants, only two trials, and wide confidence intervals in the included trials. Therefore, we downgraded by two levels

^c^Substantial heterogeneity was observed. Therefore, we downgraded by one level

^d^Small number of participants and trials, wide confidence intervals, and null/negligible effect and appreciable benefit included in the confidence interval for the mean difference. Therefore, we downgraded by two levels

All three trials reported a range of other outcomes not included in this meta-analysis; these are summarised in Additional file 1: Table S12.

### Effects of interventions: Exercise vs diet, and exercise and diet vs exercise

Only one trial [105] compared exercise with diet, and exercise combined with diet versus exercise only. Effects in the diet only and combined diet and exercise group have been reported above and in Additional file 1: Table S12. The exercise-only intervention reduced BMI (− 0.85 kg/m^2^, 95% CI − 1.69 to − 0.02; *P* < .05), but these changes were smaller than those seen in the other treatment arms. Upper body fat was statistically reduced only in the exercise group (− 1.57 kg, 95% CI − 2.86 to − 0.28; *P* < .05) and mean follicle number exhibited the greatest improvement in the exercise-only group (*P* < .01). No within-group effects were reported for body fat (%), lower body fat (kg), lean body mass, free testosterone, insulin-like growth factor-1, insulin-like growth factor binding protein-1, FBG, FI, HOMA-IR, LH, FSH, testosterone, SHBG, T/SHBG ratio, AMH, or mean ovarian volume.

## Discussion

### Summary of the main results

Our systematic review provides up-to-date evidence supporting the incorporation of exercise interventions in the management of PCOS. When exercise was compared with control, we noted statistically beneficial changes from baseline to post-intervention and more favourable post-intervention values for FI, TC, LDL-C, and VO_2_ max. Statistically positive change from baseline scores was also observed for HOMA-IR, triglycerides, WC, and body fat percentage, whereas, statistically lower post-intervention values were additionally found for BMI and RHR. In an analysis of a limited number of studies, compared with control, a small statistical effect in favour of exercise and diet was evident for WHR, but not for SHBG. In the exercise and diet versus diet only comparison, we found no evidence of effect in any outcome; however, there were strikingly scant data available (Additional files 3 and Additional file 4).

#### Primary outcomes

We found a small change in SBP from baseline to post-intervention with supervised exercise versus control. To our knowledge, this is the first systematic review to report on the effects of exercise on blood pressure in women with PCOS. Existing evidence from the general population suggests that aerobic exercise interventions induce the greatest improvements to SBP and DBP in hypertensive participants [124], with less marked effects in normotensive participants (small decreases in DBP and no effect on SBP). The mean SBP (116 mmHg) and DBP (73 mmHg) values in our review indicates that most PCOS participants were normotensive at baseline; thus, a large effect was not anticipated.

Regarding surrogate markers of IR, we found a statistically beneficial change (FI and HOMA-IR) and more favourable post-intervention values (FI) with exercise compared with control. Subgroup analyses also indicate that the greatest improvements are noted in participants who were overweight or obese and from shorter duration, supervised aerobic-based interventions. These findings agree with those of two previous systematic reviews, which however, did not make the distinction between exercise, diet or their combination, but instead compared lifestyle interventions to control [125, 126]. The more recent of these reviews [125] reported a small, but statistically significant effect on FI change (MD − 2.1 μIU/mL, 95% CI − 3.3 to − 1.0; 5 trials, *I*^2^ = 0%). The other review [126] also compared the effect of lifestyle to a minimal treatment intervention on FI showing a statistical effect on FI post-intervention values favouring lifestyle (MD − 2.02 μIU/mL, 95% CI − 3.28 to − 0.77; 144 participants, 5 trials, *I*^2^ = 0%). Herein, we expanded on these previous findings by incorporating a greater number of trials and by separating exercise-only trials, thus revealing that based on the available data the exercise alone effects are comparable to that of lifestyle interventions.

Although the PCOS diagnostic criteria do not currently include IR, it is widely acknowledged that IR plays a key role in the pathophysiology of PCOS [127]. Approximately 50–70% of women with PCOS have IR and hyperinsulinaemia [128], whereas many also present evidence of glucose intolerance [20]. Hyperinsulinaemia in PCOS further promotes secretion of androgens from the ovarian theca cells, whilst supressing SHBG hepatic secretion, thus increasing free androgens and exacerbating the associated symptoms [129]. Despite the integral role of IR in PCOS, there are scant FI reference values in the literature [130]. One study [131] reported FI levels ranging from 2 to 60 μIU/mL in healthy women (*n* = 111), with a mean value of 17.6 ± 5.7 μIU/mL in women aged 25–34 years (*n* = 22). A large-scale case-control study of women with PCOS (*n* = 1404) reported mean FI levels of 14.3 ± 1.6 μIU/mL, which was significantly higher than healthy controls [132]. The mean baseline FI level of intervention participants in our review was 16.21 μIU/mL, and a reduction of ~ 13% was reported following exercise. Due to the variability of normative FI values in PCOS, it is unclear whether these exercise-induced reductions are clinically meaningful.

Although FI correlates with IR, several studies, especially in normoglycaemic populations [133, 134], have shown that HOMA-IR (calculated based on FI and FBG values) may be a better estimate of insulin sensitivity [135]. In the present review, the mean baseline HOMA-IR for the intervention group participants was 2.99, which dropped to 2.43 (MD − 0.57) following exercise, with no evidence of reduction in the control groups. A generally adopted HOMA-IR cut-off value for the identification of IR is 2.6 [136]. This suggests that exercise may have a clinically significant effect on IR compared with usual care. Furthermore, we found no effect of exercise on FBG. Participants were within normal FBG at baseline; thus, this combined with the effect on FI indicates that less insulin is needed to maintain normoglycaemia following exercise.

In contrast to previous reviews [126, 137], we report an effect of exercise on lipid profiles. Compared to control, there were improvements in exercise-induced changes for TC, LDL-C, and triglycerides. Based on data included in our review, the mean baseline values for TC (233 mg/dL) and LDL-C (142 mg/dL) would be classified as borderline high or even elevated in the presence of concomitant CVD risk factors [138]. Post-intervention values for LDL-C were lower for exercise compared to control, but TC levels were comparable (approximately 229 mg/dL in both). LDL-C appears to play a pivotal role in atherogenesis, with progressively increasing risk of coronary heart disease (CHD) with increasing LDL plasma levels [139]. Conversely, inverse associations between HDL-C and both atherosclerosis severity and CHD risk have been reported, with HDL-C levels ≥ 60 mg/dL potentially protecting against CHD [140]. HDL-C baseline and post-intervention values within this systematic review were > 60 mg/dL, which may partially explain why no effect of exercise was found. However, where TC and LDL-C are elevated at baseline, a statistical effect is evident following exercise but the magnitude of the changes may not be clinically important [141, 142].

Mean baseline triglyceride concentrations were higher in the exercise group (+ 11 mg/dL) compared with control, but both groups were within the normal range (< 150 mg/dL). Exercise reduced triglyceride levels, but post-intervention analysis revealed that concentrations were still lower in the control groups. Triglycerides are independent predictors of CVD mortality in women [143]; however, the magnitude of the observed exercise-induced triglyceride reduction, within the reported range, is likely to have little clinical relevance. Future research is required to investigate the independent effect of exercise in women with hypertriglyceridaemia.

#### Secondary outcomes

We found a statistically and clinically significant effect for VO_2_ max (> 3.5 ml/kg/min) with exercise compared with control. Subgroup analyses revealed that aerobic exercise, regardless of other variables, improved VO_2_ max in women with PCOS.

Low CRF, as measured by VO_2_ max, has been associated with increased risk of chronic disease and all-cause mortality [144, 145]. Reduction in VO_2_ max occurs physiologically with age, but is also often linked to inactivity. The consequences of reduced CRF include impaired capability to exercise, reduced ability to perform activities of daily living, and a lower overall quality of life [146]. Consequently, improving patient VO_2_ max is a goal of many lifestyle interventions yet is often overlooked in PCOS. Studies assessing VO_2_ max in this patient population are limited; two such studies in overweight [147] and lean [148] women with PCOS reveal markedly lower CRF than healthy controls. The only previous relevant systematic review to report on VO_2_ max/peak [137] found improvements for both lifestyle (i.e., exercise and diet combined; MD 5.09 ml/kg/min, 95% CI 3.13 to 7.05, 3 trials, 137 participants) and exercise (MD 4.86 ml/kg/min, 95% CI 2.83 to 6.88, 2 trials, 125 participants) interventions compared with usual care. Our analysis of relative VO_2_ max change pooled data from 92 more participants than the review by Haqq et al. [137], and although our effect was marginally smaller, the agreement between these results suggests that exercise can improve CRF in this population.

We also found reductions in WC and body fat in the exercise groups, suggesting that exercise promotes favourable changes to body composition in women with PCOS. As a measure of central/abdominal obesity, WC is considered a better independent predictor of obesity-related disorders than BMI [149]. This may be attributed to the key role of central adiposity in the development of IR and T2DM, even in those with normal BMI [150]. However, despite statistical significance, the exercise-induced WC changes may be of unclear clinical relevance, since the observed average reduction from baseline was 2.8% (95% CI 1.31 to 4.24), which is less than the suggested 3–5% reduction considered as clinically significant [151].

Improvements in anthropometric outcomes were reported by an older systematic review [137], but these were largely based on comparing lifestyle (not exercise alone) with control. Similarly, when compared to control, Moran et al. [126] reported statistical reductions in body weight and abdominal adiposity following lifestyle interventions. In our systematic review, when combined exercise and dietary interventions were compared with diet only, both groups demonstrated favourable changes, but there was no evidence of an effect favouring either intervention for any outcome.

We found no statistical effect of exercise on the androgenic profile of women with PCOS compared with control. Where analyses were possible, we found no effect favouring either diet and exercise combined or diet only. This was further supported by subgroup analyses where the evidence of relevant effects was minimal. Typically, the baseline values of women with PCOS included in this current review were below recommended cut-offs for diagnosing hyperandrogenism; testosterone > 2.5 nmol/L and SHBG < 30 nmol/L [152], which indicates that they were not markedly hyperandrogenic. Moran et al. [126] reported reduced testosterone levels following lifestyle intervention but found no effect on FAI (100 × total testosterone/SHBG), a more valid marker of hyperandrogenism [121]. A review of exercise-induced changes on the androgenic profile of healthy women who were premenopausal [153] found that exercise acutely increases circulating androgens, but the chronic effects are less clear. A similar meta-analysis [154] reported a chronic statistical reduction in concentrations of bioavailable testosterone (MD − 0.18 pg/mL, 95% CI − 0.29 to − 0.07; 1369 participants, 9 trials, *I*^2^ = 0%) and increased SHBG (MD 3.93 nmol/L, 95% CI 0.98 to 6.87; 1643 participants, 14 trials, *I*^2^ = 75%) following exercise in healthy women. Collectively, these data suggest that exercise interventions may regulate androgenic profiles, but that the optimal dose is unclear, with potential variation in women with menstrual disruption [155].

Finally, there is increasing recognition of the deleterious effects of PCOS on HRQoL and other psychosocial components. However, only three eligible trials measured these outcomes in the exercise versus control comparison. There was no evidence of effect in any of the PCOS-Q domains, but scores were improved in the physical functioning, general health, social functioning, and mental health domains of the SF-36. Our meta-analysis revealed improvements in these outcomes of ≥ 10% for exercise compared with control, supporting the notion that exercise in these patients may improve their perception of physical and mental wellbeing.

### Overall completeness and applicability of evidence

We completed a comprehensive and systematic search of relevant electronic databases and the reference lists from included publications and relevant reviews. From this, we identified 16 RCTs, one quasi-RCT, and a randomised crossover trial. We located and meta-analysed data from more trials, made a greater number of comparisons, and included a wider range of outcomes when compared to previous systematic reviews [25, 125, 126, 148]. To our knowledge, it is the first time data from 10 of the trials included in this systematic review have been meta-analysed [32, 34, 102, 104, 108–110, 117, 119] suggesting that this is the most comprehensive and up-to-date systematic review on the topic of exercise in the treatment of women with PCOS. We followed the PRISMA statement [26] and used the PRISMA checklist (Additional file 3) to ensure methodological quality. Furthermore, we present our entire data set for transparency and reproducibility in Additional file 4.

However, there are limitations to this systematic review. It is likely that many of the included trials were not sufficiently powered to detect meaningful differences between test groups. Indeed, only seven included trials state the methods used to calculate sample size, and due to small participant numbers (e.g., median: exercise *n* = 11; control *n* = 12), it is unlikely that sufficient statistical power was achieved to either make the findings generalisable into the population or ensure that false positive/negative results were not reported. Therefore, it is important that future trials are sufficiently powered to detect changes in their primary outcomes.

PCOS is a heterogeneous condition and can exhibit phenotypes with varying levels of underlying hyperandrogenism, menstrual disorders, and polycystic ovarian morphology [156]. It is likely that different phenotypes may respond differently to exercise and/or dietary interventions. Most included trials did not target a specific PCOS phenotype, and our protocol included a PCOS diagnosis based on any of the existing PCOS definitions/criteria. Future work should focus on PCOS subgroups/phenotypes and investigate the exercise-induced effects accordingly. Another concern surrounds the representativeness of the populations included in the review; it is not clear whether the ethnicity, socio-economic, or educational status of participants is representative of the typical patient or to what degree these variables may have influenced the observed effects.

All included trials reported baseline and immediately post-intervention data; only one trial [111] completed follow-up beyond the end of the intervention. Consequently, the lasting, long-term effect of exercise for women with PCOS is unknown. Future research is needed to determine whether behaviours relating to PA are changed in this patient population due to exercise interventions and whether the noted physiological effects remain beyond the short term.

### Quality of the evidence

Due to the nature of the interventions, all included trials were judged to have a high risk of performance bias. All but one trial was judged to have a high detection bias risk due to lack of blinding outcome assessors, and although logistically difficult, steps could have been taken to minimise this bias in each trial. Selection and reporting bias were inadequately reported in > 50% of trials so a judgement of unclear risk was made and nearly 45% of the included trials (*n* = 8) were judged to be at a high risk of attrition bias. Six trials were at an unclear or high risk of baseline group imbalance, whereas adherence and contamination were generally unreported resulting in an unclear judgement. Disappointingly, few studies reported adherence data (33%, *n* = 6), but of the trials that did report these data, adherence rates were generally good (median 90%). Similarly, in the 10 trials reporting attrition, the median value was 19.5%; five of these were under the 20% attrition threshold outlined in the protocol.

Statistical effects were reported in 13 of the main analyses; in three of those, there was evidence of at least substantial heterogeneity (*I*^2^ ≥ 50%), but this was largely explained by subgroups and/or removal of trials with the most extreme values. For our primary outcomes, the quality of evidence was rated as very low to low due to a combination of unclear or high-risk randomisation or allocation procedures, lack of blinding, unclear or improper handling of missing data, high attrition, unclear risk of selective reporting bias, contamination, low adherence, or considerable heterogeneity. We downgraded all outcomes because of imprecision resulting from the small number of participants and either wide confidence intervals for the effect estimate or the null effect, as well as an appreciable benefit was included in the confidence interval for the mean difference.

### Limitations and potential biases in the review process

In addition to the limitations mentioned in “Secondary outcomes” section, there are also further possible limitations to this systematic review. Despite a thorough and comprehensive search of relevant databases, we may have missed trials that would have been eligible for inclusion. Additionally, we did not identify any additional studies from the reference lists of the included publications; although this may support the comprehensiveness of our searches, it may also represent a methodological error. Also, no language restriction in our searches meant several foreign language papers were returned; three trials in Persian [104, 109, 110] and one in Hungarian [107]. To assess these trials, translation services and software were required, and whilst interpretation of results tables was straightforward, evaluation of methodological quality was more challenging. Consequently, when assessing risk of bias in these trials, judgements of ‘unclear risk’ had to be made.

Finally, only full publications were eligible for inclusion and this could contribute to publication bias. Although including grey literature may have influenced the findings of this review, it may have also increased the risk of associated bias. Unfortunately, due to a lack of eligible trials, publication bias analysis was not performed.

### Future directions

Based upon our findings, it is apparent that there is a lack of trials that compare exercise and diet combined with other comparators, such as diet only, exercise only, or a standard treatment control. Considering that lifestyle changes (i.e., diet and exercise) are recommended in the management of PCOS, studies assessing the effectiveness of these interventions are scarce and the available data are not sufficient to lead to definite conclusions/recommendations for the clinical practice. Future trials should aim to make comprehensive comparisons involving interventions that incorporate both exercise and diet.

Furthermore, the eligible studies included in the current systematic review generally have small sample sizes, whilst even those studies that have reported power calculations appear under-powered to detect meaningful changes in all reported outcomes. Therefore, it is important that future studies are robustly designed and sufficiently powered to better inform future clinical practice guidelines/recommendations. Considering the high prevalence of PCOS in reproductive-aged women, large RCTs studying the effectiveness of lifestyle interventions in this young patient population are still clearly needed.

We also identified a lack of follow-up testing beyond the intervention period to assess the longer-term effects of such lifestyle interventions. Without follow-up reassessments, it is impossible to determine whether any intervention-induced improvements are maintained, and if the applied intervention has resulted in sustained changes in lifestyle behaviours of participants, an aspect which is vital for the long-term management of these patients.

## Conclusion

When data were pooled in a meta-analysis, changes from baseline statistically favoured exercise over control for FI, HOMA-IR, TC, LDL-C, triglycerides, VO_2_ max, WC, and body fat percentage. Furthermore, a comparison of immediately post-intervention values also revealed statistical effects on FI, TC, LDL-C, VO_2_ max, RHR, and BMI. Compared with control, exercise also improved the physical functioning, general health, social functioning, and mental health domains assessed in the SF-36. Subgroup analyses revealed that the greatest favourable changes with exercise versus control were seen in participants who were either overweight (FI, HOMA-IR, triglycerides, VO_2_ max, and WC) or obese (BMI, body mass, and WC). Post-intervention value analyses also showed beneficial effects in those who were overweight (LDL-C, VO_2_ max, RHR, BMI, WC, and body fat percentage). Aerobic exercise interventions improved FI, HOMA-IR, TC, triglycerides, VO_2_ max, BMI, WC, and body fat percentage. In contrast, resistance training lowered HDL-C concentrations and increased BMI, but reduced WC; post-intervention improvements in HDL-C were also apparent following resistance exercise. Supervised exercise interventions improved outcomes more than unsupervised interventions compared with control. Shorter duration interventions performed better than longer interventions; improved change from baseline FI, HOMA-IR, TC, LDL-C, triglycerides, VO_2_ max, and WC was found in shorter duration trials, compared with only improved VO_2_ max in those > 12 weeks. Based on limited available data, we found no differences between the effects of exercise and diet combined and diet alone. Due to lack of available trials, it was not possible to compare the effectiveness of exercise versus diet or exercise and diet combined versus diet.

Although the evidence presented within this systematic review has largely been drawn from RCTs, a cautious approach should be adopted when interpreting the findings. Many of the outcomes presented modest effects and wide confidence intervals (indicating greater uncertainty). Furthermore, we found the statistical effects in many of the analyses to be sensitive to the addition or removal of individual trials regardless of their weighting within the analysis. Using the GRADE approach, we rated the quality of evidence as very low or low for all primary outcomes. Future trials should be rigorously designed and sufficiently powered so that they are more generalizable to the wider PCOS population. In order to be more closely aligned with current treatment recommendations, future studies should ideally include a dietary component alongside exercise interventions.

## Additional files

Additional file 1: **Table S1.** Search algorithm; **Table S2.** Review authors’ judgement on risk of bias; **Table S3.** Details of excluded studies and reasons for exclusion; **Table S4.** Diagnostic criteria/definition applied for PCOS; Table S5 Effect estimates and heterogeneity from subgroup analyses in blood pressure and metabolism-related outcomes; **Table S6.** Effect estimates and heterogeneity from subgroup analyses in lipid-related outcomes; **Table S7.** Effect estimates and heterogeneity from subgroup analyses in cardiorespiratory, anthropometric and body composition related outcomes; **Table S8.** Effect estimates and heterogeneity from subgroup analyses in androgenic and inflammatory outcomes; **Table S9.** Exercise vs control—Summary of findings from investigative outcomes that were only reported in single trials; **Table S10.** Exercise and diet vs control—Summary of findings from investigative outcomes that were only reported in single trials; **Table S11.** Effect estimates and heterogeneity for change from baseline to immediately post-intervention, and immediately post-intervention values only, for all outcomes analysed in the comparison exercise and diet versus diet only; **Table S12.** Exercise and diet vs diet—summary of findings from investigative outcomes that were only reported in single trials (PDF 296 kb)

Additional file 2: **Figure S1.** Review of authors’ judgements about each risk of bias item for each included study; **Figure S2.** Forest plot of comparison: exercise vs. control. Analysis of immediately post-intervention values for outcomes related to participant lipid profile; **Figure S3.** Forest plot of comparison: exercise vs. control. Analysis of resting heart rate subgroups by intervention type; **Figure S4.** Forest plot of comparison: exercise vs. control, change from baseline; outcome: SF-36 domains; **Figure S5.** Forest plot of comparison: exercise and diet vs. control; outcome: waist-to-hip ratio; **Figure S6.** Forest plot of comparison: exercise and diet vs. control, change from baseline. outcome: SHBG. (PDF 345 kb)

Additional file 3: PRISMA Checklist (PDF 202 kb)

Additional file 4: Additional meta-analyses/complete dataset. Supplementary meta-analyses: exercise vs. control; exercise and diet versus diet. (PDF 913 kb)

## Abbreviations

AMH: Anti-Mullerian hormone
BMI: Body mass index
CHD: Coronary heart disease
CIs: Confidence intervals
CRF: Cardiorespiratory fitness
CVD: Cardiovascular disease
DBP: Diastolic blood pressure
DHEA-S: Dehydroepiandrosterone sulfate
FAI: Free androgen index
FBG: Fasting blood glucose
FFM: Fat-free mass
FG: Ferriman-Gallwey score
FI: Fasting insulin
FSH: Follicle stimulating hormone
HDL-C: High-density lipoprotein cholesterol
HOMA-IR: Homeostatic model assessment of insulin resistance index
HR: Heart rate
HR_max_: Maximum heart rate
HRQoL: Health-related quality of life
hsCRP: High-sensitivity C-reactive protein
IR: Insulin resistance
LDL-C: Low-density lipoprotein cholesterol
LH: Luteinising hormone
MD: Mean difference
MET: Metabolic equivalent of task
PA: Physical activity
PCOS: Polycystic ovary syndrome
PCOS-Q: Polycystic ovary syndrome questionnaire
RCT: Randomised controlled clinical trials
RHR: Resting heart rate
SBP: Systolic blood pressure
SD: Standard deviation
SF-36: 36-item Short Form Survey
SHBG: Sex hormone binding globulin
SMD: Standardised mean difference
T2DM: Type 2 diabetes mellitus
TC: Total cholesterol
VO_2_ max: Maximal oxygen uptake
WC: Waist circumference
WHR: Waist-to-hip ratio
