# Supplementary material for: Exercise, or exercise and diet for the management of polycystic ovary syndrome: a systematic review and meta-analysis
Source: Syst Rev. 2019 Feb 12;8:51. doi: 10.1186/s13643-019-0962-3 (PMC6371542; doi:10.1186/s13643-019-0962-3)
Supplement: Supplementary file 1 — Table S1. Search algorithm; Table S2. Review authors’ judgement on risk of bias; Table S3. Details of excluded studies and reasons for exclusion; Table S4. Diagnostic criteria/definition applied for PCOS; Table S5 Effect estimates and heterogeneity from subgroup analyses in blood pressure and metabolism-related outcomes; Table S6. Effect estimates and heterogeneity from subgroup analyses in lipid-related outcomes; Table S7. Effect estimates and heterogeneity from subgroup analyses in cardiorespiratory, anthropometric and body composition related outcomes; Table S8. Effect estimates and heterogeneity from subgroup analyses in androgenic and inflammatory outcomes; Table S9. Exercise vs control—Summary of findings from investigative outcomes that were only reported in single trials; Table S10. Exercise and diet vs control—Summary of findings from investigative outcomes that were only reported in single trials; Table S11. Effect estimates and heterogeneity for change from baseline to immediately post-intervention, and immediately post-intervention values only, for all outcomes analysed in the comparison exercise and diet versus diet only; Table S12. Exercise and diet vs diet—summary of findings from investigative outcomes that were only reported in single trials (PDF 296 kb) [file 13643_2019_962_MOESM1_ESM.pdf]

Exercise and diet for the management of polycystic ovary syndrome: a systematic review and meta-analysis - Supplementary Data

Supplementary Table 1. Search algorithm as developed for advanced search in PubMed database. This algorithm was further adapted and implemented across CENTRAL (in the Cochrane Library), CINAHL, SCOPUS, EMBASE (via Web of Science), SportDiscus (via EBSCOhost) and PsycINFO (via OvidSP).

Search	Query
**#1**	Polycystic ovary syndrome [MeSH Terms]
**#2**	Polycystic ovar* [Title/Abstract]
**#3**	PCOS [Title/Abstract]
**#4**	PCOD [Title/Abstract]
**#5**	Stein levent* [Title/Abstract]
**#6**	PCO [Title/Abstract]
**#7**	#1 OR #2 OR #3 OR #4 OR #5 OR #6
**#8**	Exercise [MeSH Terms]
**#9**	Exercise movement techniques [MeSH Terms]
**#10**	Exercise Therapy [MeSH Terms]
**#11**	Exercise [Title/Abstract]
**#12**	Physical education and training [MeSH Terms]
**#13**	Physical fitness [MeSH Terms]
**#14**	Physical fitness [Title/Abstract]
**#15**	Physical exertion [MeSH Terms]
**#16**	Sports [MeSH Terms]
**#17**	Physical Activity [MeSH Terms]
**#18**	Sport* [Title/Abstract]
**#19**	Physical activity [Title/Abstract]
**#20**	Physical activities [Title/Abstract]
**#21**	Walking [MeSH Terms]
**#22**	Walk* [Title/Abstract]
**#23**	Resistance Training [MeSH Terms]
**#24**	Muscle training [Title/Abstract]
**#25**	Strength training [Title/Abstract]
**#26**	Endurance training [Title/Abstract]
**#27**	Interval training [Title/Abstract]
**#28**	Intermittent training [Title/Abstract]
**#29**	Fitness[Title/Abstract]
**#30**	Swimming [MeSH Terms]
**#31**	Swim* [Title/Abstract]
**#32**	Bicycling [MeSH Terms]
**#33**	Bicycl* [Title/Abstract]
**#34**	Cycling [Title/Abstract]
**#35**	Cycle [Title/Abstract]
**#36**	Strengthening [Title/Abstract]
**#37**	#8 OR #9 OR #10 OR #11 OR #12 OR #13 OR #14 OR #15 OR #16 OR #17 OR #18 OR #19 OR #20 OR #21 OR #22 OR #23 OR #24 OR #25 OR #26 OR #27 OR #28 OR #29 OR #30 OR #31 OR #32 OR #33 OR #34 OR #35 OR #36
**#38**	#7 AND #37
**#39**	Randomized controlled trial [Publication Type]
**#40**	Controlled clinical trial [Publication Type]

**#41**	Randomized [Title/Abstract]
**#42**	Placebo [Title/Abstract]
**#43**	Clinical trial as topic [MeSH Terms]
**#44**	Randomly [Title/Abstract]
**#45**	Trial [Title]
**#46**	#39 OR #40 OR #41 OR #42 OR #43 OR #44 OR #45
**#47**	#38 AND #46
**#48**	Animals [MeSH Major Topic] NOT Humans [MeSH Major Topic]
**#49**	#47 NOT #48

Supplementary Table 2. Review of authors' judgements about each risk of bias item for each included study. Support for judgement based upon evidence presented within each paper.

Trial	Bias Domain	Source of Bias	Author's judgement	Support for judgement
Almenning et al. [32]	Selection Bias	Random sequence generation	Low Risk	Women were stratified by BMI and allocated in a 1:1:1 manner to study arms. Computer number random generator developed and administered to randomise subjects.
		Allocation concealment	Low Risk	Baseline testing was done before randomisation
	Performance Bias	Blinding of participants and personnel	High Risk	Impossible to blind participants and personnel due to nature of the trial (*i.e.* supervised exercise sessions)
	Detection Bias	Blinding of outcome assessment	High Risk	Follow-up testing was performed, and these measurements were done non-blinded to group assignment. An observer blinded for group allocation analysed the FMD.
	Attrition Bias	Incomplete outcome data	Low Risk	89 participants assessed for eligibility; 58 excluded and reasons provided. 31 randomised and allocated 10:11:10. 6 (19%) lost to follow up (reasons provided) and data analysis completed on those remaining. Consort flow diagram used.
	Reporting Bias	Selective reporting	Low Risk	Trial preregistered on ClinicalTrials.gov (NCT01919281) and all proposed outcomes reported in paper.
	Other bias	Group similarity at baseline	Low Risk	FMD% significantly lower in HIT group. No other significant differences at baseline.
		Adherence	Low Risk	87% for RT arm and 90% for HIT arm.
		Contamination	Unclear Risk	Physical activity in control group not reported
Brown et al. [96]	Selection Bias	Random sequence generation	Low Risk	Randomisation was accomplished by generating a random sequence of two variables (representing the two treatment groups) using the online program at http://graphpadcom/quickcalcs/randomize 2.cfm

		Allocation concealment	Low Risk	Each group assignment was placed in its own sequentially numbered envelope by an individual not involved in the study. Participants were assigned to a group based on these envelopes, and each participant had an equal chance of being randomised to either group.
	Performance Bias	Blinding of participants and personnel	High Risk	Impossible to blind participants and personnel due to nature of the trial (i.e. supervised exercise sessions)
	Detection Bias	Blinding of outcome assessment	High Risk	Not reported
	Attrition Bias	Incomplete outcome data	High Risk	Attrition is reported in study but considerably greater in exercise group. Acknowledged as a limitation in study and reasons for attrition not clearly stated. Overall attrition reported as 43%.
	Reporting Bias	Selective reporting	Unclear Risk	Unable to locate prospectively published trial protocol
	Other bias	Group similarity at baseline	High Risk	Significant differences in age and lipid profiles. Also, although not statistically significant, exercisers tended to be heavier, less hyperandrogenic, less fit and more insulin resistant.
		Adherence	Low Risk	89.8% adherence to exercise reported
		Contamination	Unclear Risk	Physical activity in control group not reported
Bruner et al. [97]	Selection Bias	Random sequence generation	Unclear Risk	Not reported
		Allocation concealment	Low Risk	Researcher chose a sealed envelope for each participant indicating which treatment they would receive.
	Performance Bias	Blinding of participants and personnel	High Risk	Impossible to blind participants and personnel due to nature of the trial (*i.e.* supervised exercise sessions).
	Detection Bias	Blinding of outcome assessment	High Risk	Not reported
	Attrition Bias	Incomplete outcome data	High Risk	Attrition not reported. There are data missing from results; LH:FSH - 2 women in EN group (lab error & pregnancy); FI - 1 from EN & 1 from N (lab error).
	Reporting Bias	Selective reporting	Unclear Risk	Unable to locate prospectively published trial protocol.

	Other bias	Group similarity at baseline	Low Risk	No significant difference between groups for all outcomes.
		Adherence	Unclear Risk	Not reported
		Contamination	Unclear Risk	Physical activity in control group not reported
Guzick et al. [98]	Selection Bias	Random sequence generation	Unclear Risk	Subjects were randomised method used, not reported
		Allocation concealment	Unclear Risk	Not reported
	Performance Bias	Blinding of participants and personnel	High Risk	Impossible to blind participants and personnel due to nature of the trial (*i.e.* supervised exercise sessions).
	Detection Bias	Blinding of outcome assessment	High Risk	Not reported
	Attrition Bias	Incomplete outcome data	Low Risk	Reports those who were excluded during screening. 12 participants randomised; results for 12 presented in findings. No missing data.
	Reporting Bias	Selective reporting	Unclear Risk	Unable to locate prospectively published trial protocol.
	Other bias	Group similarity at baseline	Low Risk	No significant difference between treatment and control subjects for key outcomes of interest.
		Adherence	Unclear Risk	Not reported
		Contamination	Unclear Risk	Physical activity in control group not reported
Hoeger et al. [99]	Selection Bias	Random sequence generation	Low Risk	Randomisation schedule was computer generated in blocks by an independent pharmacy representative.
		Allocation concealment	Unclear Risk	The block schedule was blinded to the investigators. Methods not reported.
	Performance Bias	Blinding of participants and personnel	High Risk	Impossible to blind participants and personnel due to nature of the trial (*i.e.* supervised exercise sessions). However, participants and investigators double blinded to placebo or metformin by independent pharmacist.
	Detection Bias	Blinding of outcome assessment	High Risk	Not reported
	Attrition Bias	Incomplete outcome data	High Risk	Detailed analysis of attrition and adherence throughout. Balanced attrition across groups and explanations given for drop out. However, attrition is high in trial (39%).

	Reporting Bias	Selective reporting	Unclear Risk	Unable to locate prospectively published trial protocol.
	Other bias	Group similarity at baseline	Low Risk	No significant differences between groups for all outcomes.
		Adherence	Unclear Risk	Not reported
		Contamination	Unclear Risk	Physical activity in control group not reported
Konopka et al [101]	Selection Bias	Random sequence generation	Unclear Risk	Women were randomised but unclear what method was used to do this.
		Allocation concealment	Unclear Risk	Women were assessed before and after the intervention. Unclear when randomisation took place and whether investigators were blinded.
	Performance Bias	Blinding of participants and personnel	High Risk	Impossible to blind participants and personnel due to nature of the trial (*i.e.* supervised exercise sessions).
	Detection Bias	Blinding of outcome assessment	High Risk	Not reported
	Attrition Bias	Incomplete outcome data	High Risk	No attrition reported. However, hyperinsulinemic-euglycemic clamp only completed in a subset of obese women.
	Reporting Bias	Selective reporting	Low Risk	Trial preregistered on ClinicalTrials.gov (NCT02105428 and NCT01477164).
	Other bias	Group similarity at baseline	Low Risk	No significant differences between groups for all outcomes.
		Adherence	Unclear Risk	Not reported.
		Contamination	Unclear Risk	Physical activity in control group not reported.
Nasrekani et al. [103]	Selection Bias	Random sequence generation	Unclear Risk	Following eligibility screening and informed consent participants were randomised. Method of randomisation is not reported.
		Allocation concealment	Unclear Risk	Not reported whether assessors were blinded to allocation. Randomisation occurred before baseline measurements.
	Performance Bias	Blinding of participants and personnel	High Risk	Impossible to blind participants and personnel due to nature of the trial (*i.e.* supervised exercise sessions).
	Detection Bias	Blinding of outcome	High Risk	Not reported.

		assessment		
	Attrition Bias	Incomplete outcome data	Low Risk	20 participants randomised and all data reported. No use of Consort flow diagram.
	Reporting Bias	Selective reporting	Unclear Risk	Unable to locate prospectively published trial protocol.
	Other bias	Group similarity at baseline	Low Risk	No significant differences between groups for all outcomes.
		Adherence	Unclear Risk	Not reported.
		Contamination	Unclear Risk	Physical activity in control group not reported
Nybacka et al. [104-105]	Selection Bias	Random sequence generation	Low Risk	The randomisation was carried out with the permuted-block randomization method with ten blocks and a block size of 6.
		Allocation concealment	Unclear Risk	Not reported.
	Performance Bias	Blinding of participants and personnel	High Risk	Impossible to blind participants and personnel due to nature of the trial (*i.e.* supervised exercise sessions).
	Detection Bias	Blinding of outcome assessment	High Risk	Same investigators completed outcome assessments but unclear whether they were blinded to allocation. Blinding unlikely.
	Attrition Bias	Incomplete outcome data	High Risk	Attrition is reported for each arm. Higher in 2 groups. But overall 25%. Reasons stated as personal or medical grounds.
	Reporting Bias	Selective reporting	Unclear Risk	Unable to locate prospectively published trial protocol.
	Other bias	Group similarity at baseline	Low Risk	Baseline characteristics were comparable regarding age, BMI, body composition, and endocrine, metabolic and gynaecological outcomes.
		Adherence	Unclear Risk	Not reported.
		Contamination	Unclear Risk	Physical activity in control group not reported
Petranyi et al. [106]	Selection Bias	Random sequence generation	Unclear Risk	Participants were age matched between groups. Method of sequence generation not reported.
		Allocation concealment	Unclear Risk	Not reported.
	Performance Bias	Blinding of participants and personnel	High Risk	Impossible to blind participants and personnel due to nature of the trial (*i.e.* supervised exercise sessions).

	Detection Bias	Blinding of outcome assessment	High Risk	Not reported.
	Attrition Bias	Incomplete outcome data	Low Risk	Attrition not reported. 56 participants randomised and data present for all. No use of Consort (or similar) flow diagram.
	Reporting Bias	Selective reporting	Unclear Risk	Unable to locate prospectively published trial protocol.
	Other bias	Group similarity at baseline	Unclear Risk	Significance of baseline differences not reported. There appears to be some variation across outcomes.
		Adherence	Unclear Risk	Not reported.
		Contamination	Unclear Risk	Physical activity in control group not reported
Roessler et al. [34]	Selection Bias	Random sequence generation	Unclear Risk	Participants were randomised but the method used to generate sequence is not reported.
		Allocation concealment	Unclear Risk	Not reported.
	Performance Bias	Blinding of participants and personnel	High Risk	Impossible to blind participants and personnel due to nature of the trial (*i.e.* supervised exercise sessions).
	Detection Bias	Blinding of outcome assessment	High Risk	Not reported.
	Attrition Bias	Incomplete outcome data	Low Risk	Three participants did not complete – injury (not study related) and time concerns stated. Baseline data presented for all participants and separately for completers.
	Reporting Bias	Selective reporting	Unclear Risk	Unable to locate prospectively published trial protocol.
	Other bias	Group similarity at baseline	Low Risk	No significant difference between groups for all outcomes reported.
		Adherence	High Risk	Aerobic exercise adherence was 67%
		Contamination	High Risk	This was a crossover design. Control group received group counselling sessions that explored motivation for, and barriers to PA. Exercise not stated but likely that behaviour may have been influenced.
Sa et al. [107-108]	Selection Bias	Random sequence generation	High Risk	Randomisation sequence computer generated but allocation to exercise group was partially based on ability to attend the 16 weeks of training, which was

				limited for some participants (n = 5) due to their remote geographical location.
		Allocation concealment	High Risk	Five participants allocated to control group as they were unable to attend all sessions.
	Performance Bias	Blinding of participants and personnel	High Risk	Impossible to blind participants and personnel due to nature of the trial (*i.e.* supervised exercise sessions).
	Detection Bias	Blinding of outcome assessment	High Risk	Not reported.
	Attrition Bias	Incomplete outcome data	Low Risk	Consort flow diagram used; 30 randomised and baseline data presented for all in initial study. Post-intervention data presented for completers
	Reporting Bias	Selective reporting	High Risk	Unable to locate prospectively published trial protocol. A range of baseline outcomes reported, but no post-intervention analysis completed.
	Other bias	Group similarity at baseline	Low Risk	No significant differences between groups at baseline.
		Adherence	Unclear Risk	Not reported.
		Contamination	High Risk	Five participants in the control group did not receive the allocated intervention due to living in a remote geographical location
Saremi et al. [109]	Selection Bias	Random sequence generation	Unclear Risk	Quasi-randomisation. Methods used for sequence generation not reported.
		Allocation concealment	Unclear Risk	Not reported.
	Performance Bias	Blinding of participants and personnel	High Risk	Impossible to blind participants and personnel due to nature of the trial (*i.e.* supervised exercise sessions).
	Detection Bias	Blinding of outcome assessment	High Risk	Not reported.
	Attrition Bias	Incomplete outcome data	Low Risk	22 randomised and all post-intervention data present. No evidence of Consort flow diagram.
	Reporting Bias	Selective reporting	Unclear Risk	Unable to locate prospectively published trial protocol.
	Other bias	Group similarity at baseline	Unclear Risk	Significant differences not reported between groups. Some variability in data (HOMA-IR and lipid profile).

		Adherence	Unclear Risk	Not reported.
		Contamination	Unclear Risk	Physical activity in control group not reported
Saremi et al. [110]	Selection Bias	Random sequence generation	Unclear Risk	Method of randomisation and sequence generation not reported.
		Allocation concealment	Low Risk	Investigators were blinded to group allocation prior to baseline testing.
	Performance Bias	Blinding of participants and personnel	High Risk	Impossible to blind participants and personnel due to nature of the trial (*i.e.* supervised exercise sessions). However, allocation of placebo and calcium supplement was blinded to participant.
	Detection Bias	Blinding of outcome assessment	High Risk	Not reported.
	Attrition Bias	Incomplete outcome data	Unclear Risk	Attrition not reported. Consort flow diagram not presented. Number of participants randomised unclear.
	Reporting Bias	Selective reporting	Unclear Risk	Unable to locate prospectively published trial protocol.
	Other bias	Group similarity at baseline	Unclear Risk	Significant differences not reported between groups. Some variability in data (fasting insulin, blood glucose and lipid profile).
		Adherence	Unclear Risk	Not reported.
		Contamination	Unclear Risk	Physical activity in control group not reported
Stener-Victorin et al. [100, 102, 111-113]	Selection Bias	Random sequence generation	Low Risk	Randomly allocated in a 2:2:1 ratio to low-frequency EA, physical exercise, or no active intervention. To ensure equal proportions of age and BMI in each study arm, randomisation was stratified by those variables. Computer-generated randomisation within each stratum was conducted using permuted blocks of five.
		Allocation concealment	Unclear Risk	Allocation was concealed until interventions were assigned. Methods used, not reported.
	Performance Bias	Blinding of participants and personnel	High Risk	Impossible to blind participants and personnel due to nature of the trial (*i.e.* supervised exercise sessions and EA).
	Detection Bias	Blinding of outcome	High Risk	Not reported

		assessment		
	Attrition Bias	Incomplete outcome data	High Risk	Attrition data reported throughout each stage of the study. Comparable dropout in each arm of study and appropriate reasons provided. However, 29% attrition from randomisation to post-intervention and 40% to follow-up.
	Reporting Bias	Selective reporting	Low Risk	Trial preregistered on ClinicalTrials.gov (NCT00484705). 23 participants were recruited for microneurography; no criteria given for inclusion or detail on method of selection.
	Other bias	Group similarity at baseline	Low Risk	No significant differences between groups for all outcomes.
		Adherence	Low Risk	Mean number of weekly sessions reported; ~3 per week in PA group.
		Contamination	High Risk	There were no differences between the groups (PA, EA and control) in self-reports of PA frequency.
Thomson et al. [33, 114-116]	Selection Bias	Random sequence generation	Low Risk	A parallel study design where subjects were randomly assigned by computer generation into three 20-wk lifestyle interventions.
		Allocation concealment	Unclear Risk	Not reported
	Performance Bias	Blinding of participants and personnel	High Risk	Impossible to blind participants and personnel due to nature of the trial (*i.e.* supervised exercise sessions).
	Detection Bias	Blinding of outcome assessment	High Risk	Not reported.
	Attrition Bias	Incomplete outcome data	High Risk	Overview of reasons for dropout provided in study flow diagram but high rates reported – 49%
	Reporting Bias	Selective reporting	Unclear Risk	Unable to locate prospectively published trial protocol.
	Other bias	Group similarity at baseline	Low Risk	No significant differences between groups at baseline.
		Adherence	Unclear Risk	Not reported.
		Contamination	Unclear Risk	Physical activity in control group not reported
Turan et al. [117]	Selection Bias	Random sequence	Low Risk	A computer generated random number table was used to

		generation		generate sequence for allocation.
		Allocation concealment	Low Risk	Randomised following baseline testing. Allocation concealed using pre-labelled, sealed envelopes.
	Performance Bias	Blinding of participants and personnel	High Risk	Impossible to blind participants and personnel due to nature of the trial (i.e. supervised exercise sessions).
	Detection Bias	Blinding of outcome assessment	High Risk	Not reported.
	Attrition Bias	Incomplete outcome data	Low Risk	Small attrition (n = 2) from exercise group due to non-attendance of exercise sessions.
	Reporting Bias	Selective reporting	Unclear Risk	Unable to locate prospectively published trial protocol.
	Other bias	Group similarity at baseline	Low Risk	There were no significant differences between groups at baseline.
		Adherence	Low Risk	Two participants' data removed from analysis as adherence was < 75%
		Contamination	Unclear Risk	Physical activity in control group not reported
Vigorito et al. [118]	Selection Bias	Random sequence generation	Unclear Risk	Women were randomly subdivided into groups. Methods not reported.
		Allocation concealment	Unclear Risk	Not reported.
	Performance Bias	Blinding of participants and personnel	High Risk	Impossible to blind participants and personnel due to nature of the trial (i.e. supervised exercise sessions).
	Detection Bias	Blinding of outcome assessment	Low Risk	All clinical assessments were performed by the same physician who was blinded to the patient allocation into the study protocol.
	Attrition Bias	Incomplete outcome data	Low Risk	All subjects completed the study protocol.
	Reporting Bias	Selective reporting	Unclear Risk	Unable to locate prospectively published trial protocol.
	Other bias	Group similarity at baseline	Unclear Risk	Significant differences at baseline not reported. Patients share similar characteristics across groups.
		Adherence	Low Risk	All participants completed the study protocol. Attendance was 100% in exercise group.
		Contamination	Low Risk	Small decrease (-0.1 MET hr/wk) in LTPA for control group.

Vizza et al. [119]	Selection Bias	Random sequence generation	Low Risk	Randomisation assignments were generated via an online randomisation programme.
		Allocation concealment	Low Risk	Randomisation done by an investigator not involved in the data collection and given to participants in sealed envelopes upon completion of baseline testing.
	Performance Bias	Blinding of participants and personnel	High Risk	Impossible to blind participants and personnel due to nature of the trial (*i.e.* supervised exercise sessions).
	Detection Bias	Blinding of outcome assessment	High Risk	Clinical assessment completed by lead investigator - lead investigator also completed weekly status check with participants to monitor adverse events. Suggests no blinding.
	Attrition Bias	Incomplete outcome data	High Risk	15 participants randomised, 13% attrition across trial. Attrition detailed in results section and baseline data from non-completers used in results. Baseline data carried forward for two participants in the PRT and three in the control group that did not complete follow-up testing.
	Reporting Bias	Selective reporting	Unclear Risk	Unable to locate prospectively published trial protocol.
	Other bias	Group similarity at baseline	High Risk	Paper reports no significant difference between baseline characteristics of groups but does note trends in waist and hip circumference being higher in exercise group. Looking at the descriptive characteristics, mean body weight and BMI are considerably greater in the exercise group albeit with large standard deviations.
		Adherence	High Risk	Supervised training sessions have very good attendance (95%) but home-based component was only 51%.
		Contamination	Unclear Risk	Physical activity in control group not reported

BMI: body mass index; FMD: flow mediated dilation; HIT: high-intensity interval training; RT: resistance training; LH: luteinising hormone; FSH: follicle stimulating hormone; EN: exercise and nutrition; FI: fasting insulin; N: nutrition; PA: physical activity; HOMA-IR: homeostatic model assessment of insulin resistance index; EA: electroacupuncture; MET: metabolic equivalent of task; LTPA: leisure-time physical activity; PRT: progressive resistance training.

Supplementary Table 3. Details of excluded studies and reasons for exclusion.

Study	Reason for Exclusion
Almenning et al. [36]	Conference abstract; full study included in analysis
Asante et al. [37]	Conference abstract; full study included in analysis
Bachani et al. [38]	Comparison ineligible. Compares lifestyle modification with pharmacological intervention.
Barr et al. [39]	Ineligible study design. No intervention applied
Bongaard [40]	Comparison ineligible. Compares a low-GI diet with a moderate- to high-GI diet
Brown et al. [41]	Conference abstract; full study included in analysis
Chizen et al. [42]	Comparison ineligible. Compares a pulse diet with National Cholesterol Education Program Therapeutic Lifestyle Changes (TLC) Diet.
Christiansen et al. [43]	Ineligible patient population. Pregnant women.
Crosignani et al. [44]	Ineligible study design. Not a randomised controlled trial.
Curi et al. [45]	Comparison ineligible. Compares lifestyle modification with pharmacological intervention.
Fux Otta et al. [46]	Comparison ineligible. Compared lifestyle and pharmacological intervention with lifestyle and placebo.
Galletly et al. [47]	Comparison ineligible. Compares a low-protein high-carbohydrate diet with a high-protein low-carbohydrate diet.
Gambineri et al. [48]	Comparison ineligible. Compares a hypocaloric diet with placebo to a hypocaloric diet with pharmacological interventions.
Giallauria et al. [49]	Ineligible study design. Patients not randomised.
Harris-Glocker et al. [50]	Ineligible comparison. Compares lifestyle modification and placebo with lifestyle modification and pharmacological intervention.
Hoeger et al. [51]	Ineligible patient population. Adolescent patients were used in the trial.
Jaffe et al. [52]	Comparison ineligible. Compares high-carbohydrate low-fat diet and placebo with high-carbohydrate low-fat diet and pharmacological intervention.
Jedel et al. [53]	Conference abstract; full study included in analysis
Johansson et al. [54]	Ineligible comparison. Compares acupuncture to physical therapy.
Karimzadeh et al. [55]	Comparison ineligible. Compares lifestyle to pharmacological interventions.
Kumar et al. [56]	Comparison ineligible. Compares lifestyle to lifestyle and pharmacological interventions.
Ladson et al. [57]	Comparison ineligible. Compares lifestyle to lifestyle and pharmacological interventions.
Ladson et al. [58]	Comparison ineligible. Compares lifestyle and placebo to lifestyle and pharmacological interventions.
Le Donne et al. [59]	Comparison ineligible. Compares diet with diet and pharmacological interventions.
Legro et al. [60]	Comparison ineligible. Compares lifestyle with lifestyle and pharmacological interventions.
Legro et al. [61]	Comparison ineligible. Compares lifestyle with lifestyle and pharmacological interventions.

Liao et al. [62]	Ineligible study design. Observational design.
Lindholm et al. [63]	Comparison ineligible. Compares lifestyle and placebo to lifestyle and pharmacological interventions.
Ma et al. [64]	Ineligible comparison. Compares weight loss treatment to weight loss treatment and pharmacological intervention.
Machlitt et al. [65]	Ineligible comparison. Compares lifestyle and placebo with lifestyle and pharmacological intervention.
Marzouk et al. [66]	Ineligible intervention. Intervention use dietary advice and caloric restriction.
McBreairty et al. [67]	Ineligible comparison. Compares pulse based diet and exercise with National Cholesterol Education Program therapeutic lifestyle changes (TLC) diet and exercise.
Mehrabani et al. [68]	Ineligible intervention. Two hypocaloric diets are used for the intervention.
Moran et al. [69]	Ineligible intervention. Low-protein and a high-protein hypocaloric diets are used as the intervention.
Moran et al. [70]	Ineligible intervention. Meal replacement programme utilised; no comparison made.
Moran et al. [71]	Ineligible intervention. Two diets are utilised in the intervention.
Nidhi et al. [72]	Ineligible patient population. Adolescent patients were used in the trial. Also compares two exercise modalities.
Nidhi et al. [73]	Ineligible patient population. Adolescent patients were used in the trial. Also compares two exercise modalities.
Nidhi et al. [74]	Ineligible patient population. Adolescent patients were used in the trial.
Nybacka et al. [75]	Conference abstract; full study included in analysis
Nybacka et al. [76]	Conference abstract; full study included in analysis
Omar et al. [77]	Ineligible patient population. Compares women with PCOS to healthy controls.
Orio et al. [78]	Ineligible comparison. Compares lifestyle to pharmacological interventions.
Ornstein et al. [79]	Ineligible patient population. Adolescent patients were used in the trial.
Palomba et al. [80]	Ineligible comparison. Compares lifestyle to lifestyle and pharmacological interventions.
Palomba et al. [81]	Ineligible study design. Non-randomised controlled trial.
Papakonstantinou et al. [82]	Ineligible intervention. Compares two dietary interventions in a cross-over design.
Pasquali et al. [83]	Ineligible comparison. Compares hypocaloric diet with diet and pharmacological intervention.
Pasquali et al. [84]	Ineligible patient population. Compares hypocaloric diet with diet and pharmacological intervention.
Popova et al. [85]	Ineligible comparison. Compares lifestyle with lifestyle and pharmacological interventions.
Randeva et al. [86]	Ineligible study design. Non-randomised controlled trial.
Redman et al. [87]	Ineligible patient population. Women with PCOS are compared to healthy controls.
Roessler et al. [88]	Conference abstract; full study included in analysis
Silva Dantas et al. [89]	Ineligible patient population. Compares women with PCOS to healthy controls.
Sorensen et al. [90]	Ineligible intervention. Either a high- or standard-protein diet are used for the intervention.
Tang et al. [91]	Ineligible comparison. Compares lifestyle and placebo with lifestyle and pharmacological intervention.

Thomson et al. [92]	Conference abstract; full study included in analysis
Thomson et al. [93]	Ineligible study design. No control group or comparison made.
Turner-McGrievy et al. [94]	Ineligible study design. No lifestyle intervention
Turner-McGrievy et al. [95]	Ineligible comparison. Compares vegan diet to low-calorie diet.

Supplementary Table 4. Diagnostic criteria/definition applied for polycystic ovary syndrome (PCOS).

**PCOS Criteria**	**NIH (1990)***	**Rotterdam ESHRE/ASRM (2003)***	**AE-PCOS Society (2006)***
	Must have both of the findings marked below.	*Must have at least two of the findings marked below.*	*Must have marked finding with either/or both of the other two.*
Hyperandrogenism (clinical or biochemical findings)	√	√	√
Oligomenorrhea	√	√	
Polycystic Ovarian Morphology		√	

*In addition to the above criteria, PCOS diagnosis requires the exclusion of other androgen excess or related disorders, including: hyperprolactinaemia, thyroid dysfunction, adrenal hyperplasia, androgen secreting tumours, and Cushing's syndrome.

Supplementary Table 5. Summary of effect estimates and heterogeneity from sub-group analyses in blood pressure and metabolism-related outcomes.

Outcome	Sub-analysis	Sub-group	Change from baseline			Post-intervention		
			Trials (*N*)	Effect Estimate MD (95% CI)	I^2 (%)	Trials (*N*)	Effect Estimate MD (95% CI)	I^2 (%)
SBP (mmHg)	BMI at entry	≤ 24.9 kg/m^2	1 (30)	-0.20 (-6.51 to 6.11)	NA	1 (30)	9.70 (4.35 to 15.05)	NA
		25-29.9 kg/m^2	2 (101)	-1.80 (-8.53 to 4.93)	65	2 (101)	0.19 (-12.94 to 13.31)	82
		≥ 30 kg/m^2	1 (27)	-7.70 (-13.73 to -1.67)	NA	1 (27)	-2.80 (-11.23 to 5.63)	NA
	Intervention type	Aerobic exercise	3 (128)	-3.71 (-8.88 to 1.47)	60	3 (128)	-1.41 (-8.65 to 5.82)	65
		Combined exercise	1 (30)	-0.20 (-6.51 to 6.11)	NA	1 (30)	9.70 (4.35 to 15.05)	NA
	Intervention duration	≤12 weeks	2 (120)	-3.03 (-7.54 to 1.47)	27	2 (120)	1.90 (-13.19 to 16.99)	95
		>12 weeks	2 (38)	-2.91 (-12.41 to 6.60)	79	2 (38)	2.06 (-8.29 to 12.41)	59
	Delivery format	Supervised	3 (147)	-4.42 (-8.32 to -0.51) *	31	3 (147)	0.45 (-10.04 to 10.94)	90
		Unsupervised	1 (11)	2.00 (-4.39 to 8.39)	NA	1 (11)	7.80 (-2.54 to 18.14)	NA
DBP (mmHg)	BMI at entry	≤ 24.9 kg/m^2	1 (30)	-0.20 (-7.23 to 6.83)	NA	1 (30)	4.50 (-1.43 to 10.43)	NA
		25-29.9 kg/m^2	2 (101)	-1.19 (-3.11 to 0.73)	0	2 (101)	-1.31 (-3.09 to 0.47)	0
		≥ 30 kg/m^2	1 (27)	-8.10 (-13.80 to -2.40)	NA	1 (27)	-2.60 (-8.59 to 3.39)	NA
	Intervention type	Aerobic exercise	3 (128)	-2.67 (-6.50 to 1.17)	62	3 (128)	-1.41 (-3.12 to 0.29)	0
		Combined exercise	1 (30)	-0.20 (-7.23 to 6.83)	NA	1 (30)	4.50 (-1.43 to 10.43)	NA
	Intervention duration	≤12 weeks	2 (120)	-1.30 (-3.31 to 0.71)	0	2 (120)	0.96 (-4.33 to 6.26)	68
		>12 weeks	2 (38)	-3.95 (-11.78 to 3.89)	77	2 (38)	-3.16 (-7.54 to 1.22)	0
	Delivery format	Supervised	3 (147)	-3.03 (-7.36 to 1.30)	60	3 (147)	-0.22 (-3.50 to 3.07)	43
		Unsupervised	1 (11)	-0.10 (-4.90 to 4.70)	NA	1 (11)	-3.80 (-10.22 to 2.62)	NA
FBG (mg/dL)	BMI at entry	≤ 24.9 kg/m^2	1 (30)	-4.00 (-8.94 to -0.94)	NA	1 (30)	-3.70 (-10.08 to 2.68)	NA
		25-29.9 kg/m^2	5 (168)	-0.79 (-2.08 to 0.50)	0	5 (168)	-1.59 (-5.29 to 2.11)	62
		≥ 30 kg/m^2	3 (65)	-0.87 (-8.95 to 7.22)	58	2 (40)	-1.21 (-8.83 to 6.41)	0
	Intervention type	Aerobic exercise	6 (192)	-0.70 (-2.46 to 1.05)	21	5 (167)	-0.83 (-2.80 to 1.13)	0
		Resistance exercise	3 (50)	-1.01 (-3.37 to 1.34)	11	3 (50)	-3.81 (-13.74 to 6.11)	76
		Combined exercise	1 (30)	-4.00 (-8.94 to 0.94)	NA	1 (30)	-3.70 (-10.08 to 2.68)	NA
	Intervention duration	≤12 weeks	7 (225)	-1.47 (-3.03 to 0.10)	18	6 (200)	-2.18 (-5.82 to 1.46)	53
		>12 weeks	2 (38)	0.38 (-2.42 to 3.19)	0	2 (38)	-0.33 (-4.29 to 3.64)	0
	Delivery	Supervised	6 (214)	-1.75 (-4.06 to 0.56)	40	5 (189)	-3.04 (-7.59 to 1.52)	60

	format	Unsupervised	1 (11)	0.00 (-2.91 to 2.91)	NA	1 (11)	0.00 (-4.27 to 4.27)	NA
		Mixed Delivery	2 (38)	-0.73 (-3.05 to 1.58)	0	2 (38)	0.00 (-4.91 to 4.91)	0
FI (µIU/mL)	BMI at entry	≤ 24.9 kg/m^2	1 (30)	-1.00 (-1.44 to 0.56)	NA	1 (30)	-0.60 (-3.11 to 1.91)	NA
		25-29.9 kg/m^2	5 (168)	-3.25 (-5.27 to -1.22) **	75	5 (168)	-2.27 (-3.24 to -1.31) ***	0
		≥ 30 kg/m^2	3 (65)	-1.94 (-3.94 to 0.06)	0	2 (40)	1.30 (-14.29 to 16.89)	88
	Intervention type	Aerobic exercise	6 (192)	-2.22 (-3.57 to -0.86) ***	10	5 (167)	-2.48 (-3.92 to -1.04) ***	10
		Resistance exercise	3 (50)	-3.99 (-5.97 to -2.00) ***	54	3 (50)	-0.24 (-6.99 to 6.51)	68
		Combined exercise	1 (30)	-1.00 (-1.44 to -0.56)	NA	1 (30)	-0.60 (-3.11 to 1.91)	NA
	Intervention duration	≤12 weeks	7 (225)	-2.92 (-4.91 to -0.93) **	93	6 (200)	-1.80 (-3.18 to -0.42) **	32
		>12 weeks	2 (38)	0.06 (-2.87 to 2.99)	0	2 (38)	-3.63 (-8.11 to 0.85)	67
	Delivery format	Supervised	6 (214)	-2.54 (-4.82 to -0.26) *	94	5 (189)	-2.39 (-3.62 to -1.17) ***	30
		Unsupervised	1 (11)	-0.20 (-3.38 to 2.98)	NA	1 (11)	-1.40 (-4.89 to 2.09)	NA
		Mixed Delivery	2 (38)	-3.08 (-5.63 to -0.53) *	17	2 (38)	3.54 (-8.29 to 15.37)	71
HOMA-IR	BMI at entry	≤ 24.9 kg/m^2	1 (30)	0.20 (-0.53 to 0.93)	NA	1 (30)	-0.20 (-0.75 to 0.35)	NA
		25-29.9 kg/m^2	4 (78)	-0.83 (-1.39 to -0.26) **	75	4 (78)	-0.51 (-1.10 to 0.07)	55
		≥ 30 kg/m^2	3 (65)	-0.43 (-1.19 to 0.32)	87	2 (40)	0.71 (-1.47 to 2.88)	55
	Intervention type	Aerobic exercise	5 (102)	-0.73 (-1.24 to -0.21) **	60	4 (77)	-0.15 (-0.70 to 0.40)	0
		Resistance exercise	3 (50)	-0.74 (-1.58 to 0.10)	94	3 (50)	-0.24 (-1.89 to 1.41)	85
		Combined exercise	1 (30)	0.20 (-0.53 to 0.93)	NA	1 (30)	-0.20 (-0.75 to 0.35)	NA
	Intervention duration	≤12 weeks	6 (135)	-0.69 (-1.13 to -0.26) **	89	5 (110)	-0.14 (-0.88 to 0.59)	78
		>12 weeks	1 (11)	0.10 (-0.61 to 0.81)	NA	1 (11)	-0.30 (-1.07 to 0.47)	NA
	Delivery format	Supervised	5 (124)	-0.80 (-1.19 to -0.42) ***	76	4 (99)	-0.46 (-1.09 to 0.17)	66
		Unsupervised	1 (11)	0.10 (-0.61 to 0.81)	NA	1 (11)	-0.30 (-1.07 to 0.47)	NA
		Mixed Delivery	2 (38)	-0.55 (-1.60 to 0.50)	77	2 (38)	0.47 (-1.49 to 2.42)	66

Outcome: outcome where sub-analysis was completed. Sub-analysis: how the studies were categorised for analysis. Sub-group: groups each study was classified into. Trials: number of studies included within sub-analysis, *N*: number or participants included within sub-analysis. Effect estimates are reported as mean difference (MD), and 95% confidence intervals, between exercise and control groups. Significant evidence of effect denoted by: * $P \leq 0.05$; ** $P \leq 0.01$; *** $P \leq 0.001$. Heterogeneity reported using I^2 statistic: 0-40% might not be important; 30-60% may represent moderate heterogeneity; 50-90% may represent substantial heterogeneity; 75-100% may represent considerable heterogeneity. NA: I^2 not applicable. SBP: systolic blood pressure; DBP: diastolic blood pressure; FBG: fasting blood glucose; FI: fasting insulin; BMI = body mass index.

Supplementary Table 6. Summary of effect estimates and heterogeneity from sub-group analyses in lipid related outcomes

Outcome	Sub-analysis	Sub-group	Change from baseline			Post-intervention		
			**Trials (*N*)**	**Effect Estimate MD (95% CI)**	**I^2 (%)**	**Trials (*N*)**	**Effect Estimate MD (95% CI)**	**I^2 (%)**
Triglycerides (mg/dL)	BMI at entry	≤ 24.9 kg/m^2	1 (30)	-4.20 (-6.87 to -1.53)	NA	1 (30)	-2.30 (-6.34 to 1.74)	NA
		25-29.9 kg/m^2	5 (168)	-8.17 (-14.44 to -1.89) **	13	5 (168)	3.04 (-4.97 to 11.05)	0
		≥ 30 kg/m^2	1 (27)	-5.04 (-16.10 to 6.02)	NA	1 (27)	-14.95 (-28.74 to -1.16)	NA
	Intervention type	Aerobic exercise	5 (167)	-6.80 (-13.12 to -0.48) *	5	5 (167)	-2.89 (-14.44 to 8.65)	41
		Resistance exercise	2 (37)	-9.91 (-22.32 to 2.49)	0	2 (37)	6.05 (-12.08 to 24.19)	0
		Combined exercise	1 (30)	-4.20 (-6.87 to -1.53)	NA	1 (30)	-2.30 (-6.34 to 1.74)	NA
	Intervention duration	≤12 weeks	5 (187)	-6.06 (-10.82 to -1.31) **	30	5 (187)	-1.10 (-4.73 to 2.54)	0
		>12 weeks	2 (38)	-6.18 (-15.44 to 3.09)	0	2 (38)	-13.85 (-26.33 to -1.36) *	0
	Delivery format	Supervised	5 (189)	-5.91 (-10.75 to -1.06) *	29	5 (189)	-2.49 (-6.77 to 1.79)	7
		Unsupervised	1 (11)	-8.85 (-25.81 to 8.11)	NA	1 (11)	8.85 (-38.22 to 20.52)	NA
		Mixed Delivery	1 (25)	-6.20 (-16.12 to 3.73)	NA	1 (25)	20.36 (-5.87 to 46.58)	NA
TC (mg/dL)	BMI at entry	≤ 24.9 kg/m^2	1 (30)	-3.70 (-6.26 to -1.14)	NA	1 (30)	-5.60 (-15.40 to 4.20)	NA
		25-29.9 kg/m^2	5 (168)	-6.30 (-12.81 to 0.21)	41	5 (168)	-4.16 (-10.31 to 2.00)	0
		≥ 30 kg/m^2	1 (27)	-12.08 (-24.34 to 0.18)	NA	1 (27)	-10.81 (-19.04 to -2.58)	NA
	Intervention type	Aerobic exercise	5 (167)	-6.68 (-13.00 to -0.35) *	39	5 (167)	-6.90 (-11.90 to -1.90) **	0
		Resistance exercise	2 (37)	-9.72 (-21.67 to 2.22)	0	2 (37)	6.47 (-16.70 to 29.63)	0
		Combined exercise	1 (30)	-3.70 (-6.26 to -1.14)	NA	1 (30)	-5.60 (-15.40 to 4.20)	NA
	Intervention duration	≤12 weeks	5 (187)	-5.94 (-10.32 to -1.55) **	37	5 (187)	-4.74 (-10.05 to 0.57)	0
		>12 weeks	2 (38)	-4.78 (-20.35 to 10.80)	61	2 (38)	-9.92 (-17.81 to -2.04) **	0
	Delivery format	Supervised	5 (189)	-7.25 (-11.92 to -2.58) **	48	5 (189)	-6.76 (-11.27 to -2.26) **	0
		Unsupervised	1 (11)	3.87 (-11.38 to 19.12)	NA	1 (11)	0.00 (-27.50 to 27.50)	NA
		Mixed Delivery	1 (25)	-0.01 (-14.95 to 14.93)	NA	1 (25)	5.80 (-26.26 to 37.86)	NA
LDL-C (mg/dL)	BMI at entry	≤ 24.9 kg/m^2	1 (30)	-8.72 (-11.69 to -5.75)	NA	1 (30)	-4.60 (-16.07 to 6.87)	NA
		25-29.9 kg/m^2	5 (168)	-3.41 (-8.05 to 1.24)	0	5 (168)	-9.54 (-18.71 to -0.36) *	22
		≥ 30 kg/m^2	1 (27)	-11.71 (-22.96 to -0.46)	NA	1 (27)	-5.59 (-13.91 to 2.73)	NA
	Intervention type	Aerobic exercise	5 (167)	-4.17 (-9.23 to 0.90)	0	5 (167)	-5.87 (-11.68 to -0.07) *	0
		Resistance exercise	2 (37)	-6.50 (-16.32 to 3.32)	22	2 (37)	-13.57 (-38.44 to 11.29)	45
		Combined exercise	1 (30)	-8.72 (-11.69 to -5.75)	NA	1 (30)	-4.60 (-16.07 to 6.87)	NA
	Intervention	≤12 weeks	5 (187)	-6.60 (-9.88 to -3.32) ***	13	5 (187)	-8.64 (-16.30 to -0.98) *	22

	duration	>12 weeks	2 (38)	-8.62 (-17.37 to 0.14)	0	2 (38)	5.05 (-12.97 to 2.86)	0
	Delivery format	Supervised	5 (187)	-6.70 (-10.29 to -3.12) ***	23	5 (187)	-7.58 (-13.73 to -1.43) *	24
		Unsupervised	1 (11)	-3.87 (-17.81 to 10.07)	NA	1 (11)	0.00 (-25.55 to 25.55)	NA
		Mixed Delivery	1 (25)	-7.72 (-22.99 to 7.55)	NA	1 (25)	-5.80 (-38.38 to 26.78)	NA
HDL-C▲ (mg/dL)	BMI at entry	≤ 24.9 kg/m^2	1 (30)	0.60 (-0.40 to 1.60)	NA	1 (30)	-0.90 (-3.19 to 1.39)	NA
		25-29.9 kg/m^2	5 (168)	0.99 (-2.89 to 4.88)	61	5 (168)	3.36 (-3.33 to 10.05)	62
		≥ 30 kg/m^2	1 (27)	0.36 (-2.11 to 2.83)	NA	1 (27)	1.44 (-1.28 to 4.16)	NA
	Intervention type	Aerobic exercise	5 (167)	2.69 (-1.47 to 6.86)	59	5 (167)	1.04 (-3.06 to 5.15)	29
		Resistance exercise	2 (37)	-2.19 (-4.21 to -0.18) *	0	2 (37)	7.29 (1.11 to 13.46) *	17
		Combined exercise	1 (30)	0.60 (-0.40 to 1.60)	NA	1 (30)	-0.90 (-3.19 to 1.39)	NA
	Intervention duration	≤12 weeks	5 (187)	-0.10 (-2.27 to 2.08)	57	5 (187)	2.83 (-2.73 to 8.40)	76
		>12 weeks	2 (38)	2.93 (-3.96 to 9.82)	64	2 (38)	1.25 (-1.42 to 3.92)	0
	Delivery format	Supervised	5 (189)	-0.32 (-1.87 to 1.23)	45	5 (189)	1.93 (-1.86 to 5.72)	75
		Unsupervised	1 (11)	7.74 (-0.61 to 16.09)	NA	1 (11)	-3.86 (-17.97 to 10.25)	NA
		Mixed Delivery	1 (25)	4.25 (-1.43 to 9.93)	NA	1 (25)	7.74 (-7.42 to 22.90)	NA

Outcome: outcome where sub-analysis was completed. Sub-analysis: how the studies were categorised for analysis. Sub-group: groups each study was classified into. Trials: number of studies included within sub-analysis, *N*: number or participants included within sub-analysis. Effect estimates are reported as mean differences (MD), and 95% confidence intervals (CI), between exercise and control groups. ▲: positive values favour exercise over control. Significant evidence of effect denoted by: * $P \leq 0.05$; ** $P \leq 0.01$; *** $P \leq 0.001$. Heterogeneity reported using I^2 statistic: 0-40% might not be important; 30-60% may represent moderate heterogeneity; 50-90% may represent substantial heterogeneity; 75-100% may represent considerable heterogeneity. NA: I^2 not applicable. BMI: body mass index; TC: total cholesterol; LDL-C: low-density lipoprotein cholesterol; HDL-C: high-density lipoprotein cholesterol.

Supplementary Table 7. Summary of effect estimates and heterogeneity from sub-group analyses in cardiorespiratory, anthropometric and body composition related outcomes.

Outcome	Sub-analysis	Sub-group	Change from baseline			Post-intervention		
			Trials (*N*)	Effect Estimate MD (95% CI)	I^2 (%)	Trials (*N*)	Effect Estimate MD (95% CI)	I^2 (%)
VO_2 max/peak▲ (ml/kg/min)	BMI at entry	25-29.9 kg/m^2	5 (202)	3.39 (2.66 to 4.13) ***	0	4 (157)	4.70 (2.90 to 6.49) ***	51
		≥ 30 kg/m^2	1 (27)	5.70 (3.10 to 8.30)	NA	1 (27)	6.90 (3.13 to 10.67)	NA
	Intervention type	Aerobic exercise	6 (221)	4.11 (3.07 to 5.14) ***	21	5 (176)	5.05 (3.53 to 6.56) ***	41
		Resistance exercise	1 (17)	1.70 (-1.16 to 4.56)	NA	1 (17)	4.20 (-3.22 to 11.62)	NA
	Intervention duration	≤12 weeks	4 (157)	3.35 (2.59 to 4.10) ***	0	4 (157)	4.70 (2.90 to 6.49) ***	51
		>12 weeks	2 (72)	5.17 (3.11 to 7.23) ***	0	1 (27)	6.90 (3.13 to 10.67)	NA
	Delivery format	Supervised	4 (159)	4.43 (2.76 to 6.10) ***	47	4 (159)	5.04 (3.25 to 6.82) ***	56
		Mixed Delivery	1 (25)	3.10 (0.79 to 5.41)	NA	1 (25)	4.65 (-0.84 to 10.14)	NA
Resting Heart Rate (beats/min)	BMI at entry	≤ 24.9 kg/m^2	1 (30)	-7.12 (-13.37 to -0.87)	NA	1 (30)	-6.90 (-13.88 to 0.08)	NA
		25-29.9 kg/m^2	3 (126)	-1.76 (-4.41 to 0.89)	58	3 (126)	-3.04 (-4.76 to -1.32) ***	10
	Intervention type	Aerobic exercise	3 (118)	-3.32 (-5.50 to -1.15) **	0	3 (118)	-3.00 (-4.72 to -1.28) ***	0
		Resistance exercise	1 (17)	3.50 (-1.01 to 8.01)	NA	1 (17)	-0.10 (-9.16 to 8.96)	NA
		Combined exercise	1 (30)	-7.12 (-13.37 to -0.87)	NA	1 (30)	-6.90 (-13.88 to 0.08)	NA
	Intervention duration	≤12 weeks	3 (145)	-2.54 (-5.90 to 0.81)	66	3 (145)	-3.18 (-5.59 to -0.77) **	16
		>12 weeks	1 (11)	-4.50 (-12.93 to 3.93)	NA	1 (11)	-5.70 (-20.72 to 9.32)	NA
	Delivery format	Supervised	2 (120)	-4.06 (-7.42 to -0.70) *	26	2 (120)	-3.53 (-5.28 to -1.78) ***	0
		Unsupervised	1 (11)	-4.50 (-12.93 to 3.93)	NA	1 (11)	-5.70 (-20.72 to 9.32)	NA
		Mixed Delivery	1 (25)	0.15 (-2.50 to 2.80)	NA	1 (25)	0.30 (-5.72 to 6.32)	NA
BMI (kg/m^2)	BMI at entry	≤ 24.9 kg/m^2	1 (30)	-0.14 (-1.23 to 0.95)	NA	1 (30)	-0.00 (-3.05 to 3.05)	0
		25-29.9 kg/m^2	6 (222)	-0.01 (-0.47 to 0.45)	30	6 (188)	-1.04 (-1.89 to -0.19) *	0
		≥ 30 kg/m^2	4 (79)	-1.34 (-1.86 to -0.82) ***	0	3 (54)	-0.70 (-5.55 to 4.15)	38
	Intervention type	Aerobic exercise	8 (260)	-0.78 (-1.38 to -0.18) **	57	7 (201)	-1.45 (-2.59 to -0.32) **	0
		Resistance exercise	3 (50)	0.50 (0.00 to 1.00) *	0	3 (50)	-0.10 (-2.76 to 2.55)	23
		Combined exercise	1 (30)	-0.14 (-1.23 to 0.95)	NA	1 (30)	0.00 (-3.05 to 3.05)	NA
	Intervention duration	≤12 weeks	8 (245)	-0.43 (-1.22 to 0.35)	64	7 (220)	-0.91 (-1.73 to -0.08) *	0
		>12 weeks	3 (86)	-0.61 (-1.61 to 0.38)	77	3 (52)	-2.42 (-5.28 to 0.45)	0
	Delivery	Supervised	8 (248)	-0.65 (-1.42 to 0.12)	74	7 (223)	-1.06 (-1.87 to -0.25) **	0

	format	Unsupervised	1 (45)	-0.10 (-0.51 to 0.31)	NA	1 (11)	-2.10 (-8.60 to 4.40)	NA
		Mixed Delivery	2 (38)	0.19 (-1.56 to 1.93)	0	2 (38)	1.82 (-5.85 to 9.50)	45
Body Mass (kg)	BMI at entry	≤ 24.9 kg/m^2	1 (20)	0.60 (-2.07 to 3.27)	NA	1 (20)	-1.60 (-7.40 to 4.20)	NA
		25-29.9 kg/m^2	3 (67)	-0.40 (-3.02 to 2.21)	0	3 (67)	-0.55 (-7.88 to 6.78)	0
		≥ 30 kg/m^2	3 (52)	-4.07 (-6.46 to -1.67) ***	0	2 (27)	13.88 (-16.21 to 43.97)	61
	Intervention type	Aerobic exercise	5 (98)	-1.88 (-4.08 to 0.32)	38	4 (73)	-1.54 (-8.26 to 5.17)	0
		Resistance exercise	3 (50)	0.62 (-1.27 to 2.51)	0	3 (50)	3.99 (-9.39 to 17.36)	50
	Intervention duration	≤12 weeks	6 (125)	-1.23 (-3.45 to 0.98)	44	5 (100)	-0.59 (-5.10 to 3.92)	0
		>12 weeks	1 (14)	-0.70 (-10.81 to 9.41)	NA	1 (14)	1.49 (-24.92 to 27.90)	NA
	Delivery format	Supervised	5 (101)	-1.61 (-4.21 to 0.99)	49	4 (76)	-1.00 (-5.72 to 3.72)	0
		Mixed Delivery	2 (38)	0.26 (-3.22 to 3.74)	0	2 (38)	11.85 (-21.86 to 45.56)	71
Waist Circumference (cm)	BMI at entry	≤ 24.9 kg/m^2	1 (30)	-1.40 (-2.21 to -0.59)	NA	1 (30)	-1.00 (-9.68 to 7.68)	NA
		25-29.9 kg/m^2	3 (137)	-2.21 (-4.25 to -0.16) *	0	3 (137)	-2.02 (-3.39 to -0.65) **	0
		≥ 30 kg/m^2	3 (54)	-4.18 (-7.86 to -0.50) *	62	3 (54)	1.38 (-14.27 to 17.04)	69
	Intervention type	Aerobic exercise	5 (170)	-3.30 (-6.10 to -0.51) *	50	5 (170)	-2.22 (-3.56 to -0.87) ***	0
		Resistance exercise	2 (30)	-2.40 (-4.04 to -0.75) **	0	2 (30)	10.31 (-13.73 to 34.35)	62
		Combined exercise	1 (30)	-1.40 (-2.21 to -0.59)	NA	1 (30)	-1.00 (-9.68 to 7.68)	NA
	Intervention duration	≤12 weeks	5 (180)	-1.69 (-2.38 to -0.99) ***	0	5 (180)	-1.73 (-4.25 to 0.78)	8
		>12 weeks	2 (41)	-5.19 (-11.43 to 1.05)	52	2 (41)	-6.86 (-14.02 to 0.30)	0
	Delivery format	Supervised	5 (183)	-3.21 (-5.56 to -0.85) **	64	5 (183)	-2.16 (-3.50 to -0.82) **	0
		Mixed Delivery	2 (38)	-2.09 (-4.36 to 0.19)	28	2 (38)	8.80 (-17.70 to 35.29)	72
Body Fat (%)	BMI at entry	25-29.9 kg/m^2	2 (47)	-1.60 (-3.68 to 0.47)	59	2 (47)	-4.51 (-8.10 to -0.92) **	0
		≥ 30 kg/m^2	1 (13)	-1.00 (-2.56 to 0.56)	NA	1 (13)	3.50 (-7.08 to 14.08)	NA
	Intervention type	Aerobic exercise	2 (39)	-1.36 (-3.73 to 1.01)	76	2 (39)	-4.99 (-8.73 to -1.25) **	0
		Resistance exercise	2 (30)	-0.95 (-2.02 to 0.13)	0	2 (30)	0.47 (-5.95 to 6.88)	0
	Delivery format	Supervised	1 (22)	-2.62 (-4.39 to -0.85)	NA	1 (22)	-5.48 (-9.83 to -1.13)	NA
		Mixed Delivery	2 (38)	-0.81 (-2.03 to 0.42)	0	2 (38)	-0.88 (-6.32 to 4.56)	0

Outcome: outcome where sub-analysis was completed. Sub-analysis: how the studies were categorised for analysis. Sub-group: groups each study was classified into. Trials: number of studies included within sub-analysis, *N:* number or participants included within sub-analysis. Effect estimates are reported as mean differences, and 95% confidence intervals, between exercise and control groups. ▲: positive values favour exercise over control. Significant evidence of effect denoted by: * $P \leq 0.05$; ** $P \leq 0.01$; *** $P \leq 0.001$. Heterogeneity reported using I^2 statistic: 0-40% might not be important; 30-60% may represent moderate heterogeneity; 50-90% may represent substantial heterogeneity; 75-100% may represent considerable heterogeneity. NA: I^2 not applicable. VO_2 max/peak: maximum or peak relative volume of oxygen consumed; BMI: body mass index; LDL-C: low-density lipoprotein cholesterol; HDL-C: high-density lipoprotein cholesterol.

Supplementary Table 8. Summary of effect estimates and heterogeneity from sub-group analyses in androgenic and inflammatory outcomes.

Outcome	Sub-analysis	Sub-group	Change from baseline			Post-intervention		
			Trials (*N*)	Effect Estimate MD (95% CI)	I^2 (%)	Trials (*N*)	Effect Estimate MD (95% CI)	I^2 (%)
Total Testosterone (nmol/L)	BMI at entry	≤ 24.9 kg/m^2	1 (30)	-0.10 (-2.04 to 1.84)	NA	1 (30)	-0.10 (-1.91 to 1.71)	NA
		25-29.9 kg/m^2	3 (160)	-0.11 (-0.27 to 0.05)	0	3 (126)	-0.03 (-0.46 to 0.41)	68
		≥ 30 kg/m^2	1 (13)	0.10 (-0.33 to 0.53)	NA	1 (13)	-0.10 (-0.54 to 0.34)	NA
	Intervention type	Aerobic exercise	3 (152)	-0.10 (-0.27 to 0.06)	0	3 (118)	-0.03 (-0.48 to 0.43)	60
		Resistance exercise	2 (30)	-0.00 (-0.30 to 0.30)	0	2 (30)	0.04 (-0.29 to 0.36)	0
		Combined exercise	1 (30)	-0.10 (-2.04 to 1.84)	NA	1 (30)	-0.10 (-1.91 to 1.71)	NA
	Intervention duration	≤12 weeks	4 (158)	-0.02 (-0.23 to 0.19)	0	4 (158)	-0.08 (-0.40 to 0.25)	50
		>12 weeks	1 (45)	-0.16 (-0.37 to 0.06)	NA	1 (11)	0.00 (-0.62 to 0.62)	NA
	Delivery format	Supervised	2 (120)	-0.10 (-0.41 to 0.21)	0	2 (120)	-0.30 (-0.51 to -0.09)	0
		Unsupervised	1 (45)	-0.16 (-0.37 to 0.06)	NA	1 (11)	0.00 (-0.62 to 0.62)	NA
		Mixed Delivery	2 (38)	0.04 (-0.24 to 0.32)	0	2 (38)	0.11 (-0.33 to 0.55)	45
SHBG (nmol/L)	BMI at entry	25-29.9 kg/m^2	3 (160)	11.16 (-8.39 to 30.71)	92	3 (126)	14.99 (-18.49 to 48.47)	0
		≥ 30 kg/m^2	1 (13)	-5.00 (-20.66 to 10.66)	NA	1 (13)	-20.00 (-45.80 to 5.80)	NA
	Intervention type	Aerobic exercise	3 (152)	9.49 (-13.77 to 32.76)	92	3 (118)	18.97 (-23.25 to 61.19)	69
		Resistance exercise	2 (30)	4.98 (-14.52 to 24.49)	68	2 (30)	-0.79 (-45.26 to 43.67)	67
	Intervention duration	≤12 weeks	3 (128)	2.45 (-1.04 to 5.93)	0	3 (128)	-0.81 (-8.06 to 6.45)	22
		>12 weeks	1 (45)	30.31 (20.15 to 40.47)	NA	1 (11)	-3.10 (-57.82 to 51.62)	NA
	Delivery format	Supervised	1 (90)	3.00 (-0.65 to 6.65)	NA	1 (90)	1.00 (-1.63 to 3.63)	NA
		Unsupervised	1 (45)	30.31 (20.15 to 40.47)	NA	1 (11)	-3.10 (-57.82 to 51.62)	NA
		Mixed Delivery	2 (38)	-3.23 (-14.91 to 8.46)	0	2 (38)	4.51 (-56.46 to 85.47)	89
Free Androgen Index	BMI at entry	25-29.9 kg/m^2	3 (126)	0.09 (-0.78 to 0.96)	0	3 (126)	0.06 (-1.13 to 1.26)	10
		≥ 30 kg/m^2	1 (13)	1.00 (-0.91 to 2.91)	NA	1 (13)	5.10 (-0.25 to 10.45)	NA
	Intervention type	Aerobic exercise	3 (118)	0.51 (-0.52 to 1.53)	0	3 (118)	0.10 (-1.17 to 1.36)	10
		Resistance exercise	2 (30)	-0.04 (-1.67 to 1.58)	57	2 (30)	1.71 (-3.65 to 7.08)	74
	Intervention duration	≤12 weeks	3 (128)	0.11 (-0.71 to 0.93)	0	3 (128)	0.34 (-1.45 to 2.13)	50
		>12 weeks	1 (11)	2.10 (-0.92 to 5.12)	NA	1 (11)	3.40 (-1.67 to 8.47)	NA
	Delivery format	Supervised	1 (90)	0.20 (-1.23 to 1.63)	NA	1 (90)	-0.20 (-1.23 to 1.63)	NA
		Unsupervised	1 (11)	2.10 (-0.92 to 5.12)	NA	1 (11)	3.40 (-1.67 to 8.47)	NA
		Mixed Delivery	2 (38)	1.79 (-3.18 to 6.76)	72	2 (38)	1.67 (-3.80 to 7.14)	75

Oestradiol (pmol/L)	BMI at entry	≤ 24.9 kg/m^2	1 (30)	27.28 (-8.98 to 63.54)	NA	1 (30)	6.70 (-21.73 to 35.13)	NA
		25-29.9 kg/m^2	2 (135)	-41.14 (-141.68 to 59.40)	70	1 (90)	-1.00 (-13.62 to 11.62)	NA
		≥ 30 kg/m^2	1 (25)	-77.09 (-166.91 to 12.73)	NA	-	-	-
	Intervention type	Aerobic exercise	3 (160)	-47.22 (-117.52 to 23.08)	65	1 (90)	-1.00 (-13.62 to 11.62)	NA
		Combined exercise	1 (30)	27.28 (-8.98 to 63.54)	NA	1 (30)	6.70 (-21.73 to 35.13)	NA
	Intervention duration	≤12 weeks	3 (145)	-1.14 (-36.61 to 34.33)	61	2 (120)	0.27 (-11.27 to 11.80)	0
		>12 weeks	1 (45)	-110.13 (-224.10 to 3.84)	NA	-	-	-
	Delivery format	Supervised	3 (145)	-1.14 (-36.61 to 34.33)	61	2 (120)	0.27 (-11.27 to 11.80)	0
		Unsupervised	1 (45)	-110.13 (-224.10 to 3.84)	NA	-	-	-
Luteinising Hormone (IU/L)	BMI at entry	≤ 24.9 kg/m^2	1 (30)	1.60 (-0.17 to 3.37)	NA	1 (30)	-3.60 (-7.47 to 0.27)	NA
		25-29.9 kg/m^2	3 (155)	-1.12 (-3.63 to 1.39)	61	3 (121)	0.15 (-0.92 to 1.22)	0
	Intervention type	Aerobic exercise	3 (155)	-1.12 (-3.63 to 1.39)	61	3 (121)	0.15 (-0.92 to 1.22)	0
		Combined exercise	1 (30)	1.60 (-0.17 to 3.37)	NA	1 (30)	-3.60 (-7.47 to 0.27)	NA
	Intervention duration	≤12 weeks	3 (140)	-0.59 (-3.24 to 2.06)	81	3 (140)	-1.60 (-4.73 to 1.54)	62
		>12 weeks	1 (45)	1.18 (-3.15 to 5.51)	NA	1 (11)	0.00 (-2.20 to 2.20)	NA
	Delivery format	Supervised	3 (140)	-0.59 (-3.24 to 2.06)	81	3 (140)	-1.60 (-4.73 to 1.54)	62
		Unsupervised	1 (45)	1.18 (-3.15 to 5.51)	NA	1 (11)	0.00 (-2.20 to 2.20)	NA
Follicle Stimulating Hormone (IU/L)	BMI at entry	≤ 24.9 kg/m^2	1 (30)	0.36 (-0.06 to 0.78)	NA	1 (30)	-0.30 (-1.01 to 0.41)	NA
		25-29.9 kg/m^2	3 (155)	0.09 (-0.35 to 0.52)	0	3 (121)	0.11 (-0.35 to 0.56)	0
	Intervention type	Aerobic exercise	3 (155)	0.09 (-0.35 to 0.52)	0	3 (121)	0.11 (-0.35 to 0.56)	0
		Combined exercise	1 (30)	0.36 (-0.06 to 0.78)	NA	1 (30)	-0.30 (-1.01 to 0.41)	NA
	Intervention duration	≤12 weeks	3 (140)	0.19 (-0.13 to 0.51)	0	3 (140)	-0.03 (-0.42 to 0.37)	0
		>12 weeks	1 (45)	0.57 (-0.44 to 1.58)	NA	1 (11)	0.20 (-1.34 to .74)	NA
	Delivery format	Supervised	3 (140)	0.19 (-0.13 to 0.51)	0	3 (140)	-0.03 (-0.42 to 0.37)	0
		Unsupervised	1 (45)	0.57 (-0.44 to 1.58)	NA	1 (11)	0.20 (-1.34 to .74)	NA

Outcome: outcome where sub-analysis was completed. Sub-analysis: how the studies were categorised for analysis. Sub-group: groups each study was classified into. Trials: number of studies included within sub-analysis, *N*: number or participants included within sub-analysis. Effect estimates are reported as mean differences (MD), and 95% confidence intervals (CI), between exercise and control groups. Heterogeneity reported using I^2 statistic: 0-40% might not be important; 30-60% may represent moderate heterogeneity; 50-90% may represent substantial heterogeneity; 75-100% may represent considerable heterogeneity. NA: I^2 not applicable. BMI: body mass index; SHBG: sex hormone-binding globulin.

Supplementary Table 9. Exercise versus Control: Summary of findings from investigative outcomes that were only reported in single trials.

Trial	Significance	Outcomes
**Almenning et al. [32]**	No statistically significant findings	HR recovery; Leptin
	Statistically significant findings	• Nitric oxide bio-availability [as measured by flow mediated dilation (FMD) %] - reported a statistically significant improvement in FMD following a HIT intervention, but not resistance training.<br>• Homocysteine – significant change from baseline concentrations (MD: -0.6 µmol/L, 95% CI: -1.0 to -0.1) following a HIT intervention but no between group differences.
**Sa et al. [107]**	No statistically significant findings	Area under the curve (AUC) oral glucose tolerance test; two-hour post-prandial blood glucose; interleukin-6; tumour necrosis-α
	Statistically significant findings	• Mean arterial blood pressure; a statistical reduction in the exercise group (MD: -6.8 mmHg, 95% CI: -10.6 to -3.0; 14 participants) and a significant time by group interaction, representative of a moderate effect size (*d*) was also reported (*d:* -1.0; 95% CI: -1.7 to -0.3)
**Stener-Victorin et al. [111]**	No statistically significant findings	Number of participants with acne; menstrual frequency; 5α-dihydrotestosterone; estrone; DHEA; androstenedione; 5-androstene-3β, 17β-diol, androsterone glucuronide; androstane-3α, 17β-diol-3 glucuronide; 17β-diol-17 glucuronide; insulin growth factor-1; thyroid stimulating hormone; free thyroxin 4; fibrinogen; fibrin D-dimer; von Willebrand factor; factor VIII; tissue plasminogen activator and plasminogen activator inhibitor; ovarian volume; Montgomery Asberg Depression

		Rating Scale (MADRS) and the Brief Scale for Anxiety (BSA)
	Statistically significant findings	• Estrone sulfate (E1-S) - significantly lower (*P* <.05) in the exercise group versus control when measured immediately post-intervention; this effect disappeared during follow-up assessment.<br>• Median antral follicle counts - were significantly lower (-11.7%; *P*= .010) from baseline to follow-up in the exercise group.
**Turan [117]**	Statistically significant findings	• Respiratory rate – significant within exercise group changes in respiratory rate (-1.0 ± 0.4 breaths/minute) following exercise, but not between differences of groups.<br>• Hip circumference - a statistically significant (P <.05) reduction following exercise training, and a statistical difference (P <.05) between change scores in each arm
**Vigorito et al. [118]**	No statistically significant findings	Respiratory exchange ratio; Peak HR
	Statistically significant findings	• AUC-glucose: AUC-insulin ratio - a statistically significant change from baseline for AUC-insulin and the ratio with AUC-glucose in the exercise group but not in the control.<br>• AUC-insulin - significantly improved (*P* <.001) compared to the control group.<br>• VO_2 at anaerobic threshold - within and between group statistical changes for VO_2 at anaerobic threshold (MD: 4.4 ml/kg/min^{-}1; *P* <.001)<br>• Maximum workload - within and between group statistical changes (MD: 32.3 Watts; *P* <.001)<br>• Ventilatory equivalent for carbon dioxide production - only within group changes

		were reported (VE/VCO_2: MD: -0.6; *P*= .01).<br>• Participant leisure time physical activity (METs-hrs/wk) - significantly higher (*P* <.001) following an exercise intervention.
**Vizza et al. [119]**	Statistically significant findings	• Glycated haemoglobin (HbA1c) - Resistance training statistically reduced (*P*= .037) within group HbA1c percentage, and when compared with control (*P*= .03, *d*= 0.39).<br>• Lower, but not upper, body strength was significantly increased (*P*= .04) following a resistance training intervention; it was also significantly improved compared to a control (ES: 0.45; *P*= .03).<br>• Depression, Anxiety and Stress Scale 21 (DASS-21) - the depression domain showed within group (*P*= 0.050) and between group (ES: 0.50; *P*= 0.01) reductions following resistance training.<br>• Exercise Self Efficacy Scale - a statistically significant reduction (*P*= 0.04) of self-efficacy within the control group, but no changes in the exercise groups or differences between groups.

HR: heart rate; FMD: flow mediated dilation; HIT: high-intensity interval training; MD: mean difference; DHEA: dehydroepiandrosterone; AUC: area under the curve; VO_2: volume of oxygen; VE/VCO_2: minute ventilation/carbon dioxide production; MET: metabolic equivalent of task.

Supplementary Table 10. Exercise and Diet versus Control: Summary of findings from investigative outcomes that were only reported in single trials.

Trial	Significance	Outcomes
**Guzick et al. [98]**	No statistically significant findings	Fasting insulin; luteinising hormone; follicle stimulating hormone.
	Statistically significant findings	• Bodyweight - statistical interaction effect (*P* <.0001) reflecting an improvement following combined exercise and diet intervention, but not control.<br>• Free testosterone - statistical interaction effect (*P*=.02) following a combined exercise and diet intervention, but not control.
**Hoeger et al. [99]**	No statistically significant findings	Free androgen index; AUC-glucose; AUC-insulin; fasting blood glucose; ovulatory status
	Statistically significant findings	• Bodyweight - statistically significant (*P* <.05) within-group bodyweight reductions for lifestyle and placebo, but no statistical differences versus placebo alone.<br>• When lifestyle was combined with Metformin, statistical differences (*P* <.05) compared to placebo only were reported for body weight, SHBG and FAI.
**Petranyi et al. [106]**	Statistically significant findings	• Statistically significant (*P* <.001) reductions in levels of acne, FG scores and BMI following lifestyle and Metformin therapy; changes in the Metformin only arm were comparable apart from BMI-related which was statistically higher in the combined treatment (*P*= .03).

AUC: area under the curve; SHBG: sex hormone binding globulin; FAI: free androgen index; FG: Ferriman-Gallwey; BMI: body mass index

Supplementary Table 11. Effect estimates and heterogeneity for change from baseline to immediately post-intervention, and immediately post-intervention values only, for all outcomes analysed in the comparison Exercise and Diet versus Diet Only.

	Change from baseline						Immediately post-intervention					
Outcome	Trials	*N*	MD	95% CI		I^2 (%)	Trials	*N*	MD	95% CI		I^2 (%)
				Lower	Upper					Lower	Upper	
FBG (mg/dL)	2	78	2.92	-0.40	6.23	42	2	78	2.86	-1.56	7.29	0
FI (μIU/mL)	3	90	2.22	-3.70	8.14	62	2	64	-2.72	-7.70	2.27	0
HOMA-IR	2	78	-0.01	-0.45	0.43	0	-	-	-	-	-	-
Body Weight (kg)	2	64	-0.40	-3.64	2.83	0	2	64	1.49	-8.05	11.03	0
BMI (kg/m^2)	2	38	-0.09	-1.27	1.09	0	2	38	2.56	-1.77	6.88	0
WC (cm)	2	64	-0.47	-3.95	3.01	0	2	64	-1.51	-8.69	5.67	0
Body Fat (%)	2	78	-1.05	-4.61	2.50	85	2	78	-0.93	-3.63	1.77	10
FFM (kg) ▲	2	78	0.40	-3.24	4.03	85	2	78	2.07	-1.72	5.86	0
Testosterone (nmol/L)	3	90	0.29	-0.49	1.08	78	3	90	0.08	-0.38	0.54	0
SHBG (nmol/L) ▲	3	90	2.18	-3.15	7.51	51	3	90	6.45	-5.52	18.42	61
FAI	2	64	0.11	-2.28	2.50	0	2	64	-2.88	-6.58	0.81	0

Negative values favour exercise and diet combined except where stated otherwise. ▲: positive values favour exercise and diet combined over diet only. Trials: number of studies included within analysis, *N:* number or participants included within analysis. Effect estimates are reported as mean differences (MD), and 95% confidence intervals (CI), between exercise and diet combined vs diet only groups. Heterogeneity reported using I^2 statistic. FBG: fasting blood glucose; FI: fasting insulin; HOMA-IR: homeostatic model of assessment, insulin resistance; BMI: body mass index; WC: waist circumference; FFM: fat free mass; SHBG: sex hormone binding globulin; FAI: free androgen index.

Supplementary Table 12. Exercise and Diet versus Diet: Summary of findings from investigative outcomes that were only reported in single trials.

Trial	Significance	Outcomes
**Bruner et al. [97]**	No statistically significant findings	Resting energy expenditure; LH/FSH ratio; number of ovarian follicles (left and right)
	Statistically significant findings	• Sum of two skinfolds (subscapular and iliac crest) - statistically lower than at baseline and a group x time interaction (*P*= .002) immediately post-intervention with a greater decrease in the exercise and diet group compared with diet only.
**Nybacka et al. [104]**	No statistically significant findings	• No significant changes seen in any intervention arm for ratio of upper/lower body fat. No effect seen in upper body fat (kg) for diet only or diet and exercise combined; no reduction in lower body fat for the exercise only arm.<br>• Exercise and diet combined did not significantly reduce IGF-I or IGFBP-1
	Statistically significant findings	• In the diet only arm statistical changes in free testosterone (-3.66 pg/mL, 95% CI: -6.12 to -1.20; *P* <.001), AMH (*P* <.01), IGF-1 (17.1 µg/L, 95% CI: 0.3 to 33.9; *P* <.05), and IGFBP-1 (0.32 µg/L, 95% CI: 0.01 to 0.64; *P* <.05) were reported that were not present in the combined arm.<br>• There were statistically significant reductions in lower body fat for diet only (-1055g, 95% CI: -1787 to -322; *P* <.01) and diet and exercise (1616g, 95% CI: -2407 to -825; *P* <.001), lean body mass in the diet and exercise arm only (-2.66kg, 95% CI: -4.14 to -1.18; *P* <.001), mean ovarian follicle number in both diet only (*P* <.05) and the combined arm (*P* <.05), as well as improvements to ovulatory function in both

		intervention arms (diet: *P* <.001; combined: *P* <.05).<br>• Mean ovarian volume was reduced in the diet and exercise arm only (*P* <.05).
**Thomson et al. [33]**	No statistically significant findings	The Centre of Epidemiologic Studies Depression Scale was also used but there were no differences in post-intervention scores compared to baseline.
	Statistically significant findings	• Statistically significant reductions (*P* ≤.03) to fat mass and abdominal fat mass in all groups; both exercise arms were also statistically different (P ≤.03) to the diet only arm.<br>• Levels of endothelial function were also measured; vascular cell adhesion molecule-I (*P*= .01), plasminogen activator inhibitor-I (*P* <.001) and intra-cellular adhesion molecule-I (*P* <.001) were reduced in all treatment arms with no statistical differences between treatments.<br>• PCOS-Q was used to assess quality of life; and found statistical improvements (*P* ≤.001) across all treatment arms in each domain apart from body hair scores. No differences between treatment arms were found.

LH/FSH: luteinising hormone/follicle stimulating hormone; IGF-1: insulin-like growth factor-1; IGFBP-1; insulin-like growth factor binding protein-1; AMH: anti-Müllerian hormone; PCOS-Q; polycystic ovary syndrome questionnaire.
