# Supplementary material for: Exercise, or exercise and diet for the management of polycystic ovary syndrome: a systematic review and meta-analysis
Source: Syst Rev. 2019 Feb 12;8:51. doi: 10.1186/s13643-019-0962-3 (PMC6371542; doi:10.1186/s13643-019-0962-3)
Supplement: Supplementary file 2 — Figure S1. Review of authors’ judgements about each risk of bias item for each included study; Figure S2. Forest plot of comparison: exercise vs. control. Analysis of immediately post-intervention values for outcomes related to participant lipid profile; Figure S3. Forest plot of comparison: exercise vs. control. Analysis of resting heart rate subgroups by intervention type; Figure S4. Forest plot of comparison: exercise vs. control, change from baseline; outcome: SF-36 domains; Figure S5. Forest plot of comparison: exercise and diet vs. control; outcome: waist-to-hip ratio; Figure S6. Forest plot of comparison: exercise and diet vs. control, change from baseline. outcome: SHBG. (PDF 345 kb) [file 13643_2019_962_MOESM2_ESM.pdf]

Supplementary Figure 1. Review of authors' judgements about each risk of bias item for each included study.

	Random sequence generation (selection bias)	Allocation concealment (selection bias)	Blinding of participants and personnel (performance bias)	Blinding of outcome assessment (detection bias)	Incomplete outcome data (attrition bias)	Selective reporting (reporting bias)	Group similarity at baseline (other bias)	Adherence (other bias)	Contamination (other bias)
Almenning 2015	+	+	–	–	+	+	+	+	?
Brown 2009	+	+	–	–	–	?	–	+	?
Bruner 2006	?	+	–	–	–	?	+	?	?
Guzick 1994	?	?	–	–	+	?	+	?	?
Hoeger 2004	+	?	–	–	–	?	+	?	?
Konopka 2015	?	?	–	–	–	+	+	?	?
Nasrekani 2016	?	?	–	–	+	?	+	?	?
Nybacka 2011	+	?	–	–	–	?	+	?	?
Petranyi 2011	?	?	–	–	+	?	?	?	?
Roessler 2013	?	?	–	–	+	?	+	–	–
Sa 2015	–	–	–	–	+	–	+	?	–
Saremi 2013	?	?	–	–	+	?	?	?	?
Saremi 2016	?	+	–	–	?	?	?	?	?
Stener-Victorin 2009	+	?	–	–	–	+	+	+	–
Thomson 2008	+	?	–	–	–	?	+	?	?
Turan 2015	+	+	–	–	+	?	+	+	?
Vigorito 2007	?	?	–	+	+	?	?	+	+
Vizza 2016	+	+	–	–	–	?	–	–	?

Supplementary Figure 2. Forest plot of comparison: Exercise vs. Control. Analysis of immediately post-intervention values for outcomes related to participant lipid profile.

Study or Subgroup	Exercise Mean [mg/dL]	Exercise SD [mg/dL]	Exercise Total	Control Mean [mg/dL]	Control SD [mg/dL]	Control Total	Weight	Mean Difference IV, Random, 95% CI [mg/dL]	A	B	C	D	E	F	G	H	I
**1.11.4 Triglycerides (post-intervention)**																	
Saremi 2016	111.8	45.7	10	106.7	28.6	10	2.5%	5.10 [-28.31, 38.51]	?	+	-	-	?	?	?	?	?
Saremi 2013	100.8	47.11	11	102.2	16.7	11	3.2%	-1.40 [-30.94, 28.14]	?	?	-	-	+	?	?	?	?
Stener-Victorin 2009	61.95	8.85	5	70.8	35.4	6	3.2%	-8.85 [-38.22, 20.52]	+	?	-	-	-	+	+	+	-
Almenning 2015	84.075	48.04	16	63.72	17.7	9	4.0%	20.36 [-5.87, 46.58]	+	+	-	-	+	+	+	+	?
Sa 2015	17.48	6.85	14	32.43	24.5	13	12.7%	-14.95 [-28.74, -1.16]	-	-	-	-	+	-	+	?	-
Vigorito 2007	113.3	23.3	45	111	23	45	22.2%	2.30 [-7.27, 11.87]	?	?	-	+	+	?	?	+	+
Turan 2015	138.2	2.9933	14	140.5	7.6	16	52.2%	-2.30 [-6.34, 1.74]	+	+	-	-	+	?	+	+	?
**Subtotal (95% CI)**			**115**			**110**	**100.0%**	**-1.97 [-7.36, 3.42]**									
Heterogeneity: Tau² = 10.22; Chi² = 7.36, df = 6 (P = 0.29); I² = 18%																	
Test for overall effect: Z = 0.72 (P = 0.47)																	
**1.11.5 Total cholesterol (post-intervention)**																	
Saremi 2016	111.8	45.7	10	106.7	28.6	10	1.7%	5.10 [-28.31, 38.51]	?	+	-	-	?	?	?	?	?
Almenning 2015	175.95	21.27	16	170.15	46.4	9	1.9%	5.80 [-26.26, 37.86]	+	+	-	-	+	+	+	+	?
Stener-Victorin 2009	154.7	19.33	5	154.7	27.07	6	2.6%	0.00 [-27.50, 27.50]	+	?	-	-	-	+	+	+	-
Saremi 2013	162.4	27.03	11	169.2	23.92	11	4.3%	-6.80 [-28.13, 14.53]	?	?	-	-	+	?	?	?	?
Turan 2015	195.1	11.225	14	200.7	16	16	20.2%	-5.60 [-15.40, 4.20]	+	+	-	-	+	?	+	+	?
Sa 2015	64.51	9.73	14	75.32	11.89	13	28.6%	-10.81 [-19.04, -2.58]	-	-	-	-	+	-	+	?	-
Vigorito 2007	151	16.4	45	156	17	45	40.7%	-5.00 [-11.90, 1.90]	?	?	-	+	+	?	?	+	+
**Subtotal (95% CI)**			**115**			**110**	**100.0%**	**-6.35 [-10.76, -1.95]**									
Heterogeneity: Tau² = 0.00; Chi² = 2.51, df = 6 (P = 0.87); I² = 0%																	
Test for overall effect: Z = 2.83 (P = 0.005)																	
**1.11.6 LDL-C (post-intervention)**																	
Almenning 2015	90.87	24.36	16	96.67	46.4	9	2.3%	-5.80 [-38.38, 26.78]	+	+	-	-	+	+	+	+	?
Stener-Victorin 2009	85.07	23.2	5	85.07	19.33	6	3.8%	0.00 [-25.55, 25.55]	+	?	-	-	-	+	+	+	-
Saremi 2013	99.8	27.54	11	118	14.62	11	7.3%	-18.20 [-36.63, 0.23]	?	?	-	-	+	?	?	?	?
Saremi 2016	94.7	20.6	10	117.5	21.2	10	7.4%	-22.80 [-41.12, -4.48]	?	+	-	-	?	?	?	?	?
Turan 2015	114	18.3341	14	118.6	12.8	16	18.9%	-4.60 [-16.07, 6.87]	+	+	-	-	+	?	+	+	?
Vigorito 2007	73.2	23.8	45	75.9	24.9	45	24.5%	-2.70 [-12.76, 7.36]	?	?	-	+	+	?	?	+	+
Sa 2015	39.28	10.45	14	44.87	11.53	13	35.8%	-5.59 [-13.91, 2.73]	-	-	-	-	+	-	+	?	-
**Subtotal (95% CI)**			**115**			**110**	**100.0%**	**-6.68 [-11.66, -1.70]**									
Heterogeneity: Tau² = 0.00; Chi² = 5.53, df = 6 (P = 0.48); I² = 0%																	
Test for overall effect: Z = 2.63 (P = 0.009)																	

Mean Difference IV, Random, 95% CI [mg/dL]

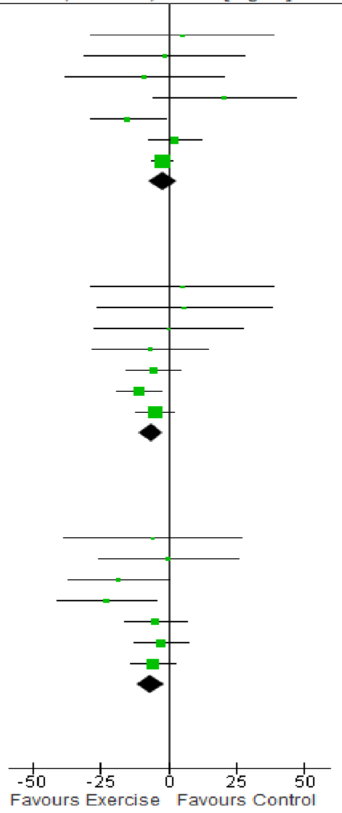

Test for subgroup differences: Chi² = 1.98, df = 2 (P = 0.37), I² = 0%

Risk of bias legend
(A) Random sequence generation (selection bias)
(B) Allocation concealment (selection bias)
(C) Blinding of participants and personnel (performance bias)
(D) Blinding of outcome assessment (detection bias)
(E) Incomplete outcome data (attrition bias)
(F) Selective reporting (reporting bias)
(G) Group similarity at baseline (other bias)
(H) Adherence (other bias)
(I) Contamination (other bias)

Supplementary Figure 3 - Forest plot of comparison: Exercise vs. Control. Analysis of resting heart rate sub-groups by intervention type.

	Exercise			Control				Mean Difference	Risk of Bias								
**Study or Subgroup**	**Mean [BPM]**	**SD [BPM]**	**Total**	**Mean [BPM]**	**SD [BPM]**	**Total**	**Weight**	**IV, Random, 95% CI [BPM]**	**A**	**B**	**C**	**D**	**E**	**F**	**G**	**H**	**I**
**8.3.1 Aerobic Exercise (change from baseline)**																	
Stener-Victorin 2009	-4.2	5.69	5	0.3	8.49	6	6.7%	-4.50 [-12.93, 3.93]	+	?	-	-	-	+	+	+	-
Almenning 2015	-4.6	5.9807	8	-0.9	3.2524	9	21.9%	-3.70 [-8.36, 0.96]	+	+	-	-	+	+	+	+	?
Vigorito 2007	-2.2	6.5803	45	0.9	5.8782	45	71.4%	-3.10 [-5.68, -0.52]	?	?	-	+	+	?	?	+	?
**Subtotal (95% CI)**			**58**			**60**	**100.0%**	**-3.32 [-5.50, -1.15]**									
Heterogeneity: Tau² = 0.00; Chi² = 0.13, df = 2 (P = 0.94); I² = 0%																	
Test for overall effect: Z = 2.99 (P = 0.003)																	
**8.3.2 Resistance Exercise (change from baseline)**																	
Almenning 2015	2.6	5.7415	8	-0.9	3.2524	9	100.0%	3.50 [-1.01, 8.01]	+	+	-	-	+	+	+	+	?
**Subtotal (95% CI)**			**8**			**9**	**100.0%**	**3.50 [-1.01, 8.01]**									
Heterogeneity: Not applicable																	
Test for overall effect: Z = 1.52 (P = 0.13)																	
**8.3.3 Aerobic and Resistance Exercise (change from baseline)**																	
Turan 2015	-3.62	10.1025	14	3.5	6.8	16	100.0%	-7.12 [-13.37, -0.87]	+	+	-	-	+	?	+	+	?
**Subtotal (95% CI)**			**14**			**16**	**100.0%**	**-7.12 [-13.37, -0.87]**									
Heterogeneity: Not applicable																	
Test for overall effect: Z = 2.23 (P = 0.03)																	
**8.3.4 Aerobic Exercise (post-intervention)**																	
Stener-Victorin 2009	63.6	9.1	5	69.3	15.9	6	1.3%	-5.70 [-20.72, 9.32]	+	?	-	-	-	+	+	+	-
Almenning 2015	59.1	6.3	8	58.4	6.2	9	8.3%	0.70 [-5.26, 6.66]	+	+	-	-	+	+	+	+	?
Vigorito 2007	74.8	4.8	45	78.1	3.9	45	90.4%	-3.30 [-5.11, -1.49]	?	?	-	+	+	?	?	+	?
**Subtotal (95% CI)**			**58**			**60**	**100.0%**	**-3.00 [-4.72, -1.28]**									
Heterogeneity: Tau² = 0.00; Chi² = 1.71, df = 2 (P = 0.42); I² = 0%																	
Test for overall effect: Z = 3.42 (P = 0.0006)																	
**8.3.5 Resistance Exercise (post-intervention)**																	
Almenning 2015	58.3	11.7	8	58.4	6.2	9	100.0%	-0.10 [-9.16, 8.96]	+	+	-	-	+	+	+	+	?
**Subtotal (95% CI)**			**8**			**9**	**100.0%**	**-0.10 [-9.16, 8.96]**									
Heterogeneity: Not applicable																	
Test for overall effect: Z = 0.02 (P = 0.98)																	
**8.3.6 Aerobic and Resistance Exercise (post-intervention)**																	
Turan 2015	81.2	8.2316	14	88.1	11.2	16	100.0%	-6.90 [-13.88, 0.08]	+	+	-	-	+	?	+	+	?
**Subtotal (95% CI)**			**14**			**16**	**100.0%**	**-6.90 [-13.88, 0.08]**									
Heterogeneity: Not applicable																	
Test for overall effect: Z = 1.94 (P = 0.05)																	

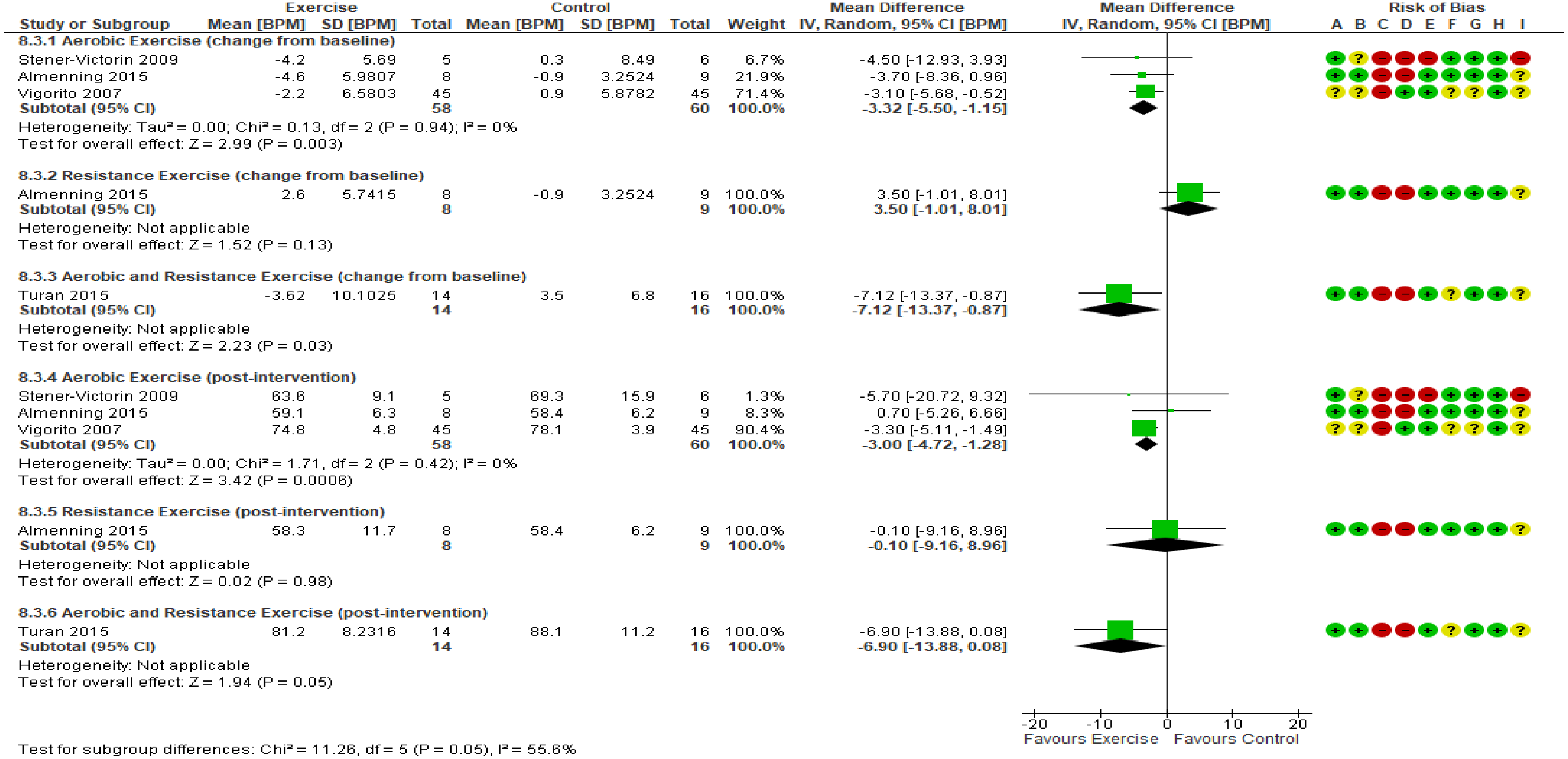

Test for subgroup differences: Chi² = 11.26, df = 5 (P = 0.05), I² = 55.6%

Risk of bias legend
(A) Random sequence generation (selection bias)
(B) Allocation concealment (selection bias)
(C) Blinding of participants and personnel (performance bias)
(D) Blinding of outcome assessment (detection bias)
(E) Incomplete outcome data (attrition bias)
(F) Selective reporting (reporting bias)
(G) Group similarity at baseline (other bias)
(H) Adherence (other bias)
(I) Contamination (other bias)

Supplementary Figure 4. A: Funnel plot of comparison: Exercise vs. Control, outcome: BMI (change from baseline). B: Funnel plot of comparison: Exercise vs. Control, outcome: BMI (post-intervention).

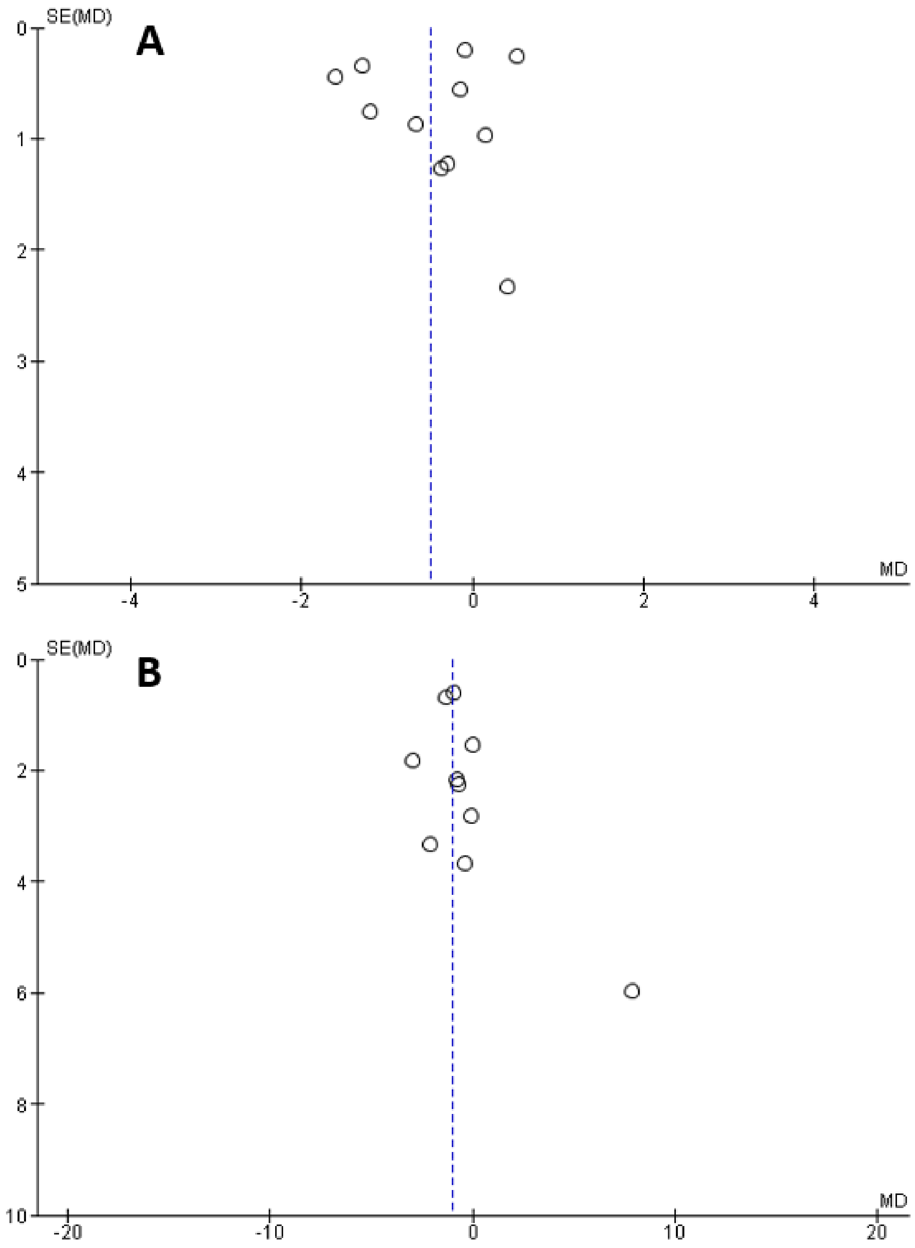

Supplementary Figure 5. Forest plot of comparison: Exercise vs. Control, change from baseline; Outcome: SF-36 Domains.

Study or Subgroup	Exercise Mean	Exercise SD	Exercise Total	Control Mean	Control SD	Control Total	Weight	Mean Difference IV, Random, 95% CI
**1.52.1 Physical Functioning**								
Sa 2015	21.1	18.7051	14	-4.6	18.534	13	23.1%	25.70 [11.65, 39.75]
Stener-Victorin 2009	0.9	10.2	29	-4	8.1	15	40.6%	4.90 [-0.63, 10.43]
Vizza 2016	6.3	6.8119	7	-4.4	6.9183	6	36.4%	10.70 [3.21, 18.19]
**Subtotal (95% CI)**			**50**			**34**	**100.0%**	**11.81 [2.36, 21.25]**
Heterogeneity: Tau² = 49.25; Chi² = 7.71, df = 2 (P = 0.02); I² = 74%								
Test for overall effect: Z = 2.45 (P = 0.01)								
**1.52.2 Role Physical**								
Sa 2015	39.3	38.2762	14	1.9	37.8955	13	22.8%	37.40 [8.65, 66.15]
Stener-Victorin 2009	-16.4	36.8	29	-6.7	27.5	15	31.8%	-9.70 [-29.01, 9.61]
Vizza 2016	0.9	2.4031	7	1.1	6.6003	6	45.4%	-0.20 [-5.77, 5.37]
**Subtotal (95% CI)**			**50**			**34**	**100.0%**	**5.33 [-13.70, 24.37]**
Heterogeneity: Tau² = 199.52; Chi² = 7.46, df = 2 (P = 0.02); I² = 73%								
Test for overall effect: Z = 0.55 (P = 0.58)								
**1.52.3 Role Emotional**								
Sa 2015	40.6	36.7174	14	10.4	36.4061	13	21.6%	30.20 [2.60, 57.80]
Stener-Victorin 2009	5.7	45.5	29	15.6	35.3	15	25.0%	-9.90 [-34.26, 14.46]
Vizza 2016	5	6.8078	7	-1.4	3.4043	6	53.4%	6.40 [0.67, 12.13]
**Subtotal (95% CI)**			**50**			**34**	**100.0%**	**7.46 [-8.78, 23.69]**
Heterogeneity: Tau² = 119.91; Chi² = 4.58, df = 2 (P = 0.10); I² = 56%								
Test for overall effect: Z = 0.90 (P = 0.37)								
**1.52.4 Bodily Pain**								
Sa 2015	12	26.3257	14	10.6	26.1462	13	13.8%	1.40 [-18.40, 21.20]
Stener-Victorin 2009	1.3	30.2	29	-3	19.7	15	24.5%	4.30 [-10.54, 19.14]
Vizza 2016	-2	8.3576	7	3.6	8.7538	6	61.7%	-5.60 [-14.95, 3.75]
**Subtotal (95% CI)**			**50**			**34**	**100.0%**	**-2.21 [-9.56, 5.13]**
Heterogeneity: Tau² = 0.00; Chi² = 1.37, df = 2 (P = 0.50); I² = 0%								
Test for overall effect: Z = 0.59 (P = 0.56)								
**1.52.5 General Health**								
Sa 2015	18.1	18.0123	14	0	18.0376	13	20.5%	18.10 [4.49, 31.71]
Stener-Victorin 2009	1.2	11.6	29	-5.5	16.8	15	42.1%	6.70 [-2.79, 16.19]
Vizza 2016	5.3	11.541	7	-4.1	6.6254	6	37.5%	9.40 [-0.66, 19.46]
**Subtotal (95% CI)**			**50**			**34**	**100.0%**	**10.05 [3.89, 16.20]**
Heterogeneity: Tau² = 0.00; Chi² = 1.84, df = 2 (P = 0.40); I² = 0%								
Test for overall effect: Z = 3.20 (P = 0.001)								
**1.52.6 Energy/Vitality**								
Sa 2015	18.2	19.0515	14	12.7	19.0305	13	24.4%	5.50 [-8.87, 19.87]
Stener-Victorin 2009	0.7	16.8	29	-1.2	16.4	15	45.7%	1.90 [-8.41, 12.21]
Vizza 2016	8.9	12.9976	7	-5.2	10.7673	6	29.9%	14.10 [1.18, 27.02]
**Subtotal (95% CI)**			**50**			**34**	**100.0%**	**6.43 [-0.82, 13.67]**
Heterogeneity: Tau² = 2.24; Chi² = 2.11, df = 2 (P = 0.35); I² = 5%								
Test for overall effect: Z = 1.74 (P = 0.08)								
**1.52.7 Social Functioning**								
Sa 2015	19.6	23.5546	14	7.7	23.4985	13	25.5%	11.90 [-5.86, 29.66]
Stener-Victorin 2009	3	23.1	29	-3.3	18	15	49.8%	6.30 [-6.10, 18.70]
Vizza 2016	14.3	17.9794	7	-8.3	15.2504	6	24.7%	22.60 [4.54, 40.66]
**Subtotal (95% CI)**			**50**			**34**	**100.0%**	**11.75 [2.56, 20.95]**
Heterogeneity: Tau² = 4.22; Chi² = 2.13, df = 2 (P = 0.35); I² = 6%								
Test for overall effect: Z = 2.50 (P = 0.01)								
**1.52.8 Mental Health**								
Sa 2015	15.4	21.4762	14	-2.5	21.1817	13	26.2%	17.90 [1.80, 34.00]
Stener-Victorin 2009	-1.1	19.8	29	-4.5	13.7	15	42.6%	3.40 [-6.60, 13.40]
Vizza 2016	12.8	14.6398	7	-5	10.8447	6	31.2%	17.80 [3.91, 31.69]
**Subtotal (95% CI)**			**50**			**34**	**100.0%**	**11.70 [1.27, 22.13]**
Heterogeneity: Tau² = 40.52; Chi² = 3.81, df = 2 (P = 0.15); I² = 47%								
Test for overall effect: Z = 2.20 (P = 0.03)								

Mean Difference IV, Random, 95% CI

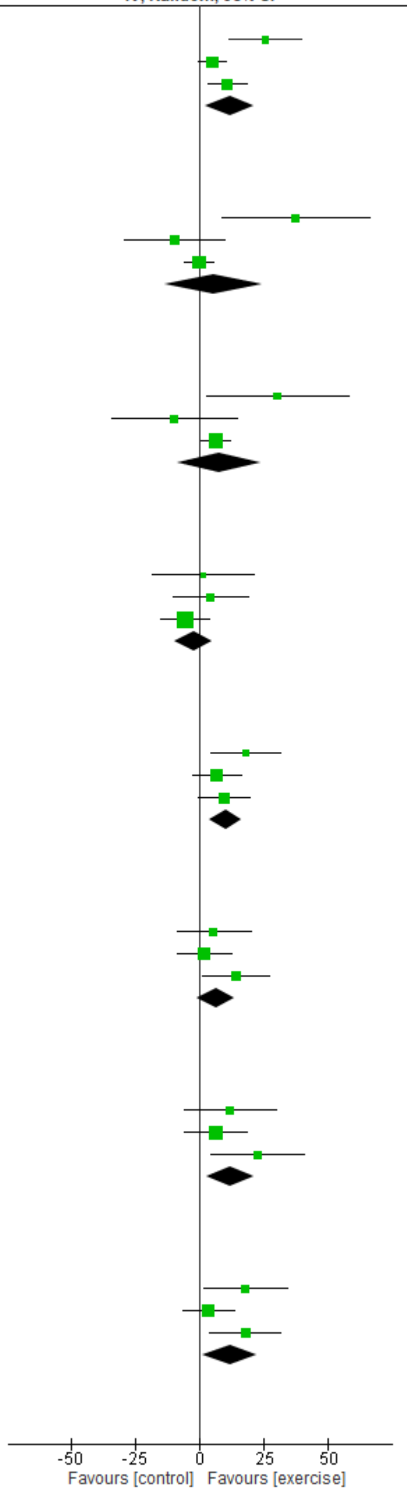

Test for subgroup differences: Chi² = 9.75, df = 7 (P = 0.20), I² = 28.2%

Supplementary Figure 6. Forest plot of comparison: Exercise and Diet vs. Control; outcome: Waist to Hip Ratio.

Study or Subgroup	Exercise and Diet Mean	Exercise and Diet SD	Exercise and Diet Total	Control Mean	Control SD	Control Total	Weight	Mean Difference IV, Random, 95% CI	Risk of Bias A B C D E F G H I
**1.20.1 Waist to hip ratio (change from baseline)**									
Guzick 1994	-0.03	0.036	6	0	0.282	6	0.2%	-0.03 [-0.26, 0.20]	? ? − − + ? + ? ?
Petranyi 2011	-0.019	0.0278	29	-0.001	0.0138	27	99.8%	-0.02 [-0.03, -0.01]	? ? − − + ? ? ? ?
**Subtotal (95% CI)**			**35**			**33**	**100.0%**	**-0.02 [-0.03, -0.01]**	
Heterogeneity: Tau² = 0.00; Chi² = 0.01, df = 1 (P = 0.92); I² = 0%									
Test for overall effect: Z = 3.11 (P = 0.002)									
**1.20.2 Waist to hip ratio (post-intervention)**									
Guzick 1994	0.92	0.0735	6	0.95	0.049	6	23.6%	-0.03 [-0.10, 0.04]	? ? − − + ? + ? ?
Petranyi 2011	0.736	0.08	29	0.745	0.07	27	76.4%	-0.01 [-0.05, 0.03]	? ? − − + ? ? ? ?
**Subtotal (95% CI)**			**35**			**33**	**100.0%**	**-0.01 [-0.05, 0.02]**	
Heterogeneity: Tau² = 0.00; Chi² = 0.26, df = 1 (P = 0.61); I² = 0%									
Test for overall effect: Z = 0.80 (P = 0.43)									

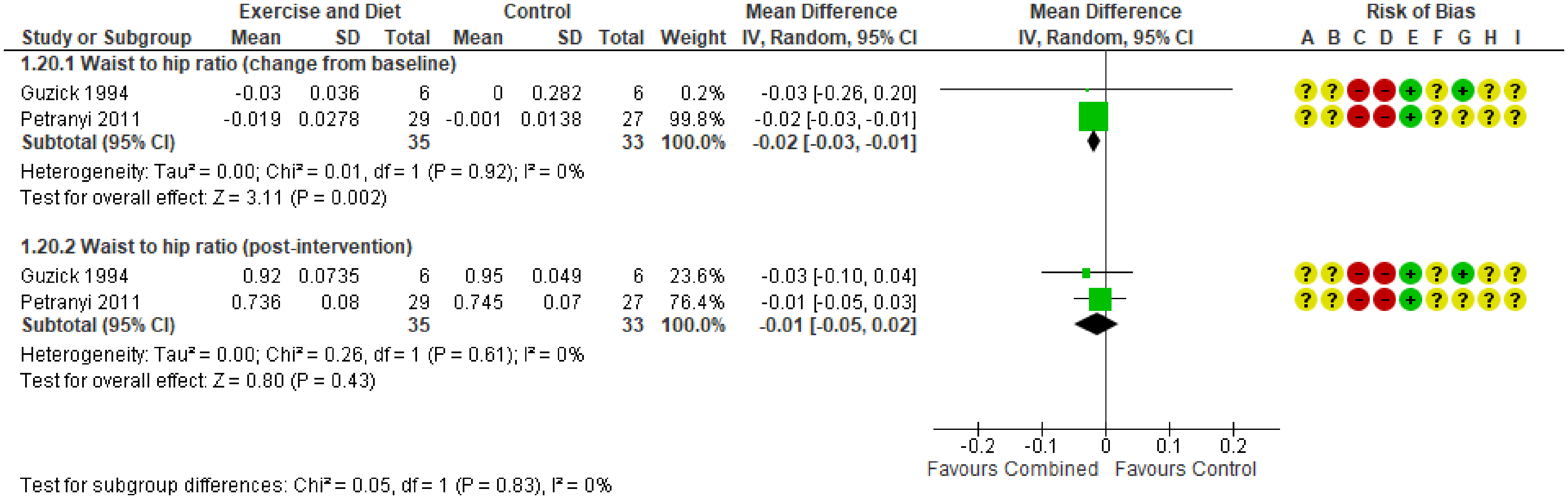

Test for subgroup differences: Chi² = 0.05, df = 1 (P = 0.83), I² = 0%

Risk of bias legend
(A) Random sequence generation (selection bias)
(B) Allocation concealment (selection bias)
(C) Blinding of participants and personnel (performance bias)
(D) Blinding of outcome assessment (detection bias)
(E) Incomplete outcome data (attrition bias)
(F) Selective reporting (reporting bias)
(G) Group similarity at baseline (other bias)
(H) Adherence (other bias)
(I) Contamination (other bias)

Supplementary Figure 7. Forest plot of comparison: Exercise and Diet vs. Control, change from baseline. Outcome: Sex-hormone binding globulin (SHBG).

Study or Subgroup	Exercise and Diet Mean	Exercise and Diet SD	Exercise and Diet Total	Control Mean	Control SD	Control Total	Weight	Std. Mean Difference IV, Random, 95% CI	Risk of Bias A	B	C	D	E	F	G	H	I
Guzick 1994	-0.13	0.13	6	0.3	0.85	6	39.7%	-0.65 [-1.83, 0.52]	?	?	-	-	+	?	+	?	?
Hoeger 2004	6.5	36.2	11	-0.1	15	9	60.3%	0.22 [-0.66, 1.10]	+	?	-	-	-	?	+	?	?
**Total (95% CI)**			**17**			**15**	**100.0%**	**-0.13 [-0.96, 0.71]**									

Heterogeneity: Tau² = 0.10; Chi² = 1.35, df = 1 (P = 0.25); I² = 26%
Test for overall effect: Z = 0.30 (P = 0.77)

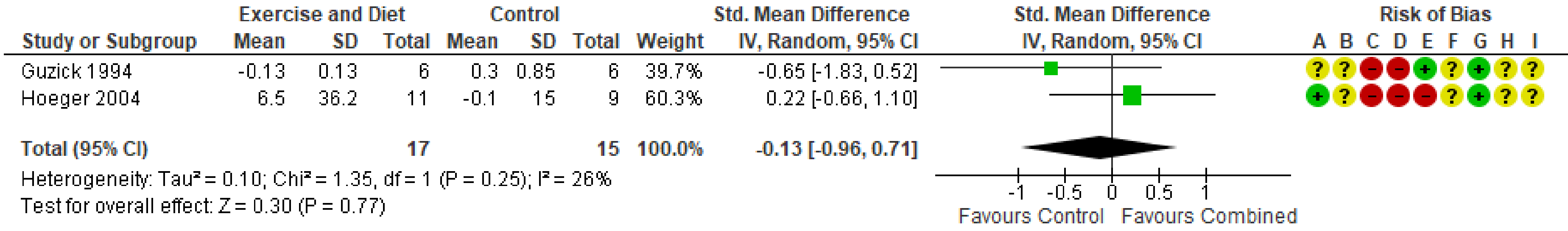

Risk of bias legend
(A) Random sequence generation (selection bias)
(B) Allocation concealment (selection bias)
(C) Blinding of participants and personnel (performance bias)
(D) Blinding of outcome assessment (detection bias)
(E) Incomplete outcome data (attrition bias)
(F) Selective reporting (reporting bias)
(G) Group similarity at baseline (other bias)
(H) Adherence (other bias)
(I) Contamination (other bias)
