# Supplementary material for: Exercise, or exercise and diet for the management of polycystic ovary syndrome: a systematic review and meta-analysis
Source: Syst Rev. 2019 Feb 12;8:51. doi: 10.1186/s13643-019-0962-3 (PMC6371542; doi:10.1186/s13643-019-0962-3)

Supplementary Meta-Analyses – Exercise versus Control

Supporting Figure: Change from baseline systolic blood pressure (mmHg); exercise versus control

	Exercise			Control				Mean Difference
Study or Subgroup	Mean [mmHg]	SD [mmHg]	Total	Mean [mmHg]	SD [mmHg]	Total	Weight	IV, Random, 95% CI [mmHg]
Stener-Victorin 2009	0.6	5.94	5	-1.4	4.62	6	22.7%	2.00 [-4.39, 8.39]
Turan 2015	-2.7	10.8508	14	-2.5	5.6	16	23.0%	-0.20 [-6.51, 6.11]
Sa 2015	-8.9	7	14	-1.2	8.8	13	24.1%	-7.70 [-13.73, -1.67]
Vigorito 2007	-3.8	10.5591	45	1.1	12.2494	45	30.1%	-4.90 [-9.63, -0.17]
**Total (95% CI)**			**78**			**80**	**100.0%**	**-2.93 [-7.06, 1.20]**

Heterogeneity: Tau² = 8.91; Chi² = 6.05, df = 3 (P = 0.11); I² = 50%
Test for overall effect: Z = 1.39 (P = 0.16)

Mean Difference
IV, Random, 95% CI [mmHg]

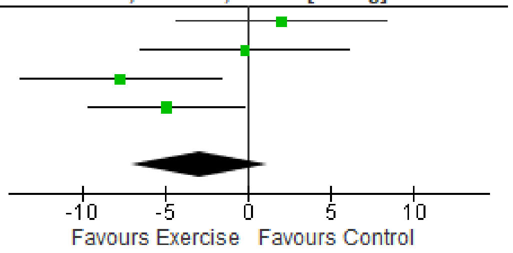

Supporting Figure: Post-intervention systolic blood pressure (mmHg); exercise versus control

	Exercise			Control				Mean Difference
Study or Subgroup	Mean [mmHg]	SD [mmHg]	Total	Mean [mmHg]	SD [mmHg]	Total	Weight	IV, Random, 95% CI [mmHg]
Stener-Victorin 2009	116.6	10.3	5	108.8	6.3	6	21.3%	7.80 [-2.54, 18.14]
Sa 2015	109.9	10.8	14	112.7	11.5	13	23.6%	-2.80 [-11.23, 5.63]
Turan 2015	117.8	9.3541	14	108.1	4.4	16	27.0%	9.70 [4.35, 15.05]
Vigorito 2007	114.1	10.6	45	119.8	9.5	45	28.1%	-5.70 [-9.86, -1.54]
**Total (95% CI)**			**78**			**80**	**100.0%**	**2.02 [-6.82, 10.86]**

Heterogeneity: Tau² = 67.80; Chi² = 22.38, df = 3 (P < 0.0001); I² = 87%
Test for overall effect: Z = 0.45 (P = 0.65)

Mean Difference
IV, Random, 95% CI [mmHg]

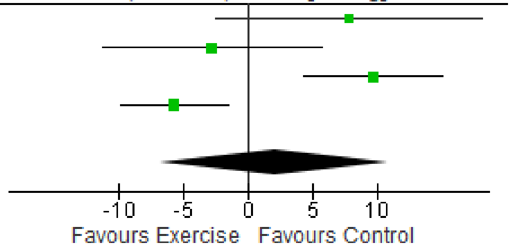

Supporting Figure: Change from baseline diastolic blood pressure (mmHg); exercise versus control

Study or Subgroup	Exercise Mean [mmHg]	Exercise SD [mmHg]	Exercise Total	Control Mean [mmHg]	Control SD [mmHg]	Control Total	Weight	Mean Difference IV, Random, 95% CI [mmHg]
Turan 2015	-3.3	11.5991	14	-3.1	7.2	16	14.0%	-0.20 [-7.23, 6.83]
Sa 2015	-5.8	7.7	14	2.3	7.4	13	18.8%	-8.10 [-13.80, -2.40]
Stener-Victorin 2009	2.8	4.73	5	2.9	3.03	6	23.3%	-0.10 [-4.90, 4.70]
Vigorito 2007	-1.7	6.1408	45	-0.3	3.7075	45	43.9%	-1.40 [-3.50, 0.70]
**Total (95% CI)**			**78**			**80**	**100.0%**	**-2.19 [-5.23, 0.85]**

Heterogeneity: Tau² = 4.34; Chi² = 5.52, df = 3 (P = 0.14); I² = 46%
Test for overall effect: Z = 1.41 (P = 0.16)

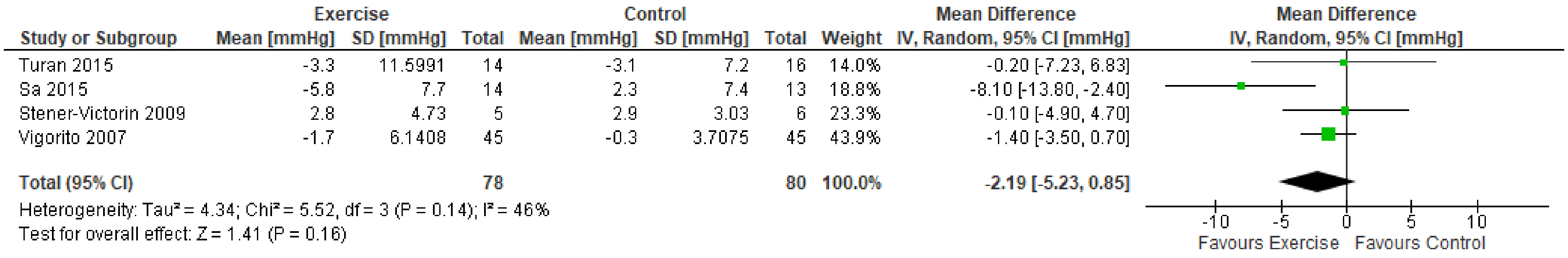

Supporting Figure: Post-intervention diastolic blood pressure (mmHg); exercise versus control

Study or Subgroup	Exercise Mean [mmHg]	Exercise SD [mmHg]	Exercise Total	Control Mean [mmHg]	Control SD [mmHg]	Control Total	Weight	Mean Difference IV, Random, 95% CI [mmHg]
Stener-Victorin 2009	67.4	6.3	5	71.2	4.1	6	14.0%	-3.80 [-10.22, 2.62]
Sa 2015	72.8	7.4	14	75.4	8.4	13	15.6%	-2.60 [-8.59, 3.39]
Turan 2015	72	9.3541	14	67.5	6.8	16	15.9%	4.50 [-1.43, 10.43]
Vigorito 2007	74.4	5	45	75.5	3.9	45	54.5%	-1.10 [-2.95, 0.75]
**Total (95% CI)**			**78**			**80**	**100.0%**	**-0.82 [-3.49, 1.84]**

Heterogeneity: Tau² = 2.50; Chi² = 4.32, df = 3 (P = 0.23); I² = 31%
Test for overall effect: Z = 0.60 (P = 0.55)

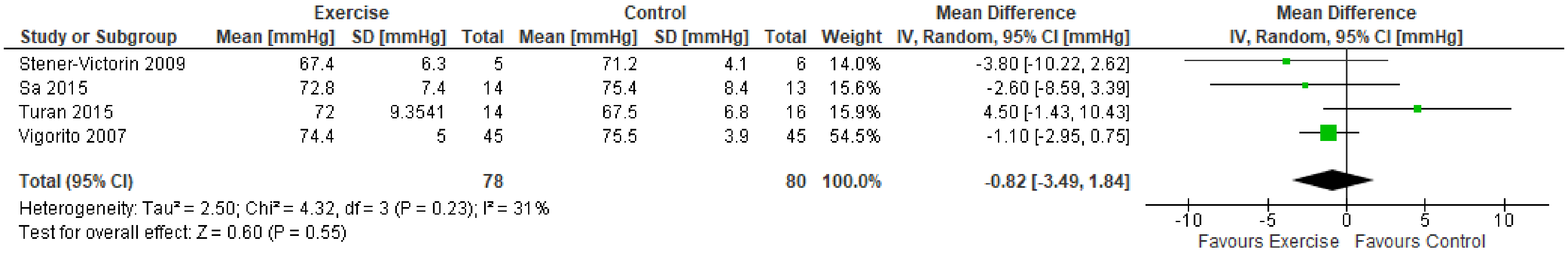

Supporting Figure: Change from baseline fasting blood glucose (mg/dL); exercise versus control

	Exercise			Control				Mean Difference
Study or Subgroup	Mean [mg/dL]	SD [mg/dL]	Total	Mean [mg/dL]	SD [mg/dL]	Total	Weight	IV, Random, 95% CI [mg/dL]
Sa 2015	3.96	10.63	14	-1.26	16.04	13	1.7%	5.22 [-5.13, 15.57]
Vizza 2016	3.6	3.3669	7	1.8	11.1978	6	2.1%	1.80 [-7.50, 11.10]
Konopka 2015	-5.3	9.0067	12	2.6	10.4561	13	3.1%	-7.90 [-15.53, -0.27]
Turan 2015	-4.4	9.3541	14	-0.4	1.2	16	7.1%	-4.00 [-8.94, 0.94]
Saremi 2016	-4.3	4.46	10	-1.1	3.94	10	11.8%	-3.20 [-6.89, 0.49]
Saremi 2013	0.9	4.28	11	0	3.39	11	14.6%	0.90 [-2.33, 4.13]
Stener-Victorin 2009	0	2.28	5	0	2.65	6	17.2%	0.00 [-2.91, 2.91]
Vigorito 2007	-1.1	5.1629	45	0.1	7.5928	45	19.4%	-1.20 [-3.88, 1.48]
Almenning 2015	-0.9	1.84	16	0	3.39	9	22.9%	-0.90 [-3.29, 1.49]
**Total (95% CI)**			**134**			**129**	**100.0%**	**-1.08 [-2.47, 0.30]**

Heterogeneity: $Tau^2 = 0.68$; $Chi^2 = 9.47$, df = 8 (P = 0.30); $I^2 = 16\%$
Test for overall effect: Z = 1.54 (P = 0.12)

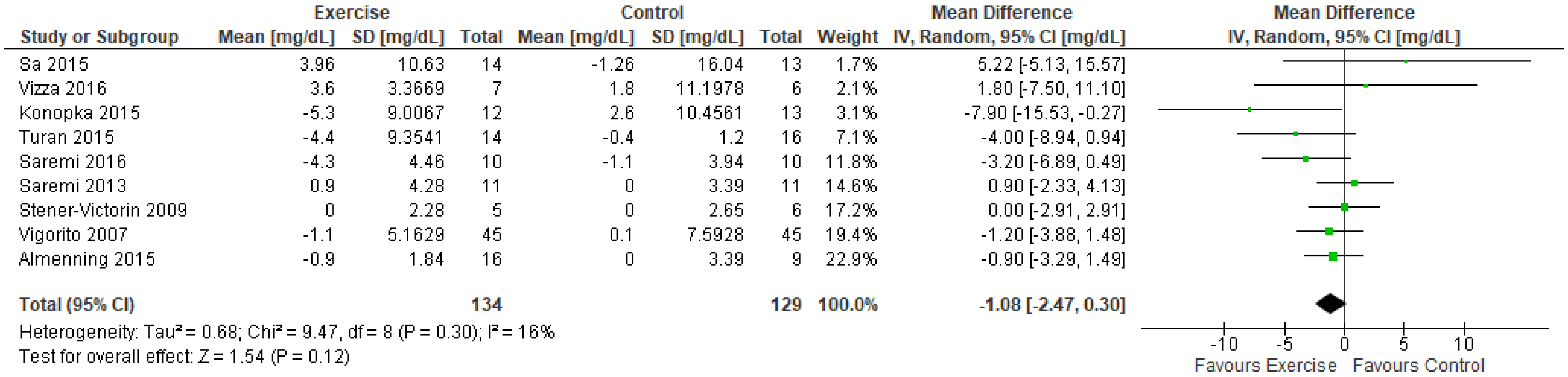

Supporting Figure: Post-intervention fasting blood glucose (mg/dL); exercise versus control

	Exercise			Control				Mean Difference
Study or Subgroup	Mean [mg/dL]	SD [mg/dL]	Total	Mean [mg/dL]	SD [mg/dL]	Total	Weight	IV, Random, 95% CI [mg/dL]
Vizza 2016	88.2	12.6	7	88.2	7.2	6	5.1%	0.00 [-10.97, 10.97]
Sa 2015	70.09	9.55	14	72.43	17.18	13	5.4%	-2.34 [-12.93, 8.25]
Saremi 2016	82.3	9.2	10	95.4	9.2	10	8.4%	-13.10 [-21.16, -5.04]
Saremi 2013	77.7	10.56	11	73.7	7.94	11	8.8%	4.00 [-3.81, 11.81]
Turan 2015	93.6	8.6058	14	97.3	9.2	16	11.8%	-3.70 [-10.08, 2.68]
Almenning 2015	90	5.8	16	90	7.2	9	14.4%	0.00 [-5.50, 5.50]
Stener-Victorin 2009	82.8	3.6	5	82.8	3.6	6	19.0%	0.00 [-4.27, 4.27]
Vigorito 2007	93.8	5.5	45	95.2	7.2	45	27.2%	-1.40 [-4.05, 1.25]
**Total (95% CI)**			**122**			**116**	**100.0%**	**-1.69 [-4.35, 0.97]**

Heterogeneity: $Tau^2 = 4.95$; $Chi^2 = 11.17$, df = 7 (P = 0.13); $I^2 = 37\%$
Test for overall effect: Z = 1.25 (P = 0.21)

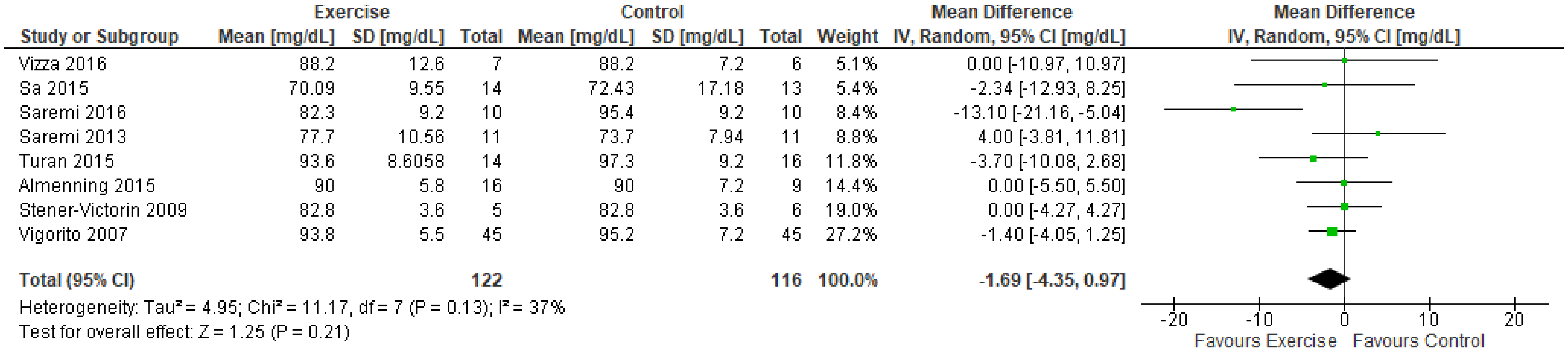

Supporting Figure: Change from baseline high-density lipoprotein cholesterol (mg/dL); exercise versus control

	Exercise			Control				Mean Difference
Study or Subgroup	Mean [mg/dL]	SD [mg/dL]	Total	Mean [mg/dL]	SD [mg/dL]	Total	Weight	IV, Random, 95% CI [mg/dL]
Stener-Victorin 2009	0	6.23	5	-7.74	7.89	6	3.9%	7.74 [-0.61, 16.09]
Vigorito 2007	1.4	13.8088	45	-1	20.0871	45	5.2%	2.40 [-4.72, 9.52]
Saremi 2013	-3.2	10.77	11	0	2.33	11	6.0%	-3.20 [-9.71, 3.31]
Almenning 2015	5.8	6.32	16	1.55	7.29	9	7.5%	4.25 [-1.43, 9.93]
Sa 2015	0.72	4.32	14	0.36	1.8	13	21.1%	0.36 [-2.11, 2.83]
Saremi 2016	-1.8	2.86	10	0.45	1.79	10	23.9%	-2.25 [-4.34, -0.16]
Turan 2015	0.6	0.3742	14	0	2	16	32.4%	0.60 [-0.40, 1.60]
**Total (95% CI)**			**115**			**110**	**100.0%**	**0.29 [-1.46, 2.04]**

Heterogeneity: Tau² = 2.21; Chi² = 12.39, df = 6 (P = 0.05); I² = 52%
Test for overall effect: Z = 0.32 (P = 0.75)

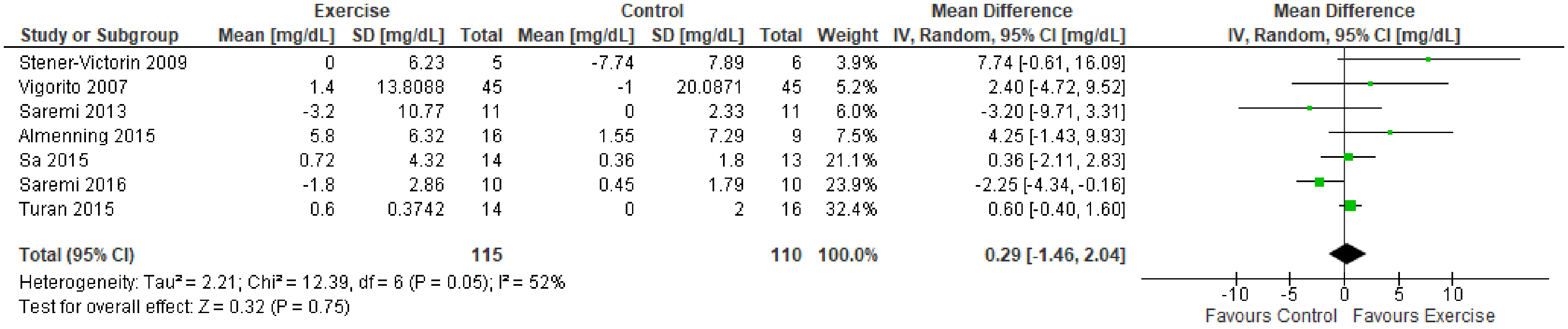

Supporting Figure: Post-intervention high-density lipoprotein cholesterol (mg/dL); exercise versus control

	Exercise			Control				Mean Difference
Study or Subgroup	Mean [mg/dL]	SD [mg/dL]	Total	Mean [mg/dL]	SD [mg/dL]	Total	Weight	IV, Random, 95% CI [mg/dL]
Almenning 2015	69.61	18.56	16	61.87	18.56	9	4.4%	7.74 [-7.42, 22.90]
Saremi 2013	50.7	24.9	11	43.2	5.47	11	4.5%	7.50 [-7.57, 22.57]
Stener-Victorin 2009	54.14	7.73	5	58	15.47	6	5.0%	-3.86 [-17.97, 10.25]
Vigorito 2007	55.2	12.9	45	58	16	45	15.6%	-2.80 [-8.80, 3.20]
Saremi 2016	52.1	6.4	10	43.45	3.4	10	19.6%	8.65 [4.16, 13.14]
Sa 2015	17.12	3.78	14	15.68	3.42	13	24.9%	1.44 [-1.28, 4.16]
Turan 2015	45.2	2.245	14	46.1	4	16	26.0%	-0.90 [-3.19, 1.39]
**Total (95% CI)**			**115**			**110**	**100.0%**	**1.87 [-1.59, 5.33]**

Heterogeneity: Tau² = 10.62; Chi² = 17.37, df = 6 (P = 0.008); I² = 65%
Test for overall effect: Z = 1.06 (P = 0.29)

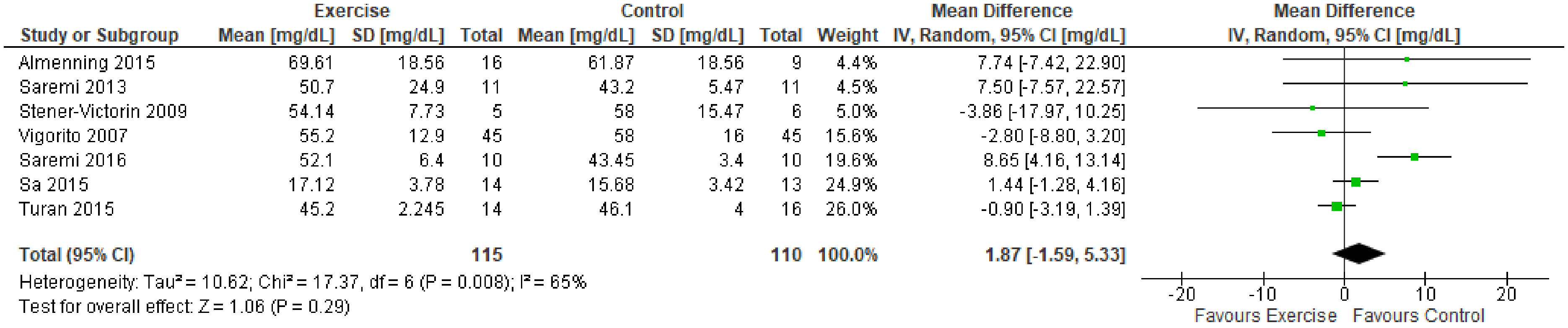

Supporting Figure: Change from baseline resting heart rate (beats/min); exercise versus control

Study or Subgroup	Exercise Mean [BPM]	Exercise SD [BPM]	Exercise Total	Control Mean [BPM]	Control SD [BPM]	Control Total	Weight	Mean Difference IV, Random, 95% CI [BPM]
Stener-Victorin 2009	-4.2	5.69	5	0.3	8.49	6	9.7%	-4.50 [-12.93, 3.93]
Turan 2015	-3.62	10.1025	14	3.5	6.8	16	15.4%	-7.12 [-13.37, -0.87]
Almenning 2015	-0.75	3.22	16	-0.9	3.2524	9	37.2%	0.15 [-2.50, 2.80]
Vigorito 2007	-2.2	6.5803	45	0.9	5.8782	45	37.8%	-3.10 [-5.68, -0.52]
**Total (95% CI)**			**80**			**76**	**100.0%**	**-2.65 [-5.55, 0.25]**

Heterogeneity: Tau² = 4.07; Chi² = 6.14, df = 3 (P = 0.10); I² = 51%
Test for overall effect: Z = 1.79 (P = 0.07)

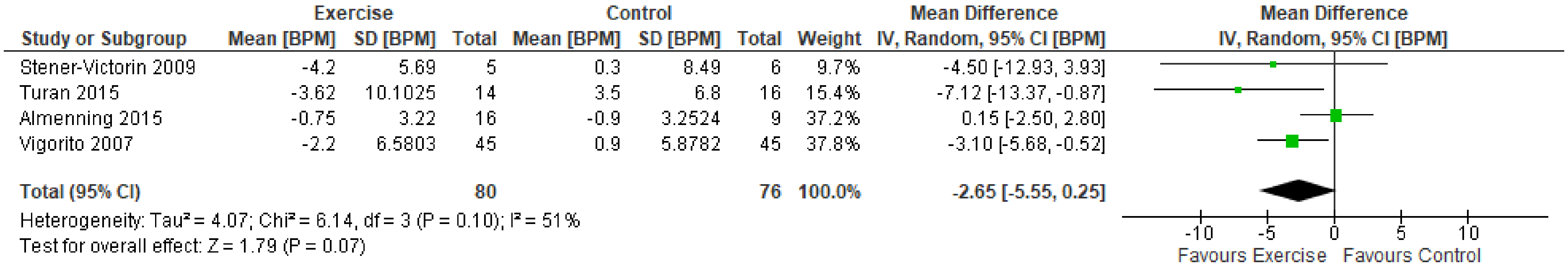

Supporting Figure: Post-intervention resting heart rate (beats/min); exercise versus control

Study or Subgroup	Exercise Mean [BPM]	Exercise SD [BPM]	Exercise Total	Control Mean [BPM]	Control SD [BPM]	Control Total	Weight	Mean Difference IV, Random, 95% CI [BPM]
Stener-Victorin 2009	63.6	9.1	5	69.3	15.9	6	1.2%	-5.70 [-20.72, 9.32]
Turan 2015	81.2	8.2316	14	88.1	11.2	16	5.7%	-6.90 [-13.88, 0.08]
Almenning 2015	58.7	9.09	16	58.4	6.2	9	7.7%	0.30 [-5.72, 6.32]
Vigorito 2007	74.8	4.8	45	78.1	3.9	45	85.4%	-3.30 [-5.11, -1.49]
**Total (95% CI)**			**80**			**76**	**100.0%**	**-3.26 [-4.93, -1.59]**

Heterogeneity: Tau² = 0.00; Chi² = 2.49, df = 3 (P = 0.48); I² = 0%
Test for overall effect: Z = 3.83 (P = 0.0001)

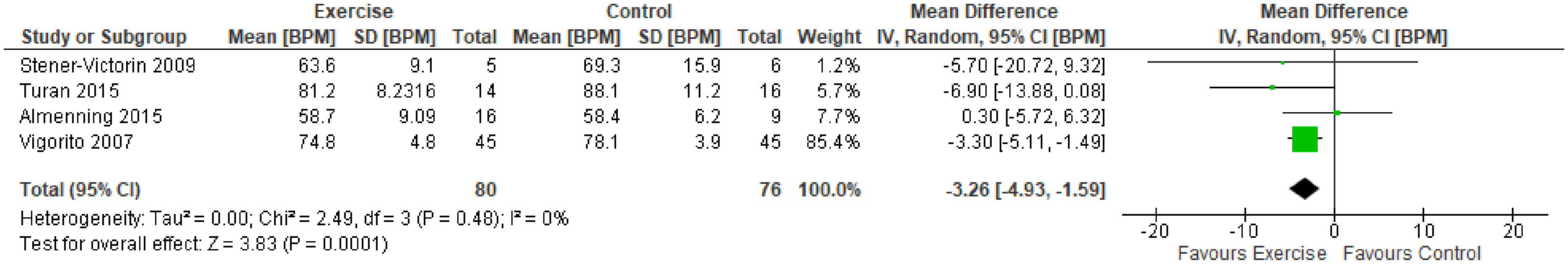

Supporting Figure: Change from baseline body mass index (kg/m^2); exercise versus control

Study or Subgroup	Experimental Mean	Experimental SD	Experimental Total	Control Mean	Control SD	Control Total	Weight	Mean Difference IV, Random, 95% CI
Vizza 2016	0.4	1.88	7	0	5.42	6	1.3%	0.40 [-4.15, 4.95]
Nasrekani 2016	-0.36	3	10	0.01	2.64	10	3.9%	-0.37 [-2.85, 2.11]
Roessler 2013	-0.4	2.24	7	-0.1	2.35	7	4.1%	-0.30 [-2.71, 2.11]
Almenning 2015	0.25	2.02	16	0.1	2.47	9	5.8%	0.15 [-1.74, 2.04]
Saremi 2013	0.19	2.57	11	0.86	1.34	11	6.6%	-0.67 [-2.38, 1.04]
Vigorito 2007	-1.3	2.4734	45	-0.1	4.4177	45	7.9%	-1.20 [-2.68, 0.28]
Turan 2015	-0.1	1.49	14	0.04	1.56	16	10.7%	-0.14 [-1.23, 0.95]
Konopka 2015	-0.5	1.3856	12	1.1	0.7211	13	12.6%	-1.60 [-2.48, -0.72]
Sa 2015	-0.6	1	14	0.7	0.8	13	14.4%	-1.30 [-1.98, -0.62]
Saremi 2016	0.49	0.581	10	-0.03	0.61	10	15.9%	0.52 [-0.00, 1.04]
Stener-Victorin 2009	0.01	0.7	30	0.11	0.63	15	16.8%	-0.10 [-0.51, 0.31]
**Total (95% CI)**			**176**			**155**	**100.0%**	**-0.49 [-1.04, 0.06]**

Heterogeneity: Tau² = 0.42; Chi² = 29.14, df = 10 (P = 0.001); I² = 66%
Test for overall effect: Z = 1.75 (P = 0.08)

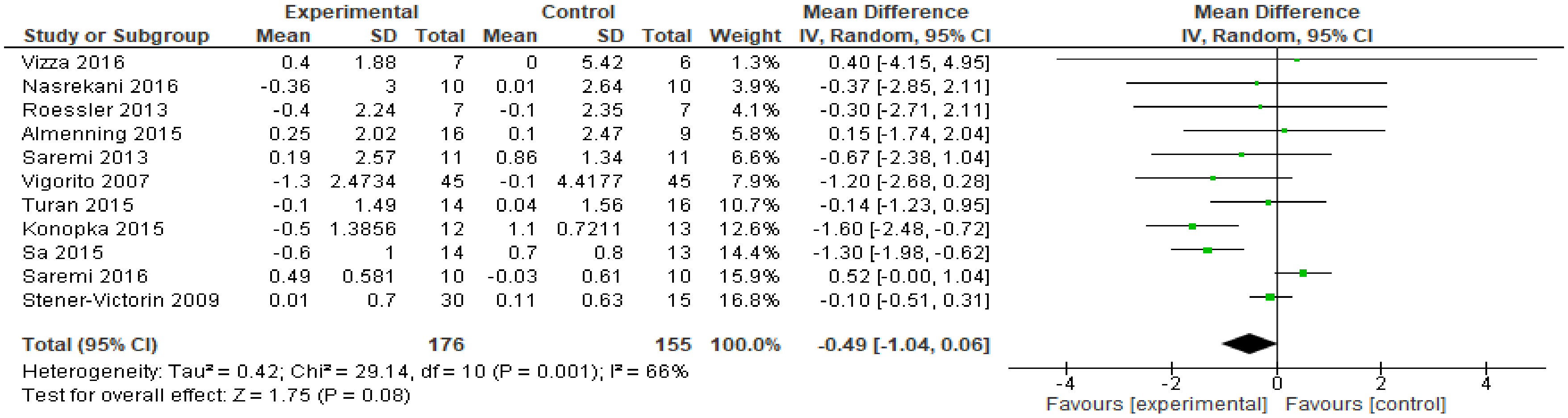

Supporting Figure: Post-intervention body mass index (kg/m^2); exercise versus control

Study or Subgroup	Exercise Mean	Exercise SD	Exercise Total	Control Mean	Control SD	Control Total	Weight	Mean Difference IV, Random, 95% CI
Vizza 2016	41.7	12.1	7	33.8	9.4	6	0.5%	7.90 [-3.80, 19.60]
Roessler 2013	34.4	6.6144	7	34.8	7.1435	7	1.2%	-0.40 [-7.61, 6.81]
Stener-Victorin 2009	26.4	4.8	5	28.5	6.2	6	1.5%	-2.10 [-8.60, 4.40]
Nasrekani 2016	28.07	6.65	10	28.11	5.89	10	2.1%	-0.04 [-5.55, 5.47]
Almenning 2015	25.7	5.62	16	26.4	5.3	9	3.2%	-0.70 [-5.12, 3.72]
Saremi 2013	28.48	6.29	11	29.26	3.55	11	3.4%	-0.78 [-5.05, 3.49]
Sa 2015	31.3	4.5	14	34.3	4.9	13	5.0%	-3.00 [-6.56, 0.56]
Turan 2015	21.7	4.1158	14	21.7	4.4	16	6.7%	0.00 [-3.05, 3.05]
Vigorito 2007	28	3.5	45	29.3	3.2	45	32.7%	-1.30 [-2.69, 0.09]
Saremi 2016	25.26	1.37	10	26.17	1.36	10	43.8%	-0.91 [-2.11, 0.29]
**Total (95% CI)**			**139**			**133**	**100.0%**	**-1.02 [-1.81, -0.23]**

Heterogeneity: Tau² = 0.00; Chi² = 4.33, df = 9 (P = 0.89); I² = 0%
Test for overall effect: Z = 2.53 (P = 0.01)

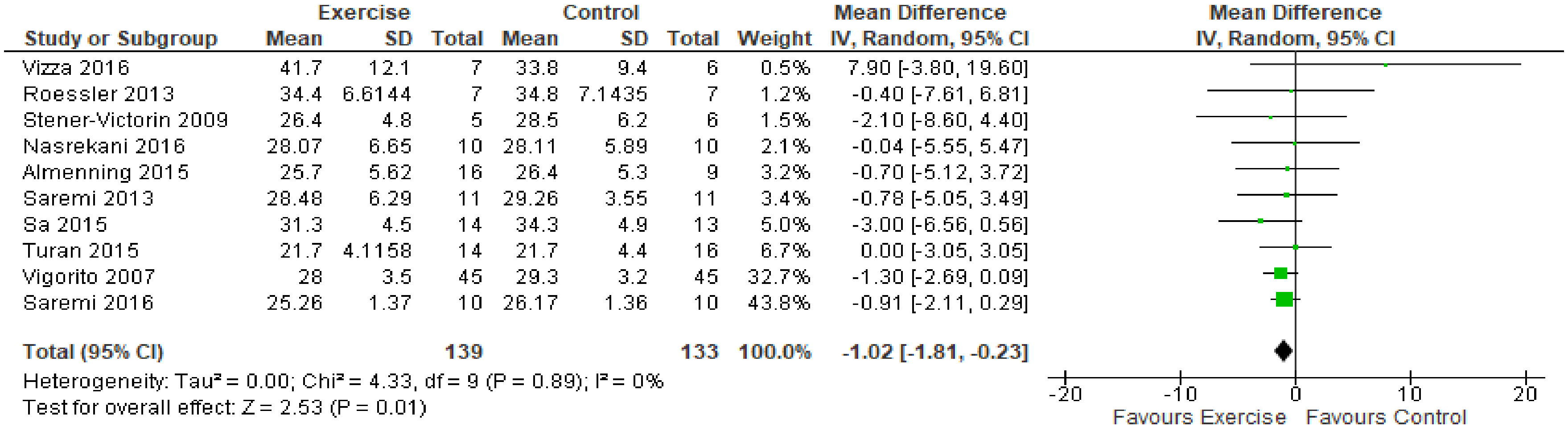

Supporting Figure: Change from baseline body mass (kg); exercise versus control

Study or Subgroup	Experimental Mean [kg]	Experimental SD [kg]	Experimental Total	Control Mean [kg]	Control SD [kg]	Control Total	Weight	Mean Difference IV, Random, 95% CI [kg]
Almenning 2015	0.65	6.23	16	0.5	2.9922	9	18.7%	0.15 [-3.47, 3.77]
Konopka 2015	-1.4	3.4641	12	3.1	2.8844	13	27.3%	-4.50 [-7.01, -1.99]
Nasrekani 2016	-0.95	6.62	10	0.05	6.18	10	10.1%	-1.00 [-6.61, 4.61]
Roessler 2013	-1.2	8.41	7	-0.5	10.75	7	3.7%	-0.70 [-10.81, 9.41]
Saremi 2013	-1.8	7.55	11	-0.8	4.12	11	11.8%	-1.00 [-6.08, 4.08]
Saremi 2016	1	2.56	10	0.4	3.46	10	25.9%	0.60 [-2.07, 3.27]
Vizza 2016	1.5	1.7156	7	0	15.44	6	2.5%	1.50 [-10.92, 13.92]
**Total (95% CI)**			**73**			**66**	**100.0%**	**-1.25 [-3.27, 0.76]**

Heterogeneity: Tau² = 2.24; Chi² = 8.98, df = 6 (P = 0.17); I² = 33%
Test for overall effect: Z = 1.22 (P = 0.22)

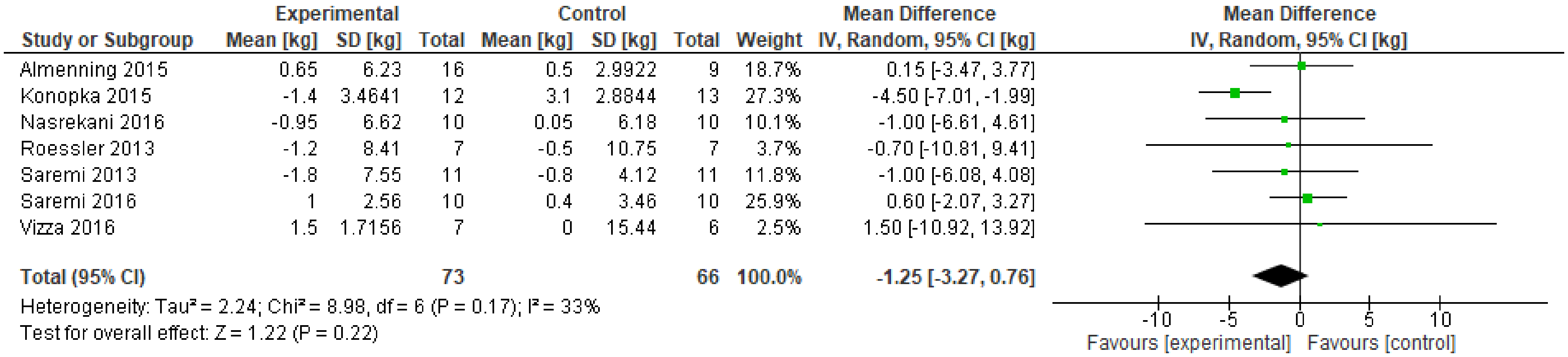

Supporting Figure: Post-intervention body mass (kg); exercise versus control

Study or Subgroup	Exercise Mean [kg]	Exercise SD [kg]	Exercise Total	Control Mean [kg]	Control SD [kg]	Control Total	Weight	Mean Difference IV, Random, 95% CI [kg]
Vizza 2016	118.9	35.4	7	86	26.8	6	1.7%	32.90 [-0.98, 66.78]
Roessler 2013	97.19	22.4499	14	95.7	27.6883	14	5.5%	1.49 [-17.18, 20.16]
Almenning 2015	73.3	17.47	16	75.5	17.5	9	9.4%	-2.20 [-16.48, 12.08]
Nasrekani 2016	70.16	14.56	10	70.18	13.78	10	12.4%	-0.02 [-12.45, 12.41]
Saremi 2013	67.8	17.23	11	67.7	9.94	11	13.9%	0.10 [-11.65, 11.85]
Saremi 2016	66.2	5.06	10	67.8	7.88	10	57.0%	-1.60 [-7.40, 4.20]
**Total (95% CI)**			**68**			**60**	**100.0%**	**-0.48 [-4.86, 3.91]**

Heterogeneity: Tau² = 0.00; Chi² = 3.99, df = 5 (P = 0.55); I² = 0%
Test for overall effect: Z = 0.21 (P = 0.83)

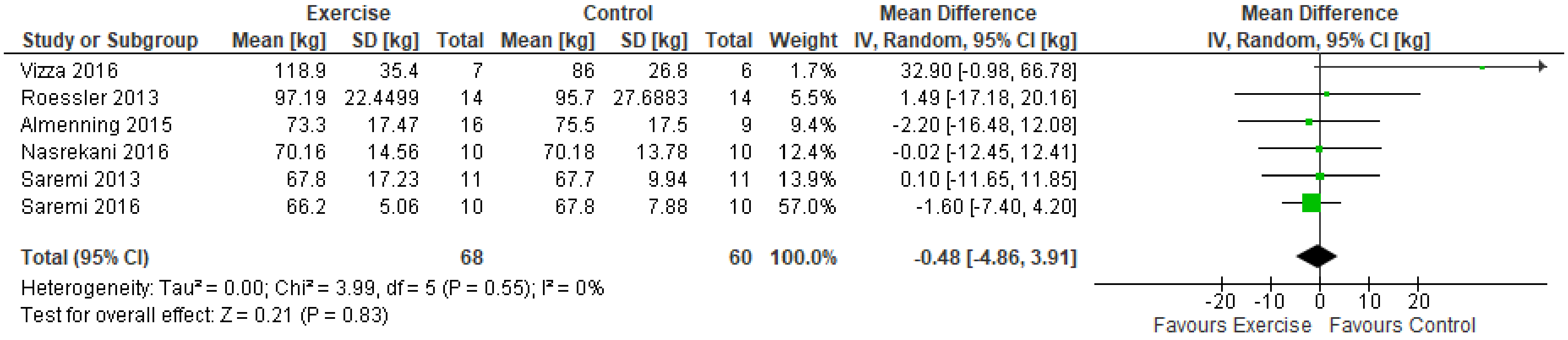

Supporting Figure: Change from baseline waist circumference (cm); exercise versus control

	Experimental			Control				Mean Difference
Study or Subgroup	Mean [cm]	SD [cm]	Total	Mean [cm]	SD [cm]	Total	Weight	IV, Random, 95% CI [cm]
Roessler 2013	-6	6.16	7	-5.2	9.25	7	3.0%	-0.80 [-9.03, 7.43]
Saremi 2013	-3.8	6.39	11	0.4	4.4	11	8.2%	-4.20 [-8.78, 0.38]
Almenning 2015	-0.5	4.96	16	-0.3	4.6834	9	10.3%	-0.20 [-4.11, 3.71]
Sa 2015	-3.7	2.5	14	3.8	6.5	13	10.8%	-7.50 [-11.27, -3.73]
Vigorito 2007	-2.7	5.137	45	-0.2	8.1501	45	15.4%	-2.50 [-5.31, 0.31]
Vizza 2016	-2.1	2.1269	7	0.7	1.2863	6	21.9%	-2.80 [-4.68, -0.92]
Turan 2015	-1.2	1.4967	14	0.2	0.4	16	30.4%	-1.40 [-2.21, -0.59]
**Total (95% CI)**			**114**			**107**	**100.0%**	**-2.62 [-4.13, -1.11]**

Heterogeneity: Tau² = 1.78; Chi² = 12.76, df = 6 (P = 0.05); I² = 53%
Test for overall effect: Z = 3.41 (P = 0.0006)

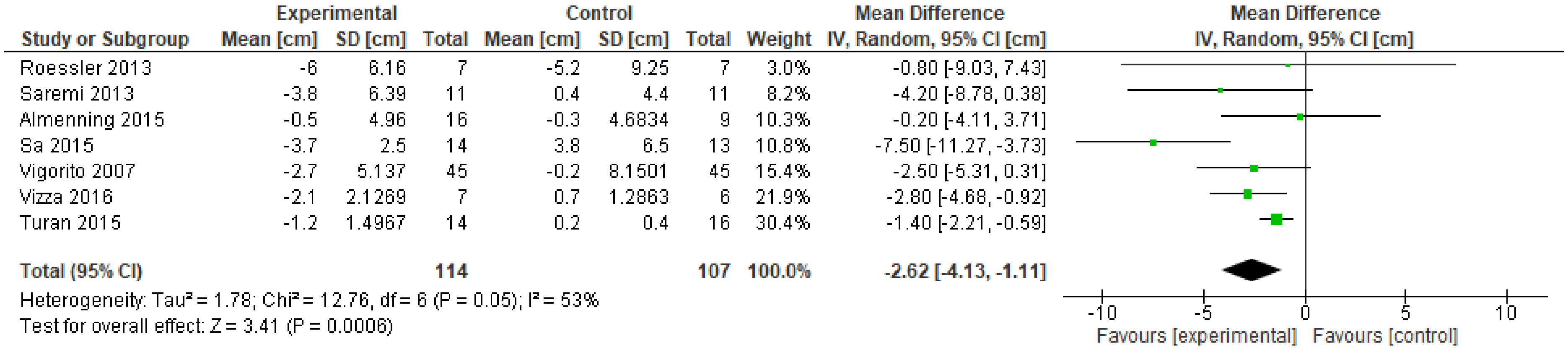

Supporting Figure: Post-intervention waist circumference (cm); exercise versus control

	Exercise			Control				Mean Difference
Study or Subgroup	Mean [cm]	SD [cm]	Total	Mean [cm]	SD [cm]	Total	Weight	IV, Random, 95% CI [cm]
Vizza 2016	121.5	29.1	7	96.6	17.2	6	1.3%	24.90 [-0.68, 50.48]
Roessler 2013	106.2	9.5247	7	106.4	17.9911	7	3.5%	-0.20 [-15.28, 14.88]
Almenning 2015	89.75	12.6	16	92.3	16.4	9	5.1%	-2.55 [-14.92, 9.82]
Saremi 2013	82.6	14.26	11	85.6	12.32	11	6.2%	-3.00 [-14.14, 8.14]
Turan 2015	67.6	7.1091	14	68.6	16	16	9.7%	-1.00 [-9.68, 7.68]
Sa 2015	89.1	10.2	14	97.9	11.3	13	10.9%	-8.80 [-16.94, -0.66]
Vigorito 2007	91.8	3.6	45	93.8	3.1	45	63.3%	-2.00 [-3.39, -0.61]
**Total (95% CI)**			**114**			**107**	**100.0%**	**-2.33 [-5.23, 0.58]**

Heterogeneity: Tau² = 2.97; Chi² = 7.06, df = 6 (P = 0.32); I² = 15%
Test for overall effect: Z = 1.57 (P = 0.12)

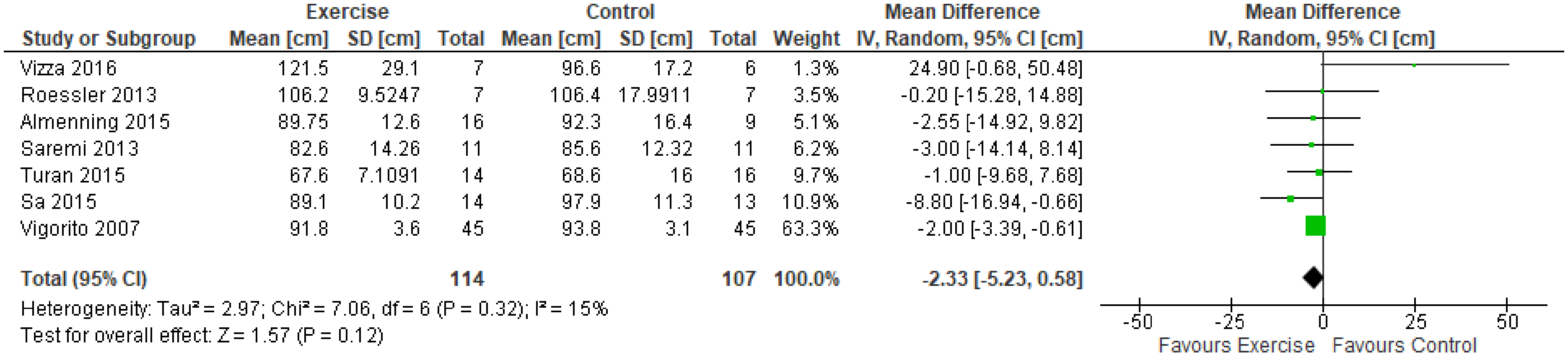

Supporting Figure: Change from baseline waist-hip-ratio; exercise versus control

	Exercise			Control				Mean Difference
Study or Subgroup	Mean	SD	Total	Mean	SD	Total	Weight	IV, Random, 95% CI
Stener-Victorin 2009	0	4.2	5	0	3.6	6	0.0%	0.00 [-4.67, 4.67]
Vigorito 2007	-0.04	0.0761	45	-0.01	0.1383	45	100.0%	-0.03 [-0.08, 0.02]
**Total (95% CI)**			**50**			**51**	**100.0%**	**-0.03 [-0.08, 0.02]**

Heterogeneity: Tau² = 0.00; Chi² = 0.00, df = 1 (P = 0.99); I² = 0%
Test for overall effect: Z = 1.27 (P = 0.20)

Mean Difference
IV, Random, 95% CI

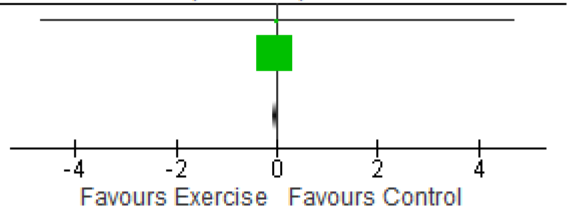

Supporting Figure: Post-intervention waist-hip-ratio; exercise versus control

	Exercise			Control				Mean Difference
Study or Subgroup	Mean	SD	Total	Mean	SD	Total	Weight	IV, Random, 95% CI
Stener-Victorin 2009	0.8	0.07	5	0.8	0.06	6	27.3%	0.00 [-0.08, 0.08]
Vigorito 2007	0.8	0.1	45	0.85	0.1	45	72.7%	-0.05 [-0.09, -0.01]
**Total (95% CI)**			**50**			**51**	**100.0%**	**-0.04 [-0.08, 0.01]**

Heterogeneity: Tau² = 0.00; Chi² = 1.23, df = 1 (P = 0.27); I² = 19%
Test for overall effect: Z = 1.63 (P = 0.10)

Mean Difference
IV, Random, 95% CI

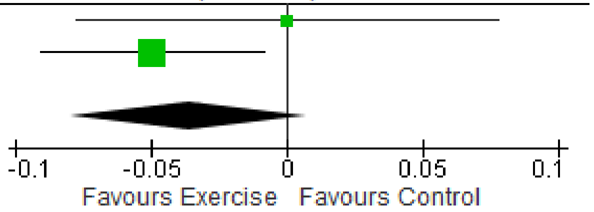

Supporting Figure: Change from baseline body fat percentage (%); exercise versus control

	Experimental			Control				Mean Difference
Study or Subgroup	Mean [%]	SD [%]	Total	Mean [%]	SD [%]	Total	Weight	IV, Random, 95% CI [%]
Almenning 2015	-1.2	3.06	16	-0.7	1.9514	9	28.3%	-0.50 [-2.47, 1.47]
Saremi 2013	-2.23	2.74	11	0.39	1.22	11	32.9%	-2.62 [-4.39, -0.85]
Vizza 2016	-0.1	1.2549	7	0.9	1.5731	6	38.9%	-1.00 [-2.56, 0.56]
**Total (95% CI)**			**34**			**26**	**100.0%**	**-1.39 [-2.61, -0.18]**

Heterogeneity: Tau² = 0.35; Chi² = 2.87, df = 2 (P = 0.24); I² = 30%
Test for overall effect: Z = 2.24 (P = 0.02)

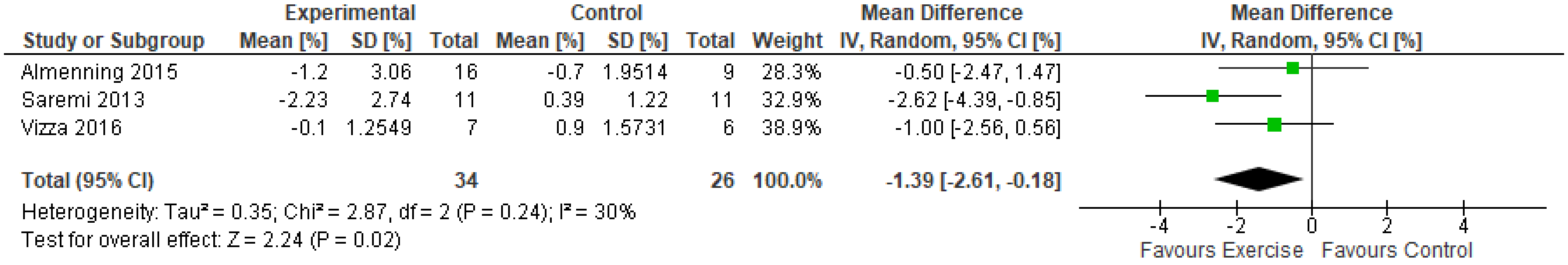

Supporting Figure: Post-intervention body fat percentage (%); exercise versus control

	Exercise			Control				Mean Difference
Study or Subgroup	Mean [%]	SD [%]	Total	Mean [%]	SD [%]	Total	Weight	IV, Random, 95% CI [%]
Vizza 2016	49.6	7.6	7	46.1	11.2	6	13.6%	3.50 [-7.08, 14.08]
Almenning 2015	30.45	8.52	16	32.9	7.3	9	32.2%	-2.45 [-8.79, 3.89]
Saremi 2013	31.03	6.78	11	36.51	2.86	11	54.2%	-5.48 [-9.83, -1.13]
**Total (95% CI)**			**34**			**26**	**100.0%**	**-3.28 [-7.39, 0.83]**

Heterogeneity: Tau² = 3.20; Chi² = 2.57, df = 2 (P = 0.28); I² = 22%
Test for overall effect: Z = 1.56 (P = 0.12)

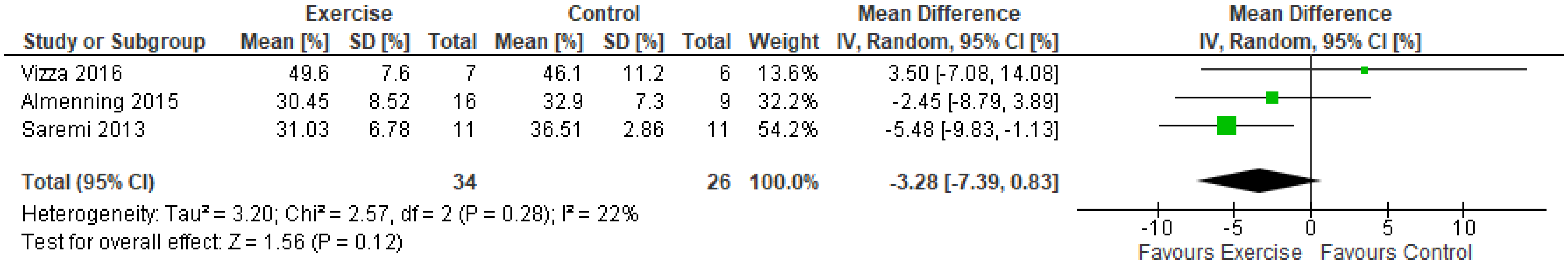

Supporting Figure: Change from baseline fat mass (kg); exercise versus control

	Experimental			Control				Mean Difference
Study or Subgroup	Mean [kg]	SD [kg]	Total	Mean [kg]	SD [kg]	Total	Weight	IV, Random, 95% CI [kg]
Almenning 2015	-0.8	4.4	16	-0.3	2.4718	9	28.3%	-0.50 [-3.19, 2.19]
Konopka 2015	-1.4	2.4249	12	2.4	2.5239	13	35.3%	-3.80 [-5.74, -1.86]
Vizza 2016	0.4	1.4749	7	1	1.8374	6	36.4%	-0.60 [-2.43, 1.23]
**Total (95% CI)**			**35**			**28**	**100.0%**	**-1.70 [-3.93, 0.53]**

Heterogeneity: Tau² = 2.69; Chi² = 6.62, df = 2 (P = 0.04); I² = 70%
Test for overall effect: Z = 1.49 (P = 0.14)

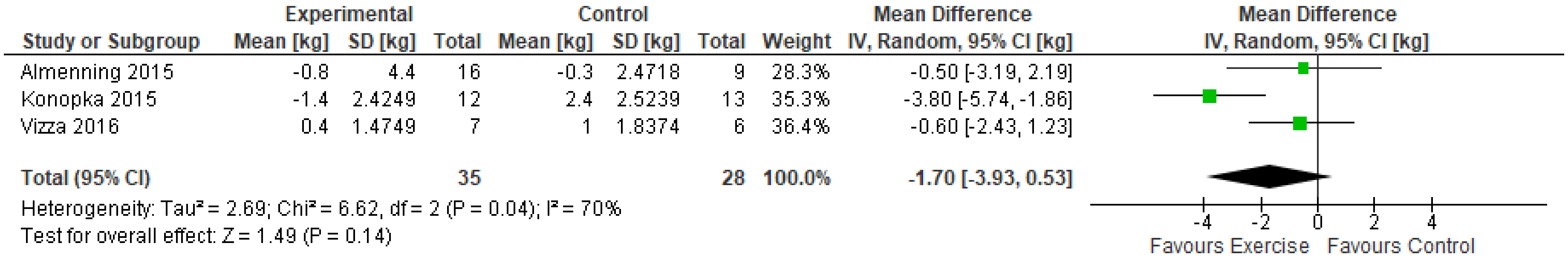

Supporting Figure: Post-intervention fat mass (kg); exercise versus control

	Exercise			Control				Mean Difference
Study or Subgroup	Mean [kg]	SD [kg]	Total	Mean [kg]	SD [kg]	Total	Weight	IV, Random, 95% CI [kg]
Vizza 2016	59	22.7	7	40.6	19.2	6	36.1%	18.40 [-4.38, 41.18]
Almenning 2015	23.55	3.9	16	25.9	11.4	9	63.9%	-2.35 [-10.04, 5.34]
**Total (95% CI)**			**23**			**15**	**100.0%**	**5.14 [-14.39, 24.68]**

Heterogeneity: Tau² = 140.06; Chi² = 2.86, df = 1 (P = 0.09); I² = 65%
Test for overall effect: Z = 0.52 (P = 0.61)

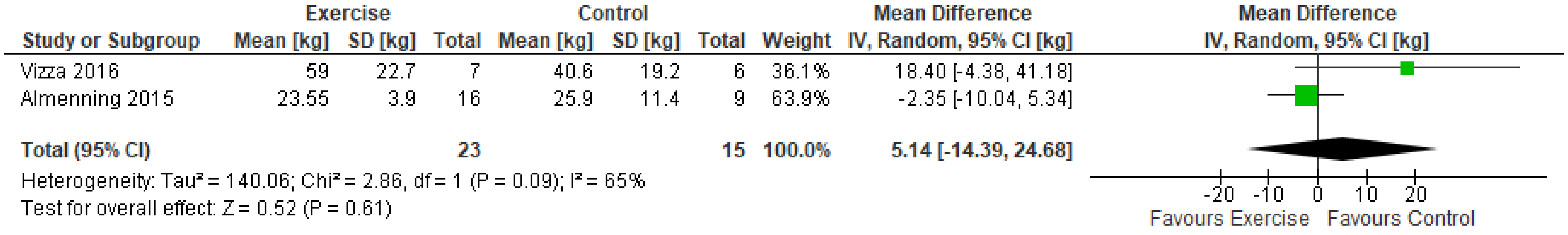

Supporting Figure: Change from baseline fat free mass (kg); exercise versus control

Study or Subgroup	Experimental Mean [kg]	Experimental SD [kg]	Experimental Total	Control Mean [kg]	Control SD [kg]	Control Total	Weight	Mean Difference IV, Random, 95% CI [kg]
Vizza 2016	1.4	2.112	7	-0.8	1.5431	6	25.5%	2.20 [0.21, 4.19]
Konopka 2015	0.4	2.0785	12	1.1	2.5239	13	28.4%	-0.70 [-2.51, 1.11]
Almenning 2015	0.6	1.35	16	0.4	0.9107	9	46.1%	0.20 [-0.69, 1.09]
**Total (95% CI)**			**35**			**28**	**100.0%**	**0.46 [-0.89, 1.81]**

Heterogeneity: $Tau^2 = 0.82$; $Chi^2 = 4.71$, df = 2 (P = 0.09); $I^2 = 58\%$
Test for overall effect: Z = 0.66 (P = 0.51)

Mean Difference IV, Random, 95% CI [kg]

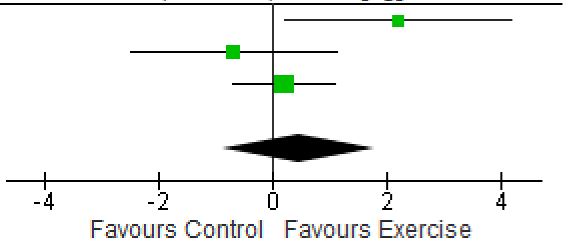

Supporting Figure: Post-intervention fat free mass (kg); exercise versus control

Study or Subgroup	Exercise Mean [kg]	Exercise SD [kg]	Exercise Total	Control Mean [kg]	Control SD [kg]	Control Total	Weight	Mean Difference IV, Random, 95% CI [kg]
Vizza 2016	58.8	11.9	7	46	10.4	6	39.2%	12.80 [0.68, 24.92]
Almenning 2015	27.35	3.9	16	27.4	3.8	9	60.8%	-0.05 [-3.18, 3.08]
**Total (95% CI)**			**23**			**15**	**100.0%**	**4.99 [-7.31, 17.28]**

Heterogeneity: $Tau^2 = 62.16$; $Chi^2 = 4.05$, df = 1 (P = 0.04); $I^2 = 75\%$
Test for overall effect: Z = 0.79 (P = 0.43)

Mean Difference IV, Random, 95% CI [kg]

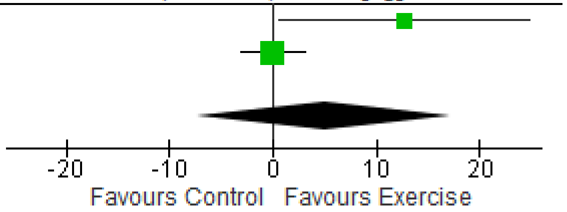

Supporting Figure: Change from baseline total testosterone (nmol/L); exercise versus control

Study or Subgroup	Exercise Mean [nmol/L]	Exercise SD [nmol/L]	Exercise Total	Control Mean [nmol/L]	Control SD [nmol/L]	Control Total	Weight	Mean Difference IV, Random, 95% CI [nmol/L]
Turan 2015	0.1	2.6192	14	0.2	2.8	16	0.6%	-0.10 [-2.04, 1.84]
Vizza 2016	0.2	0.5585	7	0.1	0.1262	6	12.4%	0.10 [-0.33, 0.53]
Almenning 2015	-0.1	0.28	16	-0.1	0.5204	9	16.7%	0.00 [-0.37, 0.37]
Vigorito 2007	-0.2	0.8927	45	-0.1	0.6263	45	22.1%	-0.10 [-0.42, 0.22]
Stener-Victorin 2009	-0.1271	0.4452	30	0.0318	0.2862	15	48.3%	-0.16 [-0.37, 0.06]
**Total (95% CI)**			**112**			**91**	**100.0%**	**-0.09 [-0.24, 0.06]**

Heterogeneity: Tau² = 0.00; Chi² = 1.39, df = 4 (P = 0.85); I² = 0%
Test for overall effect: Z = 1.14 (P = 0.25)

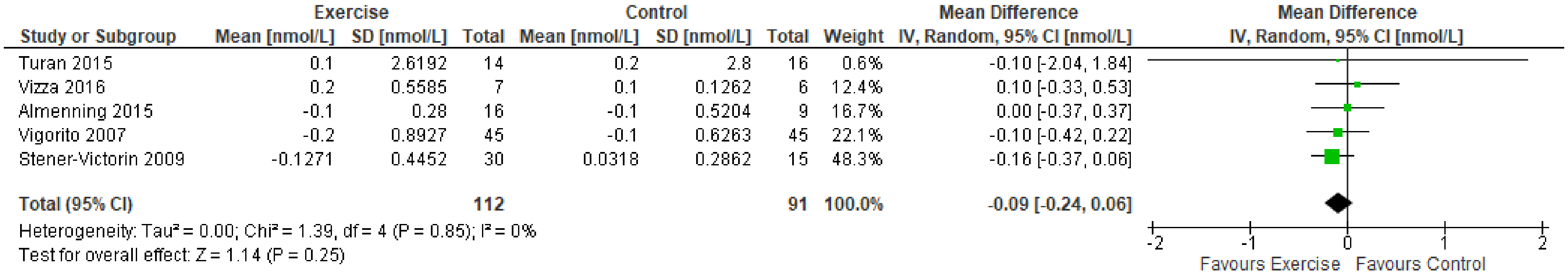

Supporting Figure: Post-intervention total testosterone (nmol/L); exercise versus control

Study or Subgroup	Exercise Mean [nmol/L]	Exercise SD [nmol/L]	Exercise Total	Control Mean [nmol/L]	Control SD [nmol/L]	Control Total	Weight	Mean Difference IV, Random, 95% CI [nmol/L]
Turan 2015	1.1	3.3675	14	1.2	0.8	16	2.1%	-0.10 [-1.91, 1.71]
Stener-Victorin 2009	2	0.3	5	2	0.7	6	13.9%	0.00 [-0.62, 0.62]
Almenning 2015	1.45	0.72	16	1.1	0.5	9	19.9%	0.35 [-0.13, 0.83]
Vizza 2016	1.7	0.5	7	1.8	0.3	6	22.2%	-0.10 [-0.54, 0.34]
Vigorito 2007	2.1	0.6	45	2.4	0.4	45	41.9%	-0.30 [-0.51, -0.09]
**Total (95% CI)**			**87**			**82**	**100.0%**	**-0.08 [-0.35, 0.19]**

Heterogeneity: Tau² = 0.03; Chi² = 6.35, df = 4 (P = 0.17); I² = 37%
Test for overall effect: Z = 0.59 (P = 0.55)

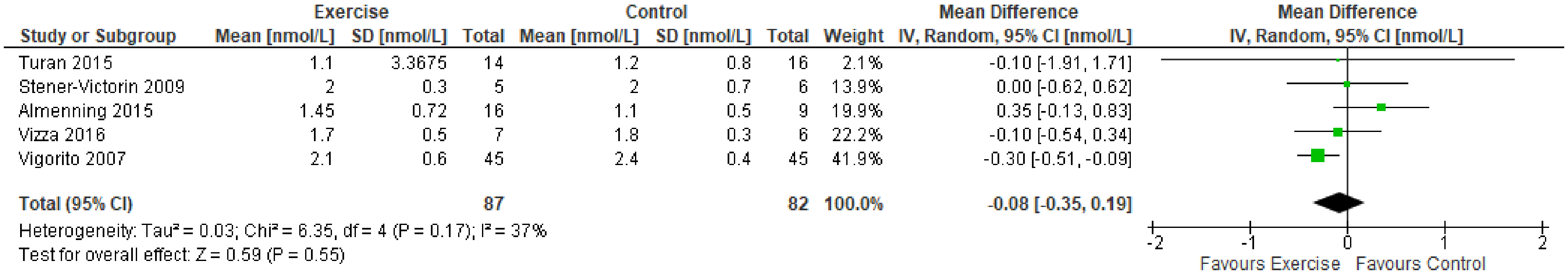

Supporting Figure: Change from baseline sex hormone binding globulin (nmol/L); exercise versus control

	Exercise			Control				Mean Difference
**Study or Subgroup**	**Mean [nmol/L]**	**SD [nmol/L]**	**Total**	**Mean [nmol/L]**	**SD [nmol/L]**	**Total**	**Weight**	**IV, Random, 95% CI [nmol/L]**
Almenning 2015	-1.6	29.75	16	-0.6	14.9609	9	21.5%	-1.00 [-18.55, 16.55]
Vizza 2016	-5	14.2804	7	0	14.43	6	22.8%	-5.00 [-20.66, 10.66]
Stener-Victorin 2009	7.3	22	30	-23.01	12.7	15	26.4%	30.31 [20.15, 40.47]
Vigorito 2007	2	8.2252	45	-1	9.4168	45	29.3%	3.00 [-0.65, 6.65]
**Total (95% CI)**			**98**			**75**	**100.0%**	**7.51 [-8.01, 23.04]**

Heterogeneity: $Tau^2 = 210.90$; $Chi^2 = 26.94$, df = 3 ($P < 0.00001$); $I^2 = 89\%$

Test for overall effect: Z = 0.95 (P = 0.34)

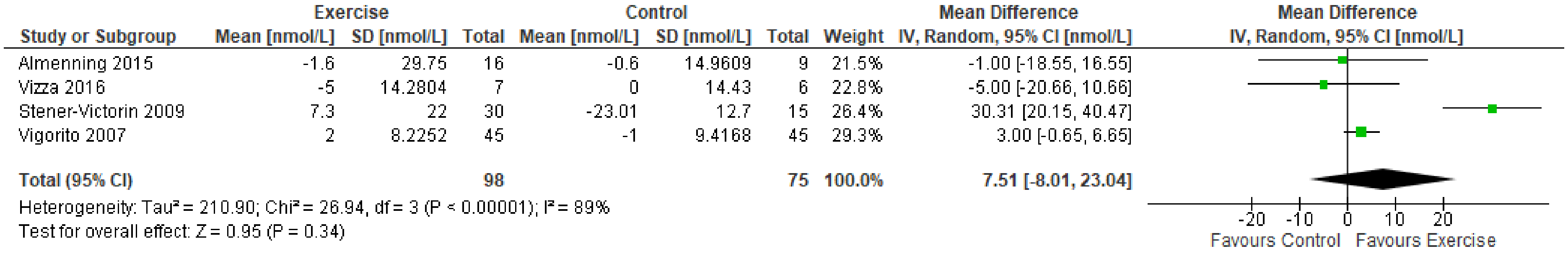

Supporting Figure: Post-intervention sex hormone binding globulin (nmol/L); exercise versus control

	Exercise			Control				Mean Difference
**Study or Subgroup**	**Mean [nmol/L]**	**SD [nmol/L]**	**Total**	**Mean [nmol/L]**	**SD [nmol/L]**	**Total**	**Weight**	**IV, Random, 95% CI [nmol/L]**
Stener-Victorin 2009	54.6	53.9	5	57.7	34.5	6	12.2%	-3.10 [-57.82, 51.62]
Almenning 2015	108.5	75.7	16	56	24.7	9	18.0%	52.50 [12.05, 92.95]
Vizza 2016	27	22	7	47	25	6	27.4%	-20.00 [-45.80, 5.80]
Vigorito 2007	29	5.8	45	28	6.9	45	42.4%	1.00 [-1.63, 3.63]
**Total (95% CI)**			**73**			**66**	**100.0%**	**4.03 [-18.57, 26.63]**

Heterogeneity: $Tau^2 = 311.90$; $Chi^2 = 8.79$, df = 3 ($P = 0.03$); $I^2 = 66\%$

Test for overall effect: Z = 0.35 (P = 0.73)

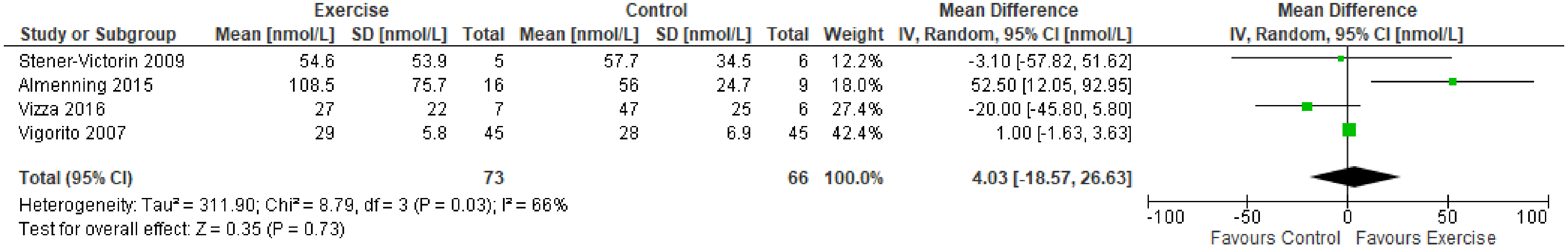

Supporting Figure: Change from baseline free testosterone (pg/mL); exercise versus control

	Exercise			Control				Mean Difference	Mean Difference
Study or Subgroup	Mean [pg/mL]	SD [pg/mL]	Total	Mean [pg/mL]	SD [pg/mL]	Total	Weight	IV, Random, 95% CI [pg/mL]	IV, Random, 95% CI [pg/mL]
Stener-Victorin 2009	-1.24	2.66	30	0.03	1.71	15	38.9%	-1.27 [-2.56, 0.02]	
Turan 2015	0	0.24	14	-0.1	0.4	16	61.1%	0.10 [-0.13, 0.33]	
**Total (95% CI)**			**44**			**31**	**100.0%**	**-0.43 [-1.74, 0.88]**	

Heterogeneity: Tau² = 0.72; Chi² = 4.22, df = 1 (P = 0.04); I² = 76%
Test for overall effect: Z = 0.65 (P = 0.52)

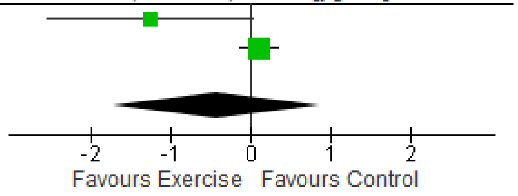

Supporting Figure: Post-intervention free testosterone (pg/mL); exercise versus control

	Exercise			Control				Mean Difference	Mean Difference
Study or Subgroup	Mean [pg/mL]	SD [pg/mL]	Total	Mean [pg/mL]	SD [pg/mL]	Total	Weight	IV, Random, 95% CI [pg/mL]	IV, Random, 95% CI [pg/mL]
Stener-Victorin 2009	5.4	2.8	5	4.4	2.8	6	1.7%	1.00 [-2.32, 4.32]	
Turan 2015	3.15	0.3742	14	2.83	0.8	16	98.3%	0.32 [-0.12, 0.76]	
**Total (95% CI)**			**19**			**22**	**100.0%**	**0.33 [-0.10, 0.77]**	

Heterogeneity: Tau² = 0.00; Chi² = 0.16, df = 1 (P = 0.69); I² = 0%
Test for overall effect: Z = 1.50 (P = 0.13)

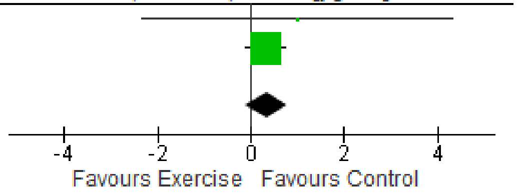

Supporting Figure: Change from baseline free androgen index; exercise versus control

Study or Subgroup	Exercise Mean	Exercise SD	Exercise Total	Control Mean	Control SD	Control Total	Weight	Mean Difference IV, Random, 95% CI
Stener-Victorin 2009	1.7	2.42	5	-0.4	2.68	6	6.9%	2.10 [-0.92, 5.12]
Vigorito 2007	0.2	1.6844	45	-0.1	8.8614	45	9.1%	0.30 [-2.34, 2.94]
Vizza 2016	1.2	2.5528	7	0.2	0.3145	6	17.3%	1.00 [-0.91, 2.91]
Almenning 2015	-0.15	0.53	16	0	1.431	9	66.8%	-0.15 [-1.12, 0.82]
**Total (95% CI)**			**73**			**66**	**100.0%**	**0.24 [-0.55, 1.04]**

Heterogeneity: Tau² = 0.00; Chi² = 2.69, df = 3 (P = 0.44); I² = 0%

Test for overall effect: Z = 0.61 (P = 0.55)

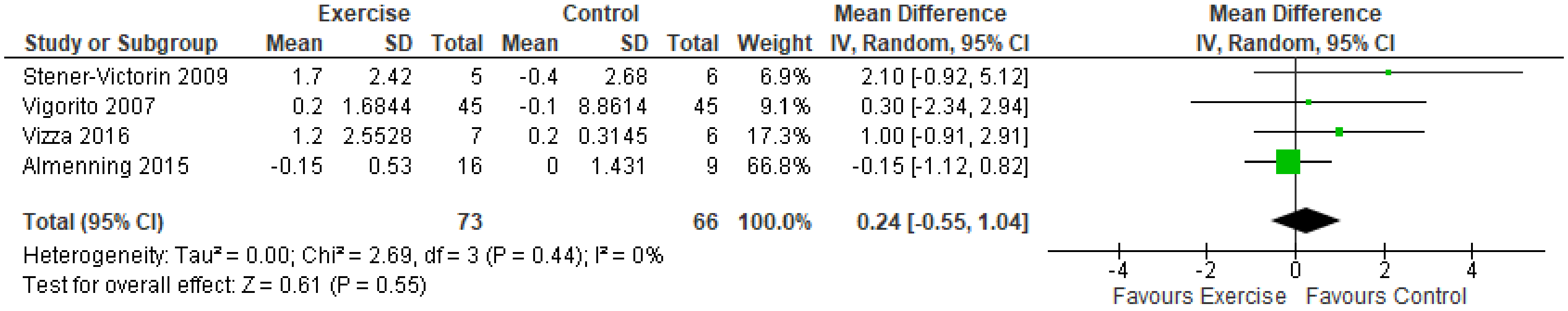

Supporting Figure: Post-intervention free androgen index; exercise versus control

Study or Subgroup	Exercise Mean	Exercise SD	Exercise Total	Control Mean	Control SD	Control Total	Weight	Mean Difference IV, Random, 95% CI
Vizza 2016	9.8	6.6	7	4.7	2.7	6	9.2%	5.10 [-0.25, 10.45]
Stener-Victorin 2009	8.8	3.3	5	5.4	5.2	6	10.1%	3.40 [-1.67, 8.47]
Almenning 2015	2	1.44	16	2.6	2.5	9	37.4%	-0.60 [-2.38, 1.18]
Vigorito 2007	8.7	3.4	45	8.5	3.5	45	43.3%	0.20 [-1.23, 1.63]
**Total (95% CI)**			**73**			**66**	**100.0%**	**0.68 [-1.09, 2.44]**

Heterogeneity: Tau² = 1.34; Chi² = 5.53, df = 3 (P = 0.14); I² = 46%

Test for overall effect: Z = 0.75 (P = 0.45)

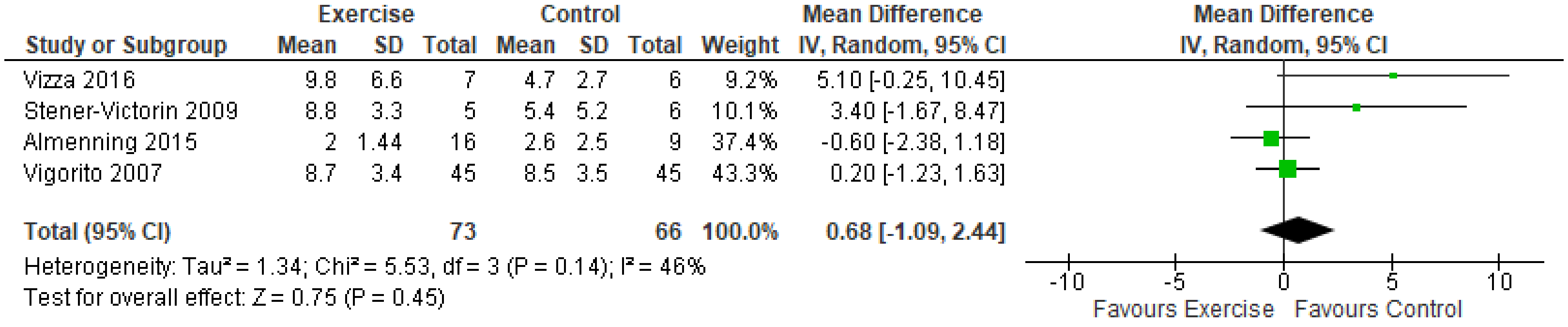

Supporting Figure: Change from baseline Ferriman-Gallwey score; exercise versus control

Study or Subgroup	Exercise Mean [mL]	Exercise SD [mL]	Exercise Total	Control Mean [mL]	Control SD [mL]	Control Total	Weight	Mean Difference IV, Random, 95% CI [mL]
Stener-Victorin 2009	0.72	3.54	30	1.4	3.66	15	41.4%	-0.68 [-2.92, 1.56]
Vigorito 2007	-0.4	4.5688	45	0.2	4.5605	45	58.6%	-0.60 [-2.49, 1.29]
**Total (95% CI)**			**75**			**60**	**100.0%**	**-0.63 [-2.08, 0.81]**

Heterogeneity: Tau² = 0.00; Chi² = 0.00, df = 1 (P = 0.96); I² = 0%
Test for overall effect: Z = 0.86 (P = 0.39)

Mean Difference
IV, Random, 95% CI [mL]

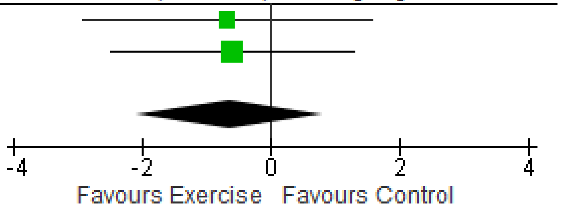

Supporting Figure: Post-intervention Ferriman-Gallwey score; exercise versus control

Study or Subgroup	Exercise Mean [mL]	Exercise SD [mL]	Exercise Total	Control Mean [mL]	Control SD [mL]	Control Total	Weight	Mean Difference IV, Random, 95% CI [mL]
Stener-Victorin 2009	12.6	9.1	5	10.8	5	6	2.1%	1.80 [-7.12, 10.72]
Vigorito 2007	11.5	3.1	45	12.3	3.2	45	97.9%	-0.80 [-2.10, 0.50]
**Total (95% CI)**			**50**			**51**	**100.0%**	**-0.75 [-2.03, 0.54]**

Heterogeneity: Tau² = 0.00; Chi² = 0.32, df = 1 (P = 0.57); I² = 0%
Test for overall effect: Z = 1.13 (P = 0.26)

Mean Difference
IV, Random, 95% CI [mL]

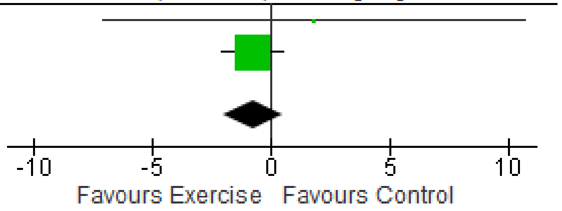

Supporting Figure: Change from baseline oestradiol (pmol/L); exercise versus control

Study or Subgroup	Exercise Mean [pmol/L]	Exercise SD [pmol/L]	Exercise Total	Control Mean [pmol/L]	Control SD [pmol/L]	Control Total	Weight	Mean Difference IV, Random, 95% CI [pmol/L]
Stener-Victorin 2009	-69.0148	175.47	30	41.12	187.96	15	9.9%	-110.13 [-224.11, 3.84]
Konopka 2015	-31.57	91.5562	12	45.52	134.9918	13	14.1%	-77.09 [-166.91, 12.73]
Turan 2015	27.4	69.2207	14	0.12	0.4	16	33.6%	27.28 [-8.98, 63.54]
Vigorito 2007	-5	44.0048	45	-2	38.6274	45	42.4%	-3.00 [-20.11, 14.11]
**Total (95% CI)**			**101**			**89**	**100.0%**	**-13.94 [-54.53, 26.64]**

Heterogeneity: Tau² = 935.44; Chi² = 8.66, df = 3 (P = 0.03); I² = 65%
Test for overall effect: Z = 0.67 (P = 0.50)

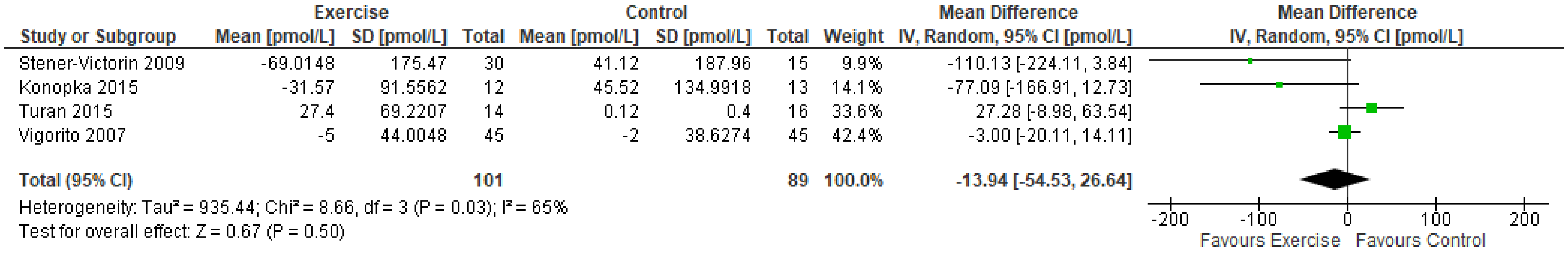

Supporting Figure: Post-intervention oestradiol (pmol/L); exercise versus control

Study or Subgroup	Exercise Mean [pmol/L]	Exercise SD [pmol/L]	Exercise Total	Control Mean [pmol/L]	Control SD [pmol/L]	Control Total	Weight	Mean Difference IV, Random, 95% CI [pmol/L]
Turan 2015	63.5	52.3832	14	56.8	15.2	16	16.5%	6.70 [-21.73, 35.13]
Vigorito 2007	115	32	45	116	29	45	83.5%	-1.00 [-13.62, 11.62]
**Total (95% CI)**			**59**			**61**	**100.0%**	**0.27 [-11.27, 11.80]**

Heterogeneity: Tau² = 0.00; Chi² = 0.24, df = 1 (P = 0.63); I² = 0%
Test for overall effect: Z = 0.05 (P = 0.96)

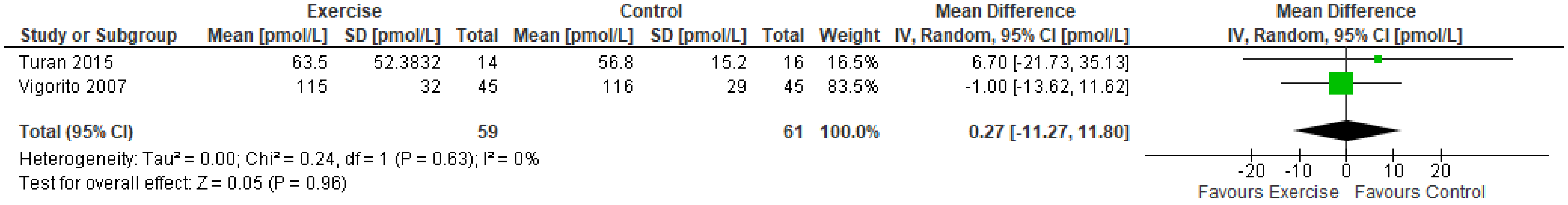

Supporting Figure: Change from baseline dehydroepiandrosterone sulfate (μmol/L); exercise versus control

	Exercise			Control				Mean Difference
Study or Subgroup	Mean [umol/L]	SD [umol/L]	Total	Mean [umol/L]	SD [umol/L]	Total	Weight	IV, Random, 95% CI [umol/L]
Almenning 2015	-1.82	1.42	16	-1	1.5611	9	64.0%	-0.82 [-2.05, 0.41]
Stener-Victorin 2009	-1.324	3.641	30	-1.125	1.986	15	36.0%	-0.20 [-1.84, 1.45]
**Total (95% CI)**			**46**			**24**	**100.0%**	**-0.60 [-1.58, 0.39]**

Heterogeneity: Tau² = 0.00; Chi² = 0.35, df = 1 (P = 0.55); I² = 0%
Test for overall effect: Z = 1.18 (P = 0.24)

Mean Difference
IV, Random, 95% CI [umol/L]

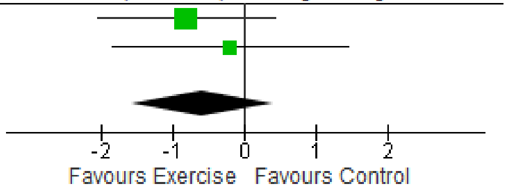

Supporting Figure: Post-intervention dehydroepiandrosterone sulfate (μmol/L); exercise versus control

	Exercise			Control				Mean Difference
Study or Subgroup	Mean [umol/L]	SD [umol/L]	Total	Mean [umol/L]	SD [umol/L]	Total	Weight	IV, Random, 95% CI [umol/L]
Almenning 2015	5.7	2.76	16	6.3	2.7	9	56.0%	-0.60 [-2.82, 1.62]
Stener-Victorin 2009	4.8	2.2	5	4.5	2	6	44.0%	0.30 [-2.21, 2.81]
**Total (95% CI)**			**21**			**15**	**100.0%**	**-0.20 [-1.87, 1.46]**

Heterogeneity: Tau² = 0.00; Chi² = 0.28, df = 1 (P = 0.60); I² = 0%
Test for overall effect: Z = 0.24 (P = 0.81)

Mean Difference
IV, Random, 95% CI [umol/L]

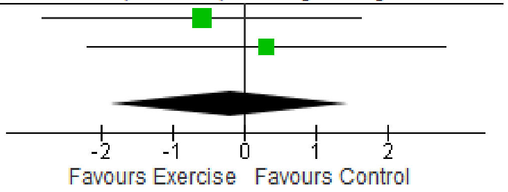

Supporting Figure: Change from baseline luteinising hormone (IU/L); exercise versus control

Study or Subgroup	Exercise Mean [IU/L]	Exercise SD [IU/L]	Exercise Total	Control Mean [IU/L]	Control SD [IU/L]	Control Total	Weight	Mean Difference IV, Random, 95% CI [IU/L]
Stener-Victorin 2009	-0.45	4.33	30	-1.63	7.98	15	15.5%	1.18 [-3.15, 5.51]
Nasrekani 2016	-3.46	2.92	10	0	2.82	10	25.1%	-3.46 [-5.98, -0.94]
Vigorito 2007	-0.7	4.572	45	-0.4	4.2389	45	29.5%	-0.30 [-2.12, 1.52]
Turan 2015	1.7	3.3675	14	0.1	0.4	16	29.8%	1.60 [-0.17, 3.37]
**Total (95% CI)**			**99**			**86**	**100.0%**	**-0.30 [-2.54, 1.95]**

Heterogeneity: Tau² = 3.57; Chi² = 10.76, df = 3 (P = 0.01); I² = 72%
Test for overall effect: Z = 0.26 (P = 0.80)

Mean Difference IV, Random, 95% CI [IU/L]

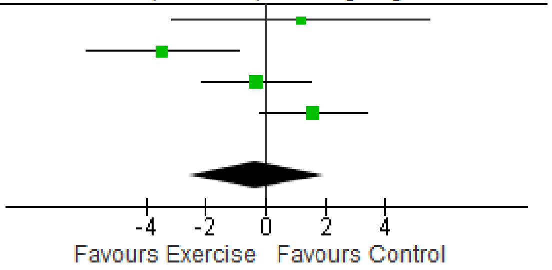

Supporting Figure: Post-intervention luteinising hormone (IU/L); exercise versus control

Study or Subgroup	Exercise Mean [IU/L]	Exercise SD [IU/L]	Exercise Total	Control Mean [IU/L]	Control SD [IU/L]	Control Total	Weight	Mean Difference IV, Random, 95% CI [IU/L]
Nasrekani 2016	9.33	6.04	10	12.86	6.32	10	8.7%	-3.53 [-8.95, 1.89]
Turan 2015	8.8	5.8744	14	12.4	4.8	16	15.0%	-3.60 [-7.47, 0.27]
Stener-Victorin 2009	5.8	1.5	5	5.8	2.2	6	30.6%	0.00 [-2.20, 2.20]
Vigorito 2007	23.5	3.2	45	23.1	2.9	45	45.7%	0.40 [-0.86, 1.66]
**Total (95% CI)**			**74**			**77**	**100.0%**	**-0.66 [-2.39, 1.06]**

Heterogeneity: Tau² = 1.29; Chi² = 5.29, df = 3 (P = 0.15); I² = 43%
Test for overall effect: Z = 0.75 (P = 0.45)

Mean Difference IV, Random, 95% CI [IU/L]

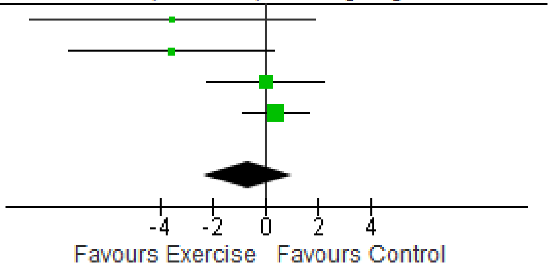

Supporting Figure: Change from baseline follicle stimulating hormone (IU/L); exercise versus control

	Exercise			Control				Mean Difference
Study or Subgroup	Mean [IU/L]	SD [IU/L]	Total	Mean [IU/L]	SD [IU/L]	Total	Weight	IV, Random, 95% CI [IU/L]
Stener-Victorin 2009	0.26	1.57	30	-0.31	1.65	15	9.1%	0.57 [-0.44, 1.58]
Vigorito 2007	-0.2	2.1194	45	0.1	1.9314	45	13.1%	-0.30 [-1.14, 0.54]
Nasrekani 2016	0.03	0.64	10	-0.08	0.71	10	26.2%	0.11 [-0.48, 0.70]
Turan 2015	0.4	0.7483	14	0.04	0.32	16	51.6%	0.36 [-0.06, 0.78]
**Total (95% CI)**			**99**			**86**	**100.0%**	**0.23 [-0.08, 0.53]**

Heterogeneity: Tau² = 0.00; Chi² = 2.50, df = 3 (P = 0.48); I² = 0%

Test for overall effect: Z = 1.47 (P = 0.14)

Mean Difference
IV, Random, 95% CI [IU/L]

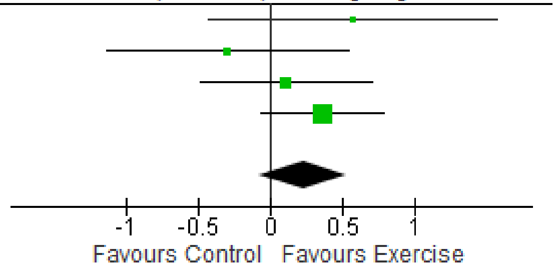

Supporting Figure: Post-intervention follicle stimulating hormone (IU/L); exercise versus control

	Exercise			Control				Mean Difference
Study or Subgroup	Mean [IU/L]	SD [IU/L]	Total	Mean [IU/L]	SD [IU/L]	Total	Weight	IV, Random, 95% CI [IU/L]
Stener-Victorin 2009	4.1	1.3	5	3.9	1.3	6	6.2%	0.20 [-1.34, 1.74]
Nasrekani 2016	6.63	1.2	10	6.55	1.58	10	9.7%	0.08 [-1.15, 1.31]
Turan 2015	5	1.1225	14	5.3	0.8	16	29.3%	-0.30 [-1.01, 0.41]
Vigorito 2007	10.3	1.3	45	10.2	1.2	45	54.8%	0.10 [-0.42, 0.62]
**Total (95% CI)**			**74**			**77**	**100.0%**	**-0.01 [-0.40, 0.37]**

Heterogeneity: Tau² = 0.00; Chi² = 0.91, df = 3 (P = 0.82); I² = 0%

Test for overall effect: Z = 0.07 (P = 0.95)

Mean Difference
IV, Random, 95% CI [IU/L]

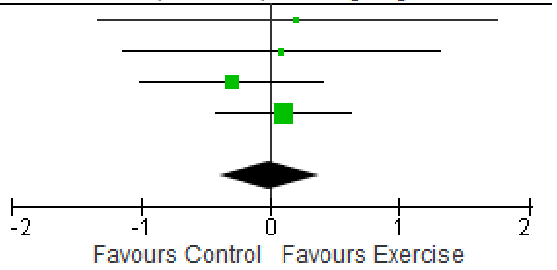

Supporting Figure: Change from baseline luteinising hormone/follicle stimulating hormone ratio; exercise versus control

Study or Subgroup	Exercise Mean	Exercise SD	Exercise Total	Control Mean	Control SD	Control Total	Weight	Mean Difference IV, Random, 95% CI
Stener-Victorin 2009	-0.6	0.58	5	-0.4	0.75	6	19.9%	-0.20 [-0.99, 0.59]
Turan 2015	0.02	0.7483	14	0	0.04	16	80.1%	0.02 [-0.37, 0.41]
**Total (95% CI)**			**19**			**22**	**100.0%**	**-0.02 [-0.38, 0.33]**

Heterogeneity: Tau² = 0.00; Chi² = 0.24, df = 1 (P = 0.62); I² = 0%
Test for overall effect: Z = 0.13 (P = 0.89)

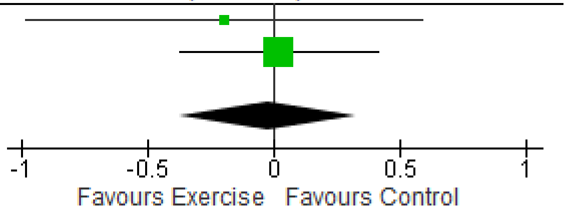

Supporting Figure: Post-intervention luteinising hormone/follicle stimulating hormone ratio; exercise versus control

Study or Subgroup	Exercise Mean	Exercise SD	Exercise Total	Control Mean	Control SD	Control Total	Weight	Mean Difference IV, Random, 95% CI
Stener-Victorin 2009	1.5	0.5	5	1.6	0.9	6	30.2%	-0.10 [-0.94, 0.74]
Turan 2015	1	0.7483	14	0.5	0.2	16	69.8%	0.50 [0.10, 0.90]
**Total (95% CI)**			**19**			**22**	**100.0%**	**0.32 [-0.22, 0.86]**

Heterogeneity: Tau² = 0.07; Chi² = 1.58, df = 1 (P = 0.21); I² = 37%
Test for overall effect: Z = 1.16 (P = 0.25)

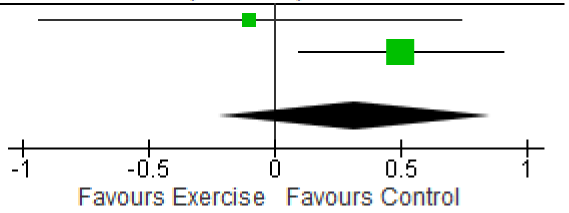

Supporting Figure: Change from baseline progesterone (nmol/L); exercise versus control

	Exercise			Control				Mean Difference
Study or Subgroup	Mean [nmol/L]	SD [nmol/L]	Total	Mean [nmol/L]	SD [nmol/L]	Total	Weight	IV, Random, 95% CI [nmol/L]
Konopka 2015	-0.954	1.272	12	0.954	3.18	13	37.7%	-1.91 [-3.78, -0.04]
Vigorito 2007	-0.1	0.4924	45	-0.1	0.7727	45	62.3%	0.00 [-0.27, 0.27]
**Total (95% CI)**			**57**			**58**	**100.0%**	**-0.72 [-2.53, 1.09]**

Heterogeneity: Tau² = 1.35; Chi² = 3.91, df = 1 (P = 0.05); I² = 74%
Test for overall effect: Z = 0.78 (P = 0.44)

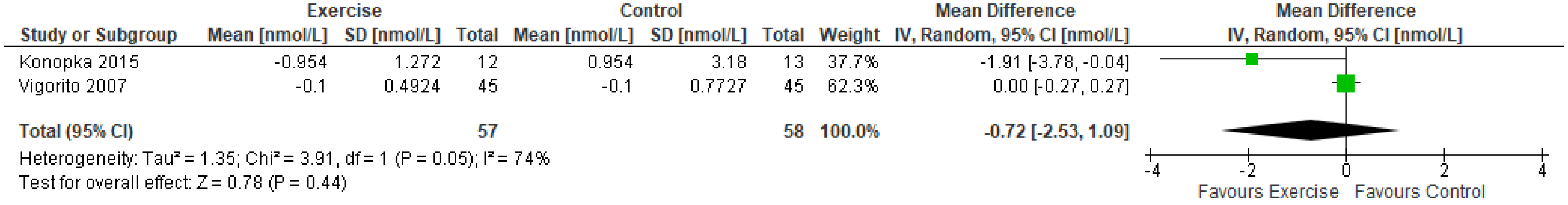

Supporting Figure: Change from baseline prolactin (ng/mL); exercise versus control

	Exercise			Control				Mean Difference
**Study or Subgroup**	**Mean [ng/ml]**	**SD [ng/ml]**	**Total**	**Mean [ng/ml]**	**SD [ng/ml]**	**Total**	**Weight**	**IV, Random, 95% CI [ng/ml]**
Vigorito 2007	-0.2	1.6844	45	-0.1	1.6156	45	93.9%	-0.10 [-0.78, 0.58]
Nasrekani 2016	0.64	3.43	10	-0.02	2.64	10	6.1%	0.66 [-2.02, 3.34]
**Total (95% CI)**			**55**			**55**	**100.0%**	**-0.05 [-0.71, 0.61]**

Heterogeneity: Tau² = 0.00; Chi² = 0.29, df = 1 (P = 0.59); I² = 0%
Test for overall effect: Z = 0.16 (P = 0.87)

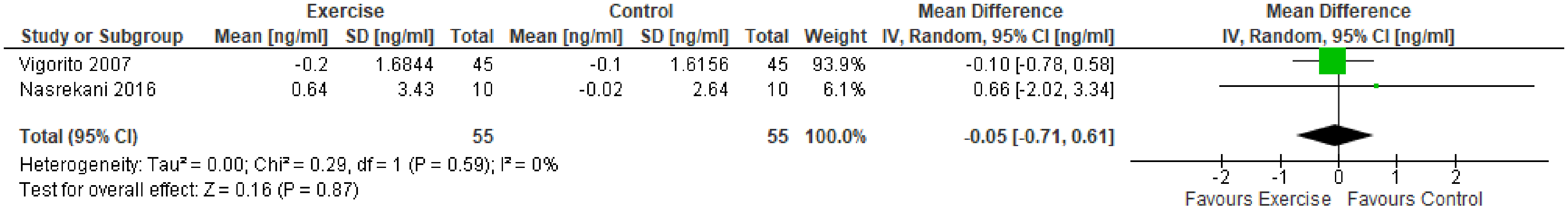

Supporting Figure: Post-intervention prolactin (ng/mL); exercise versus control

	Exercise			Control				Mean Difference
**Study or Subgroup**	**Mean [ng/ml]**	**SD [ng/ml]**	**Total**	**Mean [ng/ml]**	**SD [ng/ml]**	**Total**	**Weight**	**IV, Random, 95% CI [ng/ml]**
Vigorito 2007	10.3	1.1	45	10.1	1.2	45	99.5%	0.20 [-0.28, 0.68]
Nasrekani 2016	15.71	8.99	10	15.07	5.86	10	0.5%	0.64 [-6.01, 7.29]
**Total (95% CI)**			**55**			**55**	**100.0%**	**0.20 [-0.27, 0.68]**

Heterogeneity: Tau² = 0.00; Chi² = 0.02, df = 1 (P = 0.90); I² = 0%
Test for overall effect: Z = 0.84 (P = 0.40)

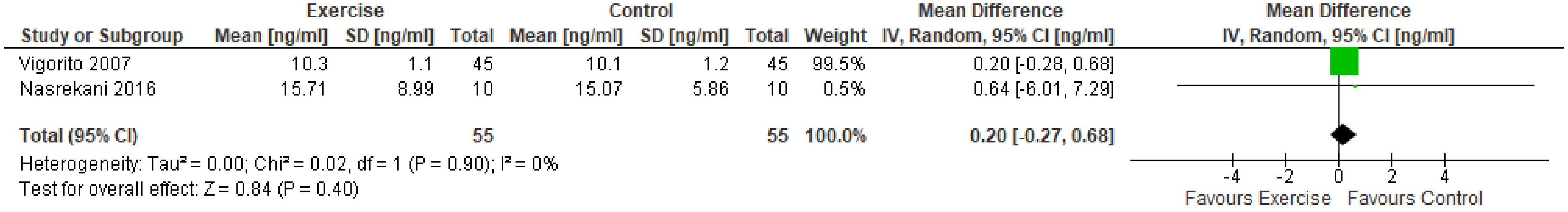

Supporting Figure: Change from baseline high-sensitivity C-reactive protein (mg/L); exercise versus control

	Exercise			Control				Mean Difference	Mean Difference
Study or Subgroup	Mean [mg/L]	SD [mg/L]	Total	Mean [mg/L]	SD [mg/L]	Total	Weight	IV, Random, 95% CI [mg/L]	IV, Random, 95% CI [mg/L]
Vizza 2016	-0.9	2.5133	7	0.3	0.3924	6	16.9%	-1.20 [-3.09, 0.69]	
Almenning 2015	-0.35	1.08	16	-0.1	1.02	9	83.1%	-0.25 [-1.10, 0.60]	
**Total (95% CI)**			**23**			**15**	**100.0%**	**-0.41 [-1.19, 0.37]**	

Heterogeneity: $Tau^2 = 0.00$; $Chi^2 = 0.81$, df = 1 (P = 0.37); $I^2 = 0\%$
Test for overall effect: Z = 1.04 (P = 0.30)

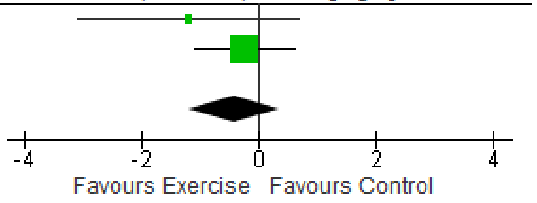

Supporting Figure: Post-intervention high-sensitivity C-reactive protein (mg/L); exercise versus control

	Exercise			Control				Mean Difference	Mean Difference
Study or Subgroup	Mean [mg/L]	SD [mg/L]	Total	Mean [mg/L]	SD [mg/L]	Total	Weight	IV, Random, 95% CI [mg/L]	IV, Random, 95% CI [mg/L]
Vizza 2016	8	10.8	7	3.9	5.4	6	4.7%	4.10 [-4.99, 13.19]	
Almenning 2015	2.1	2.63	16	1.6	2.4	9	95.3%	0.50 [-1.53, 2.53]	
**Total (95% CI)**			**23**			**15**	**100.0%**	**0.67 [-1.31, 2.65]**	

Heterogeneity: $Tau^2 = 0.00$; $Chi^2 = 0.57$, df = 1 (P = 0.45); $I^2 = 0\%$
Test for overall effect: Z = 0.66 (P = 0.51)

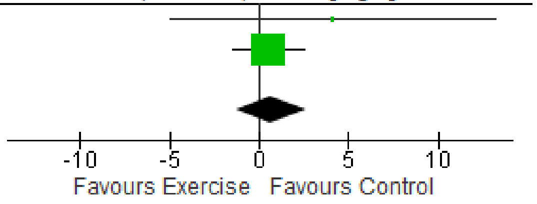

Supporting Figure: Change from baseline anti-Müllerian hormone (ng/mL); exercise versus control

	Experimental			Control				Mean Difference
**Study or Subgroup**	**Mean [ng/mL]**	**SD [ng/mL]**	**Total**	**Mean [ng/mL]**	**SD [ng/mL]**	**Total**	**Weight**	**IV, Random, 95% CI [ng/mL]**
Saremi 2013	1.49	3.27	11	0.76	2.5	11	16.4%	0.73 [-1.70, 3.16]
Saremi 2016	-0.3	2.39	10	0.38	1.79	10	28.3%	-0.68 [-2.53, 1.17]
Almenning 2015	-1.83	1.923	16	-0.756	1.4206	9	55.4%	-1.07 [-2.40, 0.25]
**Total (95% CI)**			**37**			**30**	**100.0%**	**-0.67 [-1.65, 0.32]**

Heterogeneity: $Tau^2 = 0.00$; $Chi^2 = 1.63$, df = 2 (P = 0.44); $I^2 = 0\%$
Test for overall effect: Z = 1.33 (P = 0.18)

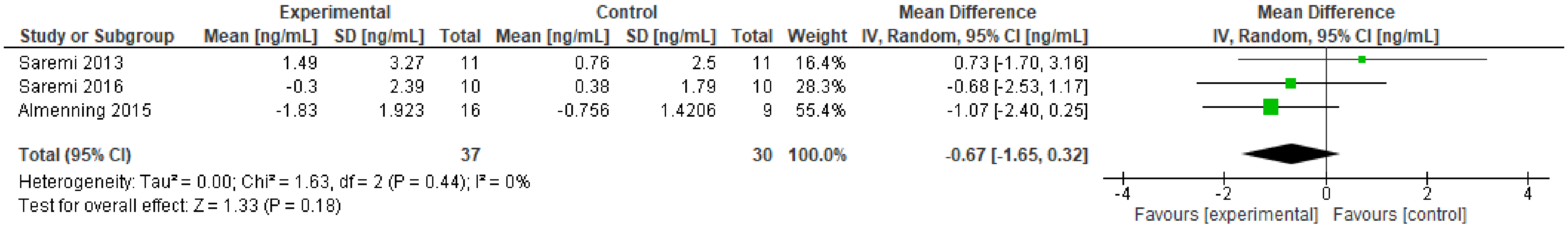

Supporting Figure: Post-intervention anti-Müllerian hormone (ng/mL); exercise versus control

	Experimental			Control				Mean Difference
**Study or Subgroup**	**Mean [ng/mL]**	**SD [ng/mL]**	**Total**	**Mean [ng/mL]**	**SD [ng/mL]**	**Total**	**Weight**	**IV, Random, 95% CI [ng/mL]**
Saremi 2013	15.93	7.74	11	17.2	6.24	11	16.2%	-1.27 [-7.15, 4.61]
Saremi 2016	14.1	5.5	10	11.6	3.9	10	32.0%	2.50 [-1.68, 6.68]
Almenning 2015	7.056	4.1566	16	7.28	3.948	9	51.8%	-0.22 [-3.51, 3.06]
**Total (95% CI)**			**37**			**30**	**100.0%**	**0.48 [-1.89, 2.84]**

Heterogeneity: $Tau^2 = 0.00$; $Chi^2 = 1.41$, df = 2 (P = 0.49); $I^2 = 0\%$
Test for overall effect: Z = 0.40 (P = 0.69)

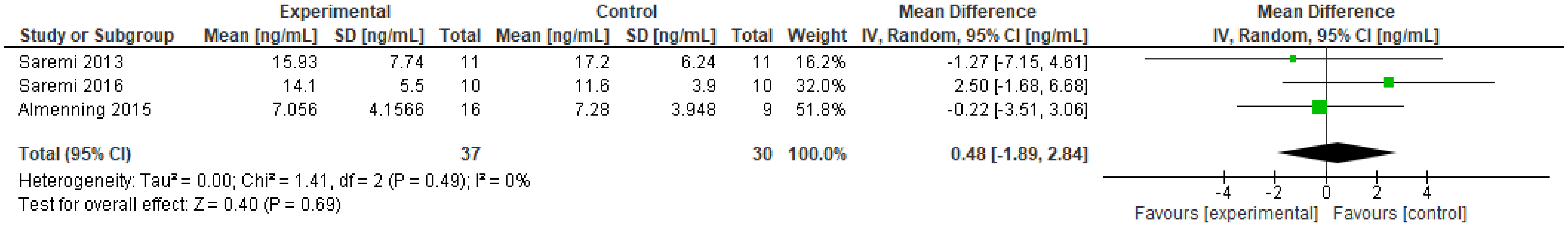

Supporting Figure: Change from baseline adiponectin (μg/mL); exercise versus control

	Exercise			Control				Mean Difference
Study or Subgroup	Mean [ug/mL]	SD [ug/mL]	Total	Mean [ug/mL]	SD [ug/mL]	Total	Weight	IV, Random, 95% CI [ug/mL]
Almenning 2015	0.2	1.53	16	0.4	1.0408	9	68.7%	-0.20 [-1.21, 0.81]
Stener-Victorin 2009	0.59	2.61	30	0.78	2.32	15	31.3%	-0.19 [-1.69, 1.31]
**Total (95% CI)**			**46**			**24**	**100.0%**	**-0.20 [-1.04, 0.64]**

Heterogeneity: $Tau^2 = 0.00$; $Chi^2 = 0.00$, $df = 1$ ($P = 0.99$); $I^2 = 0\%$

Test for overall effect: $Z = 0.46$ ($P = 0.65$)

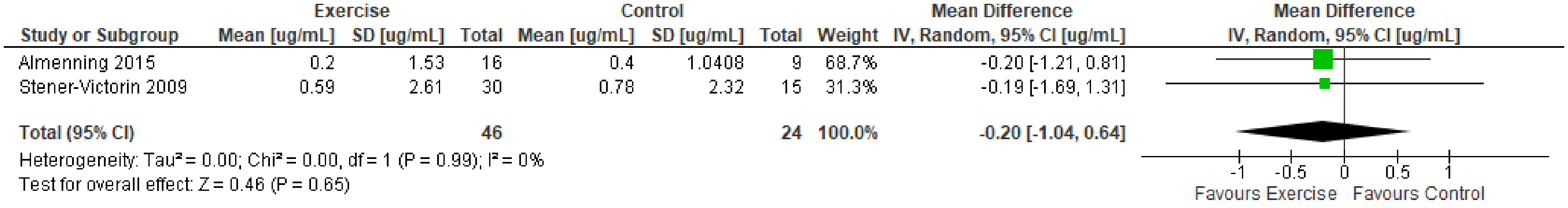

Supporting Figure: Change from baseline PCOS-Q domains; exercise versus control

Study or Subgroup	Exercise Mean	Exercise SD	Exercise Total	Control Mean	Control SD	Control Total	Weight	Mean Difference IV, Random, 95% CI
**8.51.1 Emotions**								
Vizza 2016	0.7	0.5893	7	-0.1	0.1353	6	29.9%	0.80 [0.35, 1.25]
**Subtotal (95% CI)**			**7**			**6**	**29.9%**	**0.80 [0.35, 1.25]**
Heterogeneity: Not applicable								
Test for overall effect: Z = 3.49 (P = 0.0005)								
**8.51.2 Body Hair**								
Vizza 2016	-0.4	1.0922	7	0	1.2002	6	14.4%	-0.40 [-1.66, 0.86]
**Subtotal (95% CI)**			**7**			**6**	**14.4%**	**-0.40 [-1.66, 0.86]**
Heterogeneity: Not applicable								
Test for overall effect: Z = 0.62 (P = 0.53)								
**8.51.3 Body Weight**								
Vizza 2016	0.8	0.915	7	-0.1	0.6	6	21.6%	0.90 [0.07, 1.73]
**Subtotal (95% CI)**			**7**			**6**	**21.6%**	**0.90 [0.07, 1.73]**
Heterogeneity: Not applicable								
Test for overall effect: Z = 2.12 (P = 0.03)								
**8.51.4 Infertility**								
Vizza 2016	2.6	1.8555	7	0.8	1.0098	6	10.5%	1.80 [0.21, 3.39]
**Subtotal (95% CI)**			**7**			**6**	**10.5%**	**1.80 [0.21, 3.39]**
Heterogeneity: Not applicable								
Test for overall effect: Z = 2.21 (P = 0.03)								
**8.51.5 Menstruation**								
Vizza 2016	-0.2	0.7746	7	0	0.57	6	23.6%	-0.20 [-0.93, 0.53]
**Subtotal (95% CI)**			**7**			**6**	**23.6%**	**-0.20 [-0.93, 0.53]**
Heterogeneity: Not applicable								
Test for overall effect: Z = 0.53 (P = 0.59)								
**Total (95% CI)**			**35**			**30**	**100.0%**	**0.52 [-0.10, 1.14]**

Heterogeneity: Tau² = 0.28; Chi² = 10.43, df = 4 (P = 0.03); I² = 62%
Test for overall effect: Z = 1.65 (P = 0.10)
Test for subgroup differences: Chi² = 10.43, df = 4 (P = 0.03), I² = 61.6%

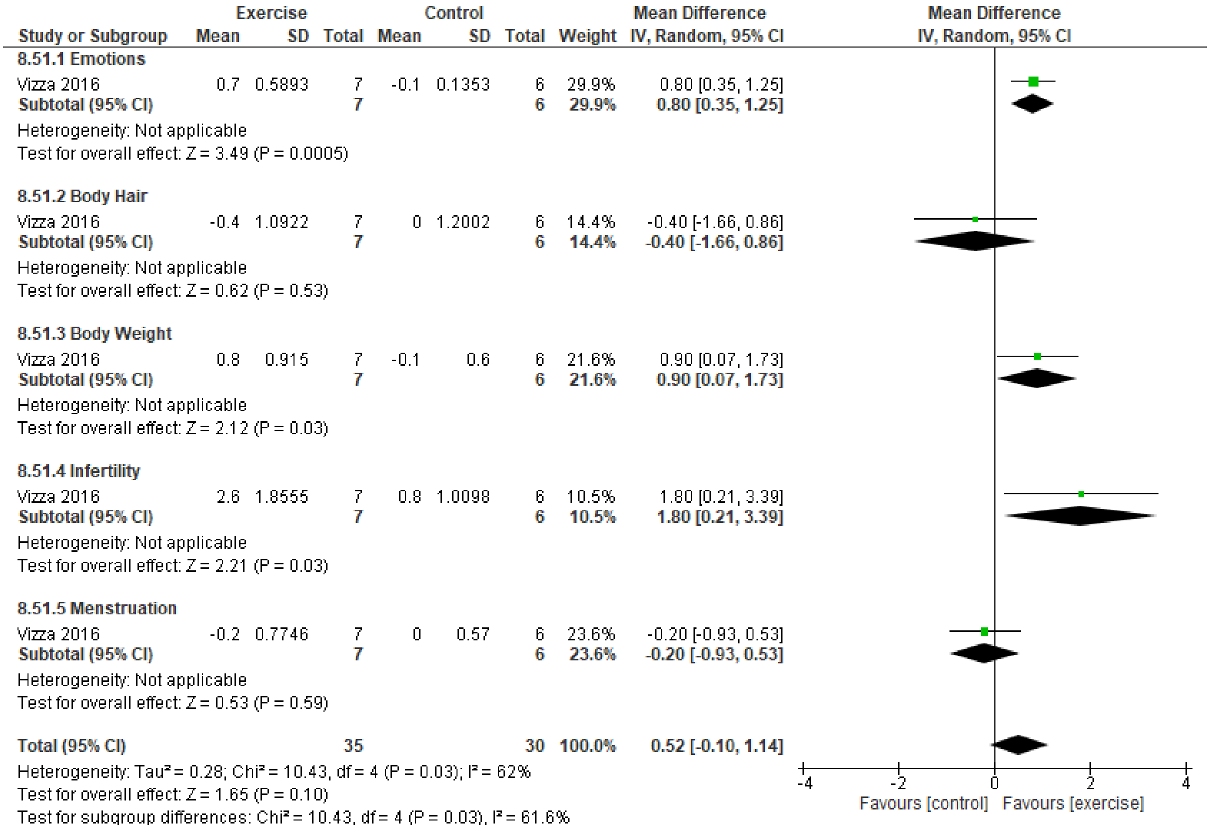

<u>**Supplementary Meta-Analyses – Exercise and Diet versus Diet**</u>

Supporting Figure: Change from baseline fasting blood glucose (mg/dL); exercise and diet versus diet

	Combined			Diet Only				Mean Difference	Mean Difference
**Study or Subgroup**	**Mean [mg/dL]**	**SD [mg/dL]**	**Total**	**Mean [mg/dL]**	**SD [mg/dL]**	**Total**	**Weight**	**IV, Random, 95% CI [mg/dL]**	**IV, Random, 95% CI [mg/dL]**
Nybacka 2011	-2.89	6.5316	12	-3.17	6.8759	14	29.1%	0.28 [-4.88, 5.44]	
Thomson 2008	-3	2.18	38	-7	3.8	14	70.9%	4.00 [1.89, 6.11]	
**Total (95% CI)**			**50**			**28**	**100.0%**	**2.92 [-0.40, 6.23]**	

Heterogeneity: Tau² = 2.87; Chi² = 1.71, df = 1 (P = 0.19); I² = 42%
Test for overall effect: Z = 1.73 (P = 0.08)

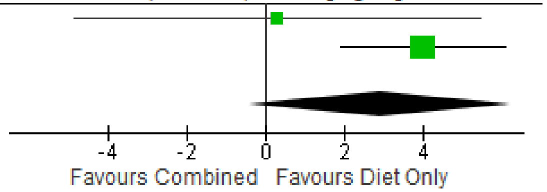

Supporting Figure: Post-intervention fasting blood glucose (mg/dL); exercise and diet versus diet

	Combined			Diet Only				Mean Difference	Mean Difference
**Study or Subgroup**	**Mean [mg/dL]**	**SD [mg/dL]**	**Total**	**Mean [mg/dL]**	**SD [mg/dL]**	**Total**	**Weight**	**IV, Random, 95% CI [mg/dL]**	**IV, Random, 95% CI [mg/dL]**
Thomson 2008	90	10	38	89	11	14	45.2%	1.00 [-5.58, 7.58]	
Nybacka 2011	83.8	8.2	12	79.4	7.2	14	54.8%	4.40 [-1.58, 10.38]	
**Total (95% CI)**			**50**			**28**	**100.0%**	**2.86 [-1.56, 7.29]**	

Heterogeneity: Tau² = 0.00; Chi² = 0.56, df = 1 (P = 0.45); I² = 0%
Test for overall effect: Z = 1.27 (P = 0.20)

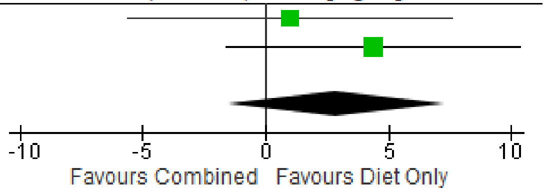

Supporting Figure: Change from baseline fasting insulin (µIU/mL); exercise and diet versus diet

Study or Subgroup	Combined Mean [uIU/mL]	Combined SD [uIU/mL]	Combined Total	Diet Only Mean [uIU/mL]	Diet Only SD [uIU/mL]	Diet Only Total	Weight	Mean Difference IV, Random, 95% CI [uIU/mL]
Bruner 2006	-4.92	6.78	7	-18.54	11.69	5	18.2%	13.62 [2.21, 25.03]
Nybacka 2011	-3.45	7.476	12	-2.85	7.7592	14	36.2%	-0.60 [-6.47, 5.27]
Thomson 2008	-4.28	1.99	38	-4.2	7.1	14	45.7%	-0.08 [-3.85, 3.69]
**Total (95% CI)**			**57**			**33**	**100.0%**	**2.22 [-3.70, 8.14]**

Heterogeneity: Tau² = 16.27; Chi² = 5.28, df = 2 (P = 0.07); I² = 62%
Test for overall effect: Z = 0.74 (P = 0.46)

Mean Difference IV, Random, 95% CI [uIU/mL]

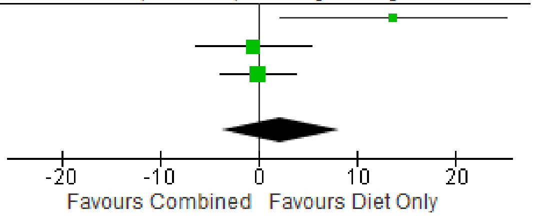

Supporting Figure: Post-intervention fasting insulin (µIU/mL); exercise and diet versus diet

Study or Subgroup	Combined Mean [uIU/mL]	Combined SD [uIU/mL]	Combined Total	Diet Only Mean [uIU/mL]	Diet Only SD [uIU/mL]	Diet Only Total	Weight	Mean Difference IV, Random, 95% CI [uIU/mL]
Bruner 2006	11.88	7.9108	7	15.12	7.9157	5	30.1%	-3.24 [-12.32, 5.84]
Thomson 2008	11.01	9.22	38	13.5	9.9	14	69.9%	-2.49 [-8.45, 3.47]
**Total (95% CI)**			**45**			**19**	**100.0%**	**-2.72 [-7.70, 2.27]**

Heterogeneity: Tau² = 0.00; Chi² = 0.02, df = 1 (P = 0.89); I² = 0%
Test for overall effect: Z = 1.07 (P = 0.29)

Mean Difference IV, Random, 95% CI [uIU/mL]

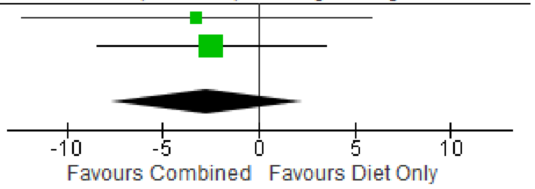

Supporting Figure: Change from baseline HOMA-IR; exercise and diet versus diet

	Combined			Diet Only				Mean Difference
**Study or Subgroup**	**Mean**	**SD**	**Total**	**Mean**	**SD**	**Total**	**Weight**	**IV, Random, 95% CI**
Nybacka 2011	-0.9	1.6998	12	-0.74	1.7666	14	10.9%	-0.16 [-1.49, 1.17]
Thomson 2008	-0.55	0.24	38	-0.56	0.88	14	89.1%	0.01 [-0.46, 0.48]
**Total (95% CI)**			**50**			**28**	**100.0%**	**-0.01 [-0.45, 0.43]**

Heterogeneity: Tau² = 0.00; Chi² = 0.06, df = 1 (P = 0.81); I² = 0%
Test for overall effect: Z = 0.04 (P = 0.97)

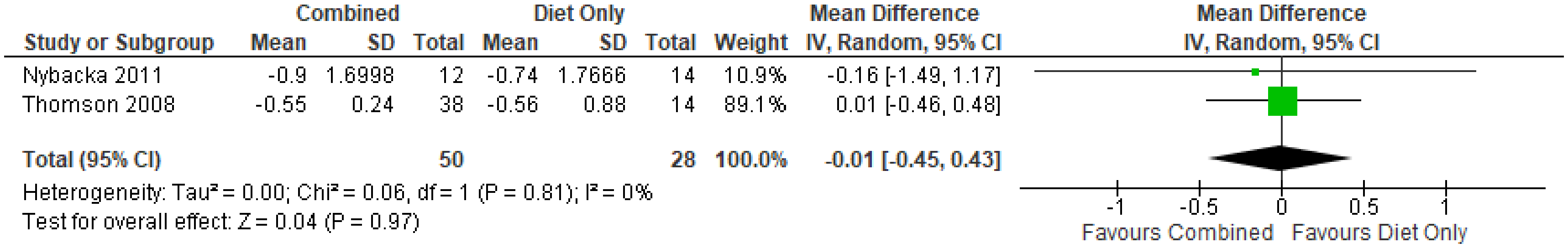

Supporting Figure: Change from baseline body mass (kg); exercise and diet versus diet

Study or Subgroup	Combined Mean [kg]	Combined SD [kg]	Combined Total	Diet Only Mean [kg]	Diet Only SD [kg]	Diet Only Total	Weight	Mean Difference IV, Random, 95% CI [kg]
Bruner 2006	-0.8	10.06	7	-3.1	7.9	5	10.1%	2.30 [-7.87, 12.47]
Thomson 2008	-9.31	4.21	38	-8.6	6	14	89.9%	-0.71 [-4.13, 2.71]
**Total (95% CI)**			**45**			**19**	**100.0%**	**-0.40 [-3.64, 2.83]**

Heterogeneity: Tau² = 0.00; Chi² = 0.30, df = 1 (P = 0.58); I² = 0%
Test for overall effect: Z = 0.25 (P = 0.81)

Mean Difference
IV, Random, 95% CI [kg]

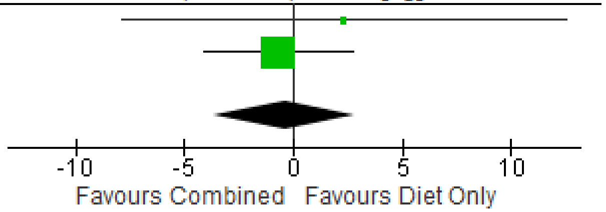

Supporting Figure: Post-intervention body mass (kg); exercise and diet versus diet

Study or Subgroup	Combined Mean [kg]	Combined SD [kg]	Combined Total	Diet Only Mean [kg]	Diet Only SD [kg]	Diet Only Total	Weight	Mean Difference IV, Random, 95% CI [kg]
Bruner 2006	99.7	19.84	7	91.7	10.96	5	29.5%	8.00 [-9.56, 25.56]
Thomson 2008	90.66	18.4	38	91.9	18.6	14	70.5%	-1.24 [-12.60, 10.12]
**Total (95% CI)**			**45**			**19**	**100.0%**	**1.49 [-8.05, 11.03]**

Heterogeneity: Tau² = 0.00; Chi² = 0.75, df = 1 (P = 0.39); I² = 0%
Test for overall effect: Z = 0.31 (P = 0.76)

Mean Difference
IV, Random, 95% CI [kg]

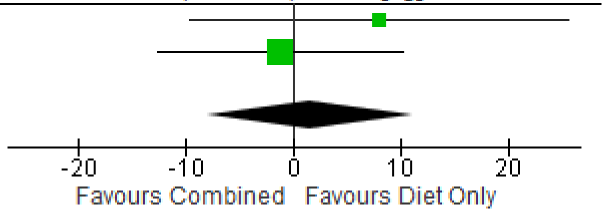

Supporting Figure: Change from baseline body mass index (kg/m^2); exercise and diet versus diet

	Combined			Diet Only				Mean Difference
Study or Subgroup	Mean	SD	Total	Mean	SD	Total	Weight	IV, Random, 95% CI
Bruner 2006	-0.3	2.97	7	-1.2	4.53	5	6.7%	0.90 [-3.64, 5.44]
Nybacka 2011	-1.9	1.5739	12	-1.74	1.5934	14	93.3%	-0.16 [-1.38, 1.06]
**Total (95% CI)**			**19**			**19**	**100.0%**	**-0.09 [-1.27, 1.09]**

Heterogeneity: Tau² = 0.00; Chi² = 0.20, df = 1 (P = 0.66); I² = 0%
Test for overall effect: Z = 0.15 (P = 0.88)

Mean Difference
IV, Random, 95% CI

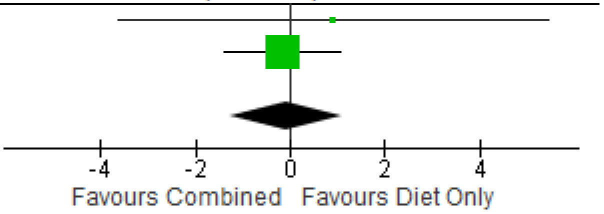

Supporting Figure: Post-intervention body mass index (kg/m^2); exercise and diet versus diet

	Combined			Diet Only				Mean Difference
Study or Subgroup	Mean	SD	Total	Mean	SD	Total	Weight	IV, Random, 95% CI
Bruner 2006	35.9	5.82	7	35.9	6.71	5	35.1%	0.00 [-7.29, 7.29]
Nybacka 2011	36.9	8	12	32.96	5.5	14	64.9%	3.94 [-1.43, 9.31]
**Total (95% CI)**			**19**			**19**	**100.0%**	**2.56 [-1.77, 6.88]**

Heterogeneity: Tau² = 0.00; Chi² = 0.73, df = 1 (P = 0.39); I² = 0%
Test for overall effect: Z = 1.16 (P = 0.25)

Mean Difference
IV, Random, 95% CI

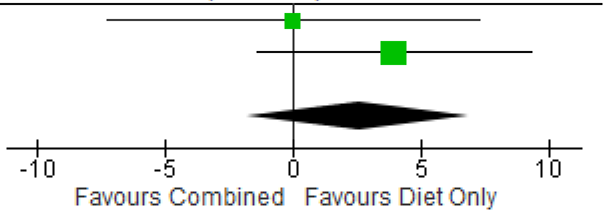

Supporting Figure: Change from baseline waist circumference (cm); exercise and diet versus diet

	Combined			Diet Only				Mean Difference
Study or Subgroup	Mean [cm]	SD [cm]	Total	Mean [cm]	SD [cm]	Total	Weight	IV, Random, 95% CI [cm]
Bruner 2006	-5.2	6.93	7	-5	7.36	5	17.8%	-0.20 [-8.44, 8.04]
Thomson 2008	-11.33	2.95	38	-10.8	7.1	14	82.2%	-0.53 [-4.37, 3.31]
**Total (95% CI)**			**45**			**19**	**100.0%**	**-0.47 [-3.95, 3.01]**

Heterogeneity: Tau² = 0.00; Chi² = 0.01, df = 1 (P = 0.94); I² = 0%
Test for overall effect: Z = 0.27 (P = 0.79)

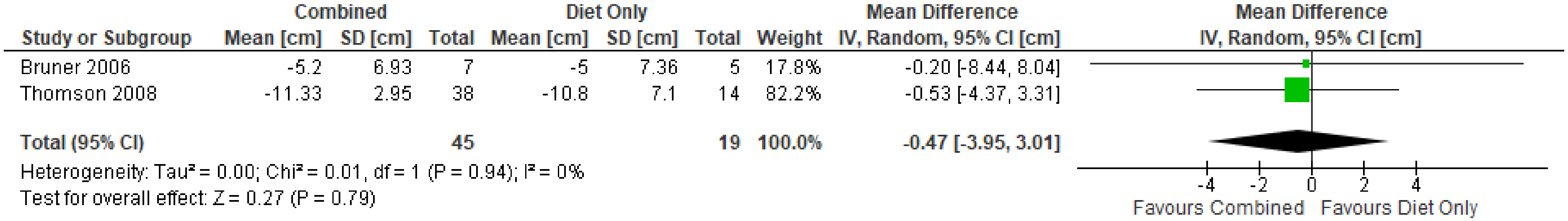

Supporting Figure: Post-intervention waist circumference (cm); exercise and diet versus diet

	Combined			Diet Only				Mean Difference
Study or Subgroup	Mean [cm]	SD [cm]	Total	Mean [cm]	SD [cm]	Total	Weight	IV, Random, 95% CI [cm]
Bruner 2006	93.1	12.7	7	94.8	12.07	5	25.7%	-1.70 [-15.86, 12.46]
Thomson 2008	90.76	13.29	38	92.2	13.7	14	74.3%	-1.44 [-9.77, 6.89]
**Total (95% CI)**			**45**			**19**	**100.0%**	**-1.51 [-8.69, 5.67]**

Heterogeneity: Tau² = 0.00; Chi² = 0.00, df = 1 (P = 0.98); I² = 0%
Test for overall effect: Z = 0.41 (P = 0.68)

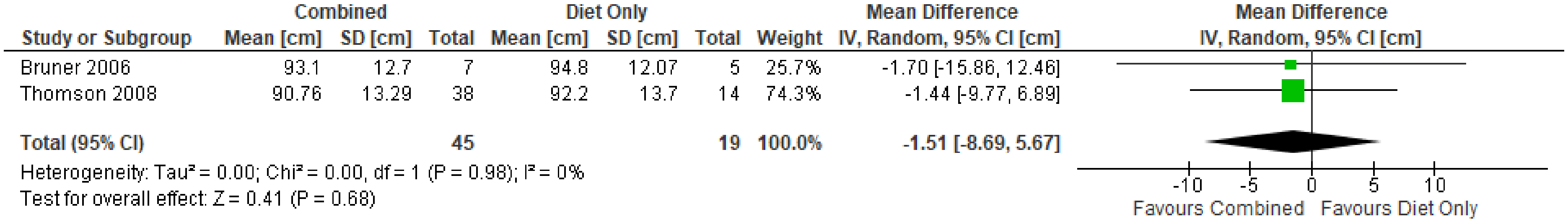

Supporting Figure: Change from baseline body fat percentage (%); exercise and diet versus diet

	Combined			Diet Only				Mean Difference
Study or Subgroup	Mean [%]	SD [%]	Total	Mean [%]	SD [%]	Total	Weight	IV, Random, 95% CI [%]
Nybacka 2011	-0.84	2.7858	12	-1.66	2.8231	14	48.4%	0.82 [-1.34, 2.98]
Thomson 2008	-3.71	0.97	38	-0.9	3.3	14	51.6%	-2.81 [-4.57, -1.05]
**Total (95% CI)**			**50**			**28**	**100.0%**	**-1.05 [-4.61, 2.50]**

Heterogeneity: Tau² = 5.58; Chi² = 6.53, df = 1 (P = 0.01); I² = 85%
Test for overall effect: Z = 0.58 (P = 0.56)

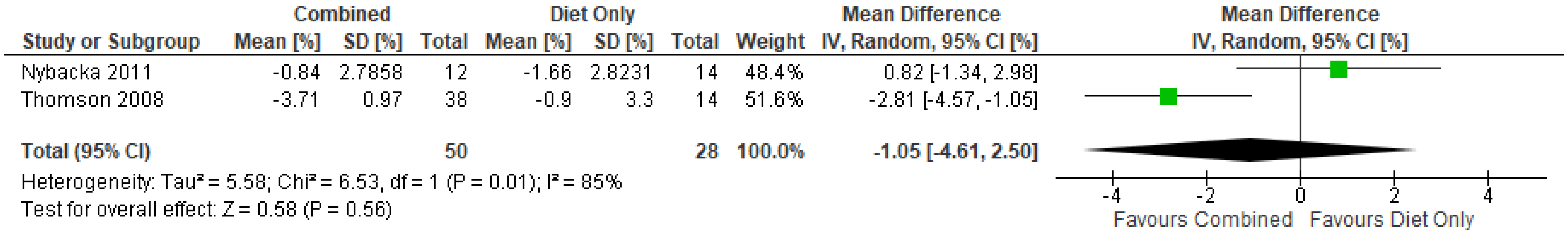

Supporting Figure: Post-intervention body fat percentage (%); exercise and diet versus diet

	Combined			Diet Only				Mean Difference
Study or Subgroup	Mean [%]	SD [%]	Total	Mean [%]	SD [%]	Total	Weight	IV, Random, 95% CI [%]
Nybacka 2011	45.2	6.4	12	44	6	14	29.6%	1.20 [-3.59, 5.99]
Thomson 2008	43.98	4.53	38	45.8	4.9	14	70.4%	-1.82 [-4.76, 1.12]
**Total (95% CI)**			**50**			**28**	**100.0%**	**-0.93 [-3.63, 1.77]**

Heterogeneity: Tau² = 0.44; Chi² = 1.11, df = 1 (P = 0.29); I² = 10%
Test for overall effect: Z = 0.67 (P = 0.50)

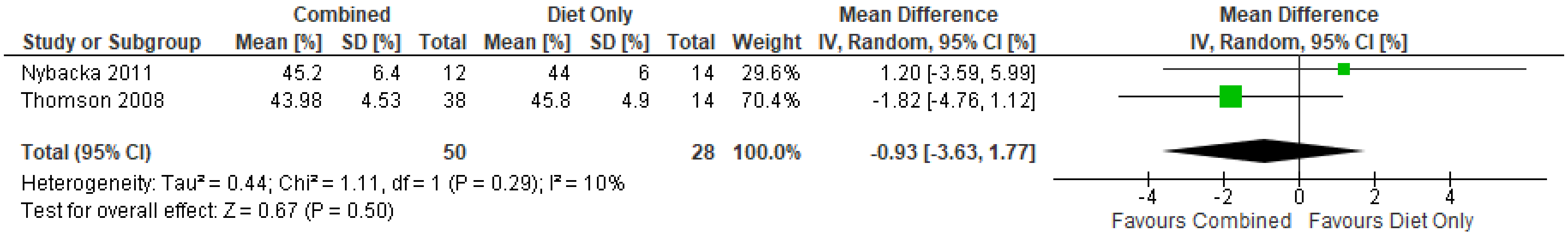

Supporting Figure: Change from baseline fat free mass (kg); exercise and diet versus diet

	Combined			Diet Only				Mean Difference
Study or Subgroup	Mean [kg]	SD [kg]	Total	Mean [kg]	SD [kg]	Total	Weight	IV, Random, 95% CI [kg]
Thomson 2008	-1.52	2.06	38	-3.8	3.8	14	49.2%	2.28 [0.18, 4.38]
Nybacka 2011	-2.66	2.3294	12	-1.23	2.5806	14	50.8%	-1.43 [-3.32, 0.46]
**Total (95% CI)**			**50**			**28**	**100.0%**	**0.40 [-3.24, 4.03]**

Heterogeneity: Tau² = 5.85; Chi² = 6.65, df = 1 (P = 0.010); I² = 85%
Test for overall effect: Z = 0.21 (P = 0.83)

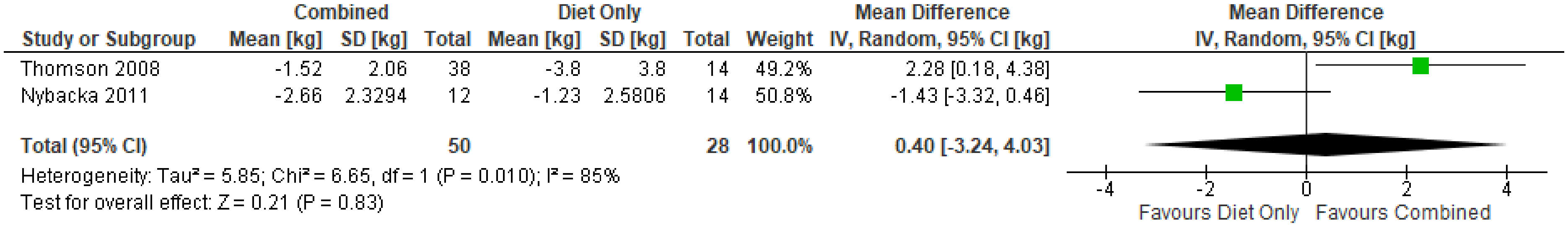

Supporting Figure: Post-intervention fat free mass (kg); exercise and diet versus diet

	Combined			Diet Only				Mean Difference
Study or Subgroup	Mean [kg]	SD [kg]	Total	Mean [kg]	SD [kg]	Total	Weight	IV, Random, 95% CI [kg]
Thomson 2008	50.24	8.97	38	49.3	9.3	14	45.1%	0.94 [-4.70, 6.58]
Nybacka 2011	49.7	7.6	12	46.7	5.3	14	54.9%	3.00 [-2.12, 8.12]
**Total (95% CI)**			**50**			**28**	**100.0%**	**2.07 [-1.72, 5.86]**

Heterogeneity: Tau² = 0.00; Chi² = 0.28, df = 1 (P = 0.60); I² = 0%
Test for overall effect: Z = 1.07 (P = 0.28)

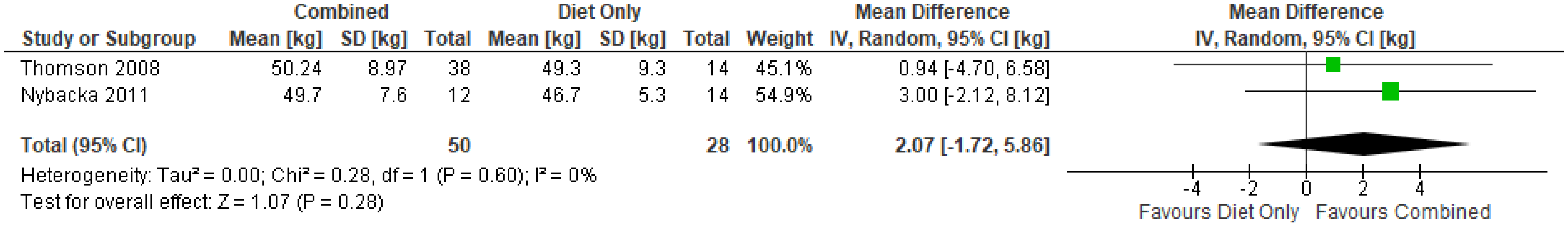

Supporting Figure: Change from baseline total testosterone (nmol/L); exercise and diet versus diet

	Combined			Diet Only				Mean Difference
Study or Subgroup	Mean [nmol/L]	SD [nmol/L]	Total	Mean [nmol/L]	SD [nmol/L]	Total	Weight	IV, Random, 95% CI [nmol/L]
Bruner 2006	0.2	0.36	7	0	1.07	5	26.2%	0.20 [-0.78, 1.18]
Nybacka 2011	-0.21	0.86	12	-1.16	0.82	14	33.8%	0.95 [0.30, 1.60]
Thomson 2008	-0.47	0.18	38	-0.27	0.71	14	40.0%	-0.20 [-0.58, 0.18]
**Total (95% CI)**			**57**			**33**	**100.0%**	**0.29 [-0.49, 1.08]**

Heterogeneity: Tau² = 0.36; Chi² = 9.07, df = 2 (P = 0.01); I² = 78%
Test for overall effect: Z = 0.74 (P = 0.46)

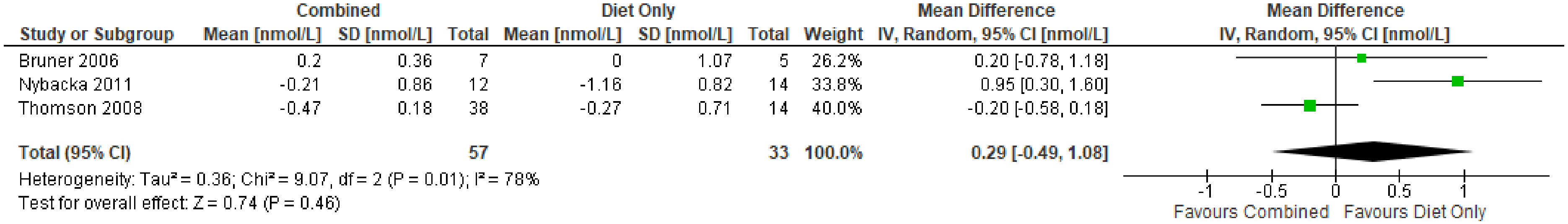

Supporting Figure: Post-intervention total testosterone (nmol/L); exercise and diet versus diet

	Combined			Diet Only				Mean Difference
Study or Subgroup	Mean [nmol/L]	SD [nmol/L]	Total	Mean [nmol/L]	SD [nmol/L]	Total	Weight	IV, Random, 95% CI [nmol/L]
Nybacka 2011	4.74	2	12	4.21	2	14	8.9%	0.53 [-1.01, 2.07]
Bruner 2006	3.2	0.53	7	3.1	0.89	5	27.9%	0.10 [-0.77, 0.97]
Thomson 2008	2.1	0.85	38	2.09	0.98	14	63.2%	0.01 [-0.57, 0.59]
**Total (95% CI)**			**57**			**33**	**100.0%**	**0.08 [-0.38, 0.54]**

Heterogeneity: Tau² = 0.00; Chi² = 0.39, df = 2 (P = 0.82); I² = 0%
Test for overall effect: Z = 0.35 (P = 0.73)

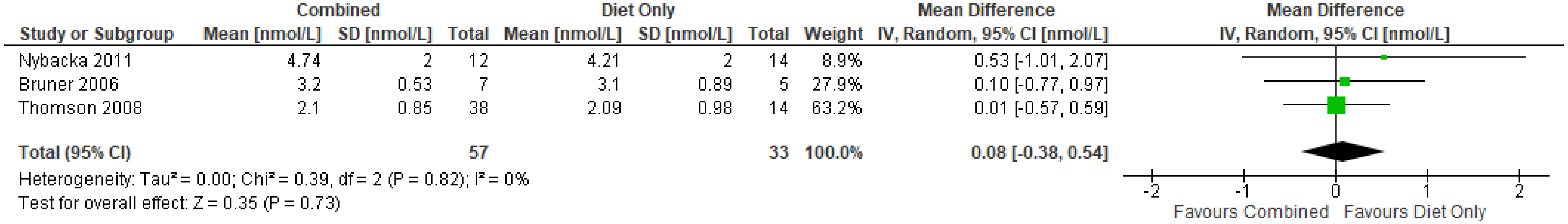

Supporting Figure: Change from baseline sex hormone binding globulin (nmol/L); exercise and diet versus diet

	Combined			Diet Only				Mean Difference
Study or Subgroup	Mean [nmol/L]	SD [nmol/L]	Total	Mean [nmol/L]	SD [nmol/L]	Total	Weight	IV, Random, 95% CI [nmol/L]
Bruner 2006	10.4	13.98	7	1.3	6.32	5	15.6%	9.10 [-2.64, 20.84]
Thomson 2008	8.33	3.76	38	4.2	12.6	14	31.9%	4.13 [-2.58, 10.84]
Nybacka 2011	3.15	4.52	12	4.21	3.6	14	52.5%	-1.06 [-4.24, 2.12]
**Total (95% CI)**			**57**			**33**	**100.0%**	**2.18 [-3.15, 7.51]**

Heterogeneity: Tau² = 11.44; Chi² = 4.11, df = 2 (P = 0.13); I² = 51%
Test for overall effect: Z = 0.80 (P = 0.42)

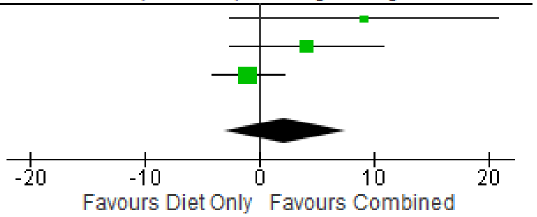

Supporting Figure: Post-intervention sex hormone binding globulin (nmol/L); exercise and diet versus diet

	Combined			Diet Only				Mean Difference
Study or Subgroup	Mean [nmol/L]	SD [nmol/L]	Total	Mean [nmol/L]	SD [nmol/L]	Total	Weight	IV, Random, 95% CI [nmol/L]
Bruner 2006	36.8	35.19	7	18.4	8.94	5	14.6%	18.40 [-8.82, 45.62]
Thomson 2008	43.27	17.02	38	31.5	17.5	14	40.2%	11.77 [1.13, 22.41]
Nybacka 2011	25.25	11.58	12	27.37	10.53	14	45.3%	-2.12 [-10.68, 6.44]
**Total (95% CI)**			**57**			**33**	**100.0%**	**6.45 [-5.52, 18.42]**

Heterogeneity: Tau² = 63.36; Chi² = 5.08, df = 2 (P = 0.08); I² = 61%
Test for overall effect: Z = 1.06 (P = 0.29)

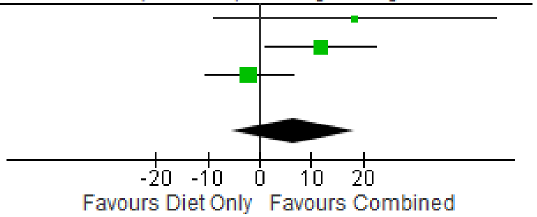

Supporting Figure: Change from baseline free androgen index; exercise and diet versus diet

	Combined			Diet Only				Mean Difference
**Study or Subgroup**	**Mean**	**SD**	**Total**	**Mean**	**SD**	**Total**	**Weight**	**IV, Random, 95% CI**
Bruner 2006	0	3.68	7	-0.5	6.16	5	15.6%	0.50 [-5.55, 6.55]
Thomson 2008	-2.76	1.38	38	-2.8	4.9	14	84.4%	0.04 [-2.56, 2.64]
**Total (95% CI)**			**45**			**19**	**100.0%**	**0.11 [-2.28, 2.50]**

Heterogeneity: Tau² = 0.00; Chi² = 0.02, df = 1 (P = 0.89); I² = 0%
Test for overall effect: Z = 0.09 (P = 0.93)

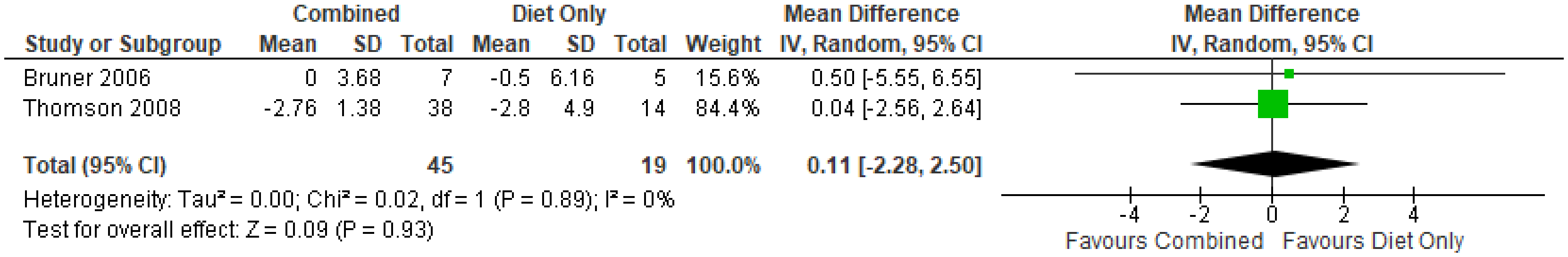

Supporting Figure: Post-intervention free androgen index; exercise and diet versus diet

	Combined			Diet Only				Mean Difference
**Study or Subgroup**	**Mean**	**SD**	**Total**	**Mean**	**SD**	**Total**	**Weight**	**IV, Random, 95% CI**
Bruner 2006	13.9	6.879	7	19.84	9.1679	5	15.1%	-5.94 [-15.46, 3.58]
Thomson 2008	6.06	6.41	38	8.4	6.6	14	84.9%	-2.34 [-6.35, 1.67]
**Total (95% CI)**			**45**			**19**	**100.0%**	**-2.88 [-6.58, 0.81]**

Heterogeneity: Tau² = 0.00; Chi² = 0.47, df = 1 (P = 0.49); I² = 0%
Test for overall effect: Z = 1.53 (P = 0.13)

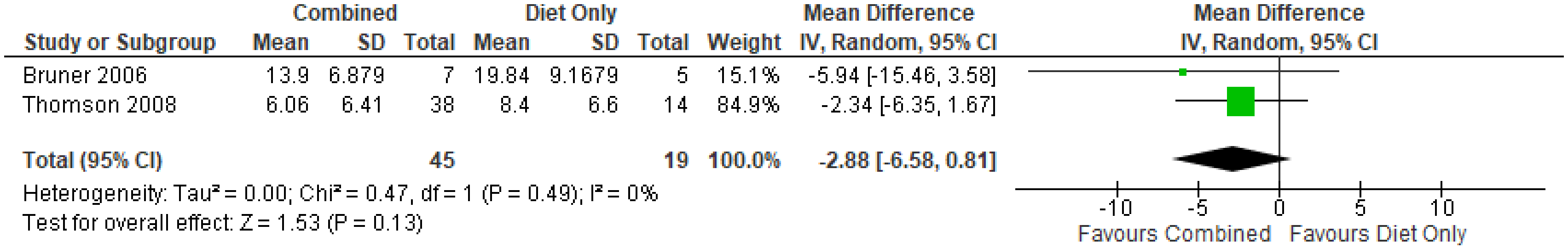

Supplement: Supplementary file 4 — Additional meta-analyses/complete dataset. Supplementary meta-analyses: exercise vs. control; exercise and diet versus diet. (PDF 913 kb) [file 13643_2019_962_MOESM4_ESM.pdf]
